# Potentiometric Sensing of Nonsteroidal Painkillers by Acyclic Squaramide Ionophores

**DOI:** 10.1021/acssensors.3c00981

**Published:** 2023-08-02

**Authors:** Giacomo Picci, Sara Farotto, Jessica Milia, Claudia Caltagirone, Vito Lippolis, Maria Carla Aragoni, Corrado Di Natale, Roberto Paolesse, Larisa Lvova

**Affiliations:** † Dipartimento di Scienze Chimiche e Geologiche, Università degli Studi di Cagliari, S.S. 554 Bivio per Sestu, 09042 Monserrato (CA), Italy; ‡ Department of Chemical Science and Technologies, University of Rome “Tor Vergata”, 00133 Rome, Italy; § Department of Electronic Engineering, University of Rome “Tor Vergata”, 00133 Rome, Italy

**Keywords:** squaramides, potentiometric sensing ion-selective electrodes, anion recognition, emerging pollutants, supramolecular chemistry

## Abstract

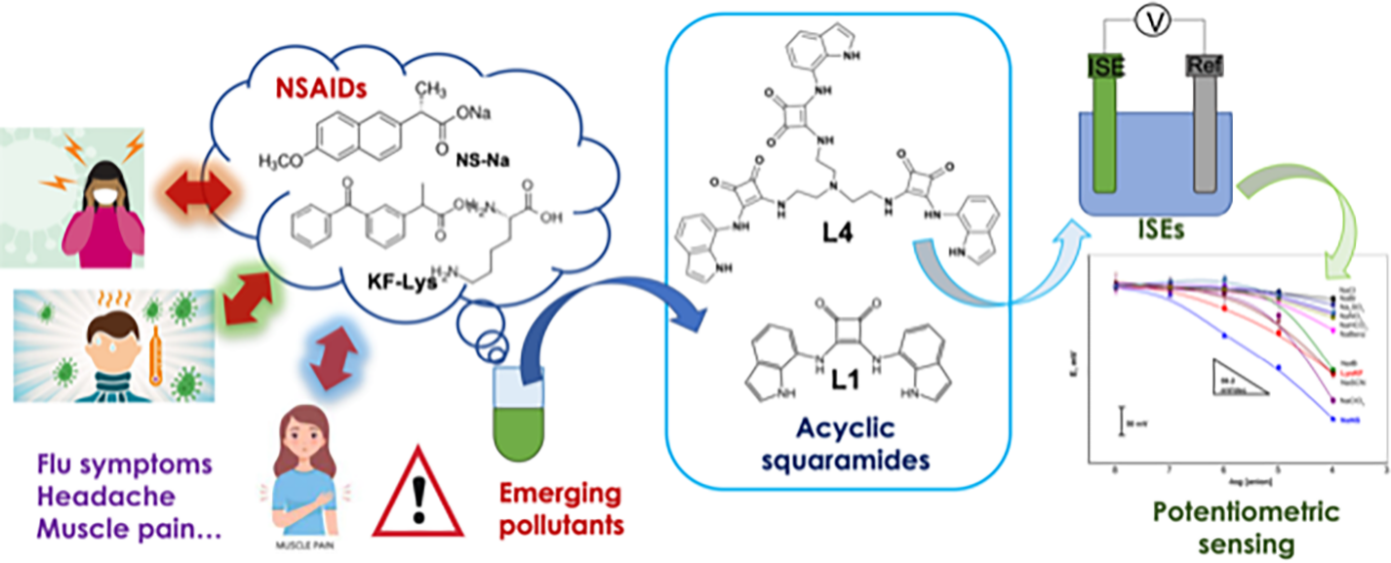

We report here a small library of a new type of acyclic squaramide receptors (**L1**–**L5**) as selective ionophores for the detection of ketoprofen and naproxen anions (KF^–^ and NS^–^, respectively) in aqueous media. ^1^H NMR binding studies show a high affinity of these squaramide receptors toward KF^–^ and NS^–^, suggesting the formation of H-bonds between the two guests and the receptors through indole and −NH groups. Compounds **L1**–**L5** have been tested as ionophores for the detection of KF^–^ and NS^–^ inside solvent PVC-based polymeric membranes. The optimal membrane compositions were established through the careful variation of the ligand/tridodecylmethylammonium chloride (TDMACl) anion-exchanger ratio. All of the tested acyclic squaramide receptors **L1**–**L5** have high affinity toward KF^–^ and NS^–^ and anti-Hofmeister selectivity, with **L4** and **L5** showing the highest sensitivity and selectivity to NS^–^. The utility of the developed sensors for a high precision detection of KF^–^ in pharmaceutical compositions with low relative errors of analysis (RSD, 0.99–1.4%) and recoveries, *R*%, in the range 95.1–111.8% has been demonstrated. Additionally, the chemometric approach has been involved to effectively discriminate between the structurally very similar KF^–^ and NS^–^, and the possibility of detecting these analytes at concentrations as low as 0.07 μM with *R*^2^ of 0.947 and at 0.15 μM with *R*^2^ of 0.919 for NS^–^ and KF^–^, respectively, was shown.

Nonsteroidal anti-inflammatory drugs (NSAIDs) are rapidly becoming emerging pollutants due to their continuously growing use and wide application to relieve headache, muscular, and other long-term courses of pain, reduce inflammation, fever, and other symptoms of colds and flu, and fight against mild COVID-19 symptoms.^1^ Among NSAIDs, ketoprofen (KF) and naproxen (NS) are widely used in many pharmaceutical compositions for both adults and children. These two compounds have a relatively simple chemical structure; in pharmacological compositions, they are often introduced as the more water-soluble salts Lys-KF (ketoprofen lysine salt) and NaNS (Scheme 1). Like any drug, besides a direct anti-inflammatory action, NSAIDs may have several undesirable side effects on a patient’s health, especially when undergoing high-dose therapies or during long-term medications, such as indigestion and diarrhea, allergic reactions, stomach ulcerous, and liver and kidney dysfunctions, making it important to control the intake of NSAIDs.^2^

**Scheme 1:**
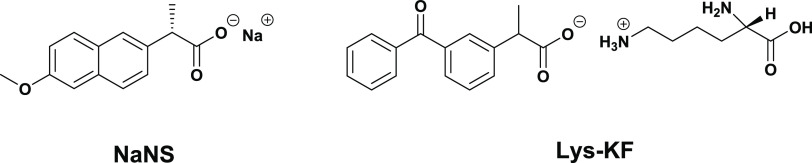
Chemical Structures of Sodium Naproxen, NaNS, and Ketoprofen Lysine, Lys-KF

Moreover, being toxic for biota, NSAIDs may cause serious environmental damage. Indeed, the concentration of NSAIDs in the environment increases dramatically from about 10^–11^ mol/L in natural water^3^ to 10^–6^ mol/L in wastewater.^4^ Hence, there is a need for very sensitive methods to monitor and detect these NSAIDs in a natural environment. In pharmaceutical compositions, the NSAID concentrations are around 4 orders of magnitude higher,^2^ but the selectivity issues become important to distinguish adulteration and to discriminate, for example, counterfeit drugs.^5^ Therefore, the detection of anti-inflammatory drugs and their careful concentration screening in pharmaceutical compositions are important and challenging analytical tasks.

The most commonly used methods for NSAID analysis are instrumental methods, such as high-performance liquid chromatography (HPLC), gas chromatography with different detection end-units, such as GC-MS for instance,^6^ spectrophotometry,^7^ and others. They have been able to determine NSAID concentrations with high precision and low detection limits; however, costly equipment, complex operation, nonportability, the need for sample pretreatment, often including preconcentration, and the need for qualified personnel involvement make the application of these analytical methods cumbersome, especially for routine analysis and in-field measurements.

In recent years, chemical sensors have been actively employed for the determination of pharmaceuticals (especially nonsteroidal analgesics) due to their adequate selectivity and sensitivity in a wide linear dynamic range, simplicity of sample preparation and device use, low cost, and high portability.^8^ Examples of potentiometric^9^ and impedimetric^10^ sensors, conducting molecularly imprinted polymers coupled with electrochemistry,^11^ and multisensory systems based on different transduction principles^5,12^ have been reported in the literature.

Among the above-listed classes of sensors, ion-selective electrodes (ISEs) are of particular interest. The theoretical basis of functioning of these devices is well-established, and sensors with improved characteristics and appropriate selectivity can be easily developed by precisely tuning the composition of the ISE sensing membrane. However, in most of the previously reported works, only ion-exchangers^13^ and/or ion-exchanger/analyte ion pairs^13a,14^ have been used inside ISE membranes for NSAID assessment. To a much lesser extent, the application of macrocyclic compounds such as calixarenes,^15^ porphyrins,^16^ or cyclodextrins^17^ as ionophores for the selective detection of nonsteroidal painkillers has been reported. A different example was reported by Nazarov et al., who tuned the composition of ISE membranes for ibuprofen detection based on *N*-trifluoroacetylbenzoic acid heptyl ester as a neutral carrier, sensitive to anions classified as hard Lewis bases.^18^

Since NS and KF are mainly used as salts (i.e., in their carboxylate form) in pharmaceutical compositions, H-bond-based ionophores could be exploited in the development of ISE membranes for their selective and sensitive detection. During the last few decades, the squaramide scaffold, together with ureas, thioureas, selenoureas, amides, sulfonamides, and selenamides, has become quite popular in the design of anion receptors.^19^ Indeed, squaramides possess peculiar features, such as the aromaticity of the cyclobutadiene ring and the directionality of NHs, which make them ideal candidates for the design of H-bond-based receptors for anion binding, mostly halides.^20^ Interestingly, squaramides can also act as potent ionophores for the transport of chloride anions across lipid membranes due to a delicate balance between their lipophilicity and anion affinity.^21^ However, when properly functionalized, squaramides were found to effectively recognize also oxyanions such as SO_4_^2– ^^22^ and H_2_PO_4_^–^^23^ and carboxylate anions such as AcO^–^ and Benz.^24^ Moreover, the presence of the indole group as a substituent in the structure of acyclic squaramides was demonstrated to cause an enhancement of the anion recognition properties due to the cooperativity of the H-bond donor sites in stabilizing the formation of the host–guest adducts.^25^ Based on these considerations, herein, we investigate a novel family of acyclic squaramide-based receptors **L1**–**L5** tested as ionophores for the development of potentiometric sensors for NSAIDs detection in aqueous media through hydrogen-bond formation (Scheme 2). We demonstrate here that the selective potentiometric sensing of NSAIDs can be achieved by incorporating acyclic indole-substituted squaramide-based ionophores inside solvent PVC-based polymeric membranes with a careful variation of the ionophore/anion-exchanger ratio. It is interesting to note that, to the best of our knowledge, this represents one rare example of the use of squaramides for the development of potentiometric ISE.^26^ Among the tested ionophores, **L1**, **L4**, and **L5** receptors have shown an anti-Hofmeister selectivity with the highest affinity toward KF^–^ and NS^–^. The developed sensors were applied for KF^–^ detection in pharmaceutical compositions with the relative analytical error, R%, lower than 1% and recoveries in the range of 95.1–111.8%. Finally, the application of the chemometric approach allowed to effectively discriminate between KF^–^ and NS^–^, which are structurally very similar.

**Scheme 2:**
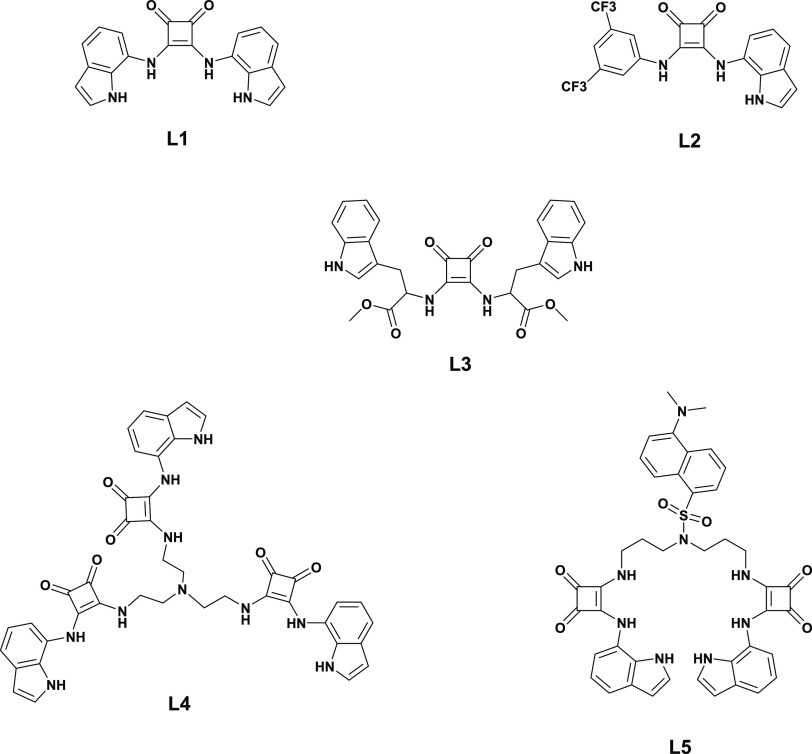
Chemical Structures of Acyclic Squaramide Receptors L1–L5

## Experimental Section

### Materials and Methods

High-molecular-weight poly(vinyl chloride) (PVC), tris(2-ethylhexyl) phosphate (TOP), tridodecylmethyammonium chloride (TDMACl), anhydrous tetrahydrofuran (THF), NaNO_2_, NaCl, NaBr, NaNO_3_, CH_3_CO_2_Na, NaClO_4_, NaSCN, NaHCO_3_, Na_2_SO_4_, sodium benzoate (NaBenz), ibuprofen sodium salt (NaIB), ketoprofen sodium (NaKF) and ketoprofen lysine (Lys-KF) salts, and naproxen sodium salt (NaNS) were purchased from Sigma-Aldrich. Ultrapure water was used for aqueous solution preparation. All of the other chemicals were of analytical grade and used without further purification.

All reactions were performed in oven-dried glassware under a slight positive pressure of nitrogen. ^1^H NMR (600 and 300 MHz) and ^13^C NMR (151 and 75 MHz) spectra were determined on a 600 MHz Bruker and on a 300 MHz Bruker. Chemical shifts for ^1^H NMR were reported in parts per million (ppm), calibrated to the residual solvent peak set, with coupling constants reported in Hertz (Hz). The following abbreviations were used for spin multiplicity: s, singlet; d, doublet; t, triplet; q, quadruplet; m, multiplet. Chemical shifts for ^13^C NMR spectra were reported in ppm, relative to the central line of a septet at δ = 39.52 ppm for DMSO-*d*_6_. All solvents and starting materials were purchased from commercial sources when available (Merck Europe, Fluorochem U.K.).

### Syntheses of **L1–L5**

**Scheme 3:**
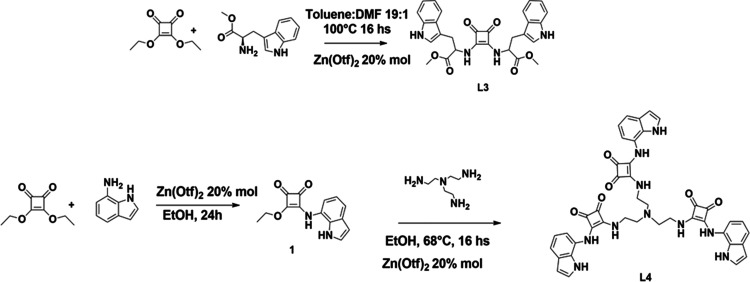
Synthetic Pathway for L3–L4

**L1** and **L2** were synthesized as previously reported.^25,27^ The synthesis of **L5** has also been recently described.^28^ Receptors **L3** and **L4** were prepared by modification of the procedure reported in the literature and described in Scheme 3. Compounds **L3** and **L4** were obtained in satisfactory to good yields (31 and 98%, respectively) and fully characterized (see the Supplementary Information, SI, Figures S1–S4 for ^1^H- and ^13^C NMR spectra of the intermediates **1**, **L3**, and **L4**).

#### Synthesis of 3-Indol-4-diethoxycyclobut-3-ene-1,2-dione (**1**)

The procedure found in the literature was modified to prepare this compound.^29^ To a stirred solution of 3,4-diethoxycyclobut-3-ene-1,2-dione (200 mg, 1.18 mmol) and zinc trifluoromethanesulfonate [Zn(OTf)_2_] (10 mol %) in dry ethanol (10 mL), 7-aminoindole (140 mg, 1.06 mmol) was added at room temperature. The reaction progress was monitored by TLC chromatography (SiO_2_, n-hexane/ethyl acetate 1:1 v/v). Once completed, the solvent was removed under reduced pressure and the crude product was purified by column chromatography (SiO_2_, hexane/ethyl acetate 3:2 v/v). The fractions containing the desired product were combined, and the solvent was evaporated, collecting it as a crude brown solid (227 mg, 0.9 mmol, 85% yield). ^1^H NMR (600 MHz, DMSO-*d*_6_, 298 K) δ_H_ (ppm): 11.07 (s, 1H), 10.57 (s, 1H), 7.47 (d, *J* = 7.7 Hz, 1H), 7.44 (t, *J* = 2.3 Hz, 1H), 7.12 (s, 1H), 7.05 (t, *J* = 7.7 Hz, 1H), 6.55 (t, *J* = 2.1 Hz, 1H), 4.74 (q, *J* = 7.1 Hz, 2H), 1.39 (t, *J* = 7.3 Hz, 3H) ^13^C NMR (151 MHz, DMSO-*d*_6_, 298 K) δ_C_ (ppm): 184.6, 178.7, 171.0, 129.8, 129.4, 126.2, 122.7, 119.4, 118.2, 114.9, 102.3, 69.7, 16.0. Elemental analysis (%) calcd. for C_14_H_12_N_2_O_3_ (% found): C: 65.62 (65.21), H: 4.72 (4.79), N: 10.93 (10.87).

##### Synthesis of (2S,2′R)-Dimethyl 2,2′-((3,4-Dioxocyclobut-1-ene-1,2-diyl)bis(azanediyl))bis(3-(3a,7a-dihydro-1H-indol-3-yl)propanoate) (**L3**)

To a stirred solution of 3,4-diethoxycyclobut-3-ene-1,2-dione (254 mg, 1.49 mmol) and [Zn(OTf)_2_] (20 mol %) in toluene/DMF (19:1 v/v, 6 mL), L-tryptophane methyl ester (706 mg, 3.23 mmol) was added. The solution was heated at 100 °C and stirred for 24 h. When the solution was cooled to room temperature, a precipitate was observed and isolated by filtration. The solid was further washed with methanol (3 × 5 mL) and dried under reduced pressure to remove the residual methanol. The residual was dissolved in ethyl acetate (2 mL) and precipitated with n-hexane. The solid was filtered, and the product was purified by flash chromatography (from n-hexane/ethyl acetate 3:2 v/v to n-hexane/ethyl acetate 1:2 v/v), obtaining the product as a white solid (0.2347 g, 0.4561 mmol, 31% yield) mp = 220–222 °C; ^1^H NMR (300 MHz, DMSO-*d*_6_, 298 K): δ_H_ 10.92 (s, 2H, NH), 7.97 (d, 2H, NH, *J* = 6 MHz), 7.44 (d, 2H, *J* = 6 MHz, ArH), 7.33 (d, 2H, *J* = 6 MHz, ArH), 7.05 (m, 4H, ArH), 6.95 (t, 2H, *J* = 6 MHz, ArH), 5.05 (q, 2H), 3.66 (s, 6H), 3.23 (d, 4H, *J* = 6 MHz) ^13^C (125 MHz, DMSO-*d*_6_, 298 K): δ_C_ 183.2, 171.9, 167.5, 136.5, 127.7, 124.4, 121.5, 119.0, 118.6, 111.9, 108.5. Elemental analysis (%) calcd. for C_28_H_26_N_4_O_6_ (% found): C: 65.36 (65.41), H: 5.09 (5.11), N: 10.89 (10.87). E-MS(+): *m*/*z* = 515, calcd. 514 for [M – H]^+^.

##### Synthesis of 4,4′,4″-((Nitrilotris(ethane-2,1-diyl))tris(azanediyl))tris(3-((1H-indol-7-yl)amino)cyclobut-3-ene-1,2-dione) (**L4)**

A stirred solution of compound **1** (200 mg, 0.78 mmol) and [Zn(OTf)_2_] (20 mol %) in dry EtOH (20 mL) was warmed at 68 °C. Then, a solution of tris(2-aminoethyl)amine (TREN) (22.8 mg, 0.16 mmol) in dry EtOH (1 mL) was added dropwise. The formation of a pale-yellow precipitate was observed. The solid was then filtered off and washed with ethyl acetate (3 × 5 mL) to remove the residual unreacted mono-squaramide. The solid was dried under vacuum, and the product was collected as a pale-brown solid (122 mg, 0.15 mmol, 98% yield). mp > 300 °C; ^1^H NMR (300 MHz, DMSO-*d*_6_, 298 K): δ_H_ 10.81 (s, 3H, NH), 9.71 (s, 3H, NH), 7.38 (m, 6H, NH, ArH), 7.24 (s, 3H, ArH), 6.99 (m, 6H, ArH), 6.49 (q, 3H, ArH), 3.69 (s, 6H), 2.79 (s, 6H), ^13^C NMR (151 MHz, DMSO-*d*_6_, 298 K): δ_C_ 185.22, 181.71, 169.38, 165.33, 129.74, 126.26, 123.39, 119.80, 117.21, 113.99, 102.51, 42.30. Elemental analysis (%) calcd. for C_42_H_36_N_10_O_6_ (% found): C: 64.94 (64.91), H: 4.67 (4.65), N: 18.03 (17.8ì97). ESI-MS(+): *m*/*z* = 777, calcd. 776 for [M – H]^+^.

### NMR Binding Studies

^1^H NMR titrations of **L1–L5** were performed by adding aliquots of a putative anionic guest (KF^–^ and NS^–^ as their sodium salts, 0.075 mol/L) in a solution of the receptor (0.005 mol/L) in DMSO-*d*_6_/0.5% water and DMSO-*d*_6_/10% water. The chemical shift of the signals attributed to the hydrogen-bond donor sites of the receptors was followed during the titration. The titration curves were fitted by using a proper binding model by the open-source program BindFit^30^ to collect the association constant for the formation of the expected adduct.

### Electrode Construction and Polymeric Membrane Preparation

Polymeric membranes were prepared by incorporating 0.5 wt % **L1–L5** and 0.2–6 equiv of the TDMACl anion-exchanger inside a polymeric matrix containing PVC and a TOP plasticizer in a 1:2 ratio by weight. The tested membrane compositions are listed in Table 1.

**Table 1:** Composition of the Tested Solvent Polymeric Membranes Based on L1–L5

				slope, mV/dec	
membrane		ligand, 0.5 wt %	TDMACl, equiv	NaNS	Lys-KF
1	mb 1.1	**L1**	0.25	–76.5 ± 7.0	–54.5 ± 4.7
2	mb 1.2		0.50	–67.1 ± 1.6	–42.5 ± 4.8
3	mb 1.3		1.0	–63.8 ± 2.3	–43.5 ± 3.6
4	mb 1.4		2.0	–76.7 ± 6.8	–44.4 ± 2.3
5	mb 2.1	**L2**	0.25	–15.1 ± 2.2	–18.8 ± 2.1
6	mb 2.2		0.5	–10.0 ± 3.7	–17.7 ± 3.6
7	mb 2.3		1.0	–14.8 ± 3.5	–19.5 ± 0.7
8	mb 2.4		3.75	–74.3 ± 6.5	–57.5 ± 6.4
9	mb 3.1	**L3**	0.25	–16.1 ± 0.1	–6.7 ± 0.5
10	mb 3.2		0.5	–27.7 ± 1.5	–21.1 ± 4.0
11	mb 3.3		1.0	–26.1 ± 1.0	–22.7 ± 0.1
12	mb 3.4		4.0	–29.4 ± 0.5	–26.5 ± 1.2
13	mb 4.1	**L4**	0.3	–71.8 ± 3.6	–56.7 ± 5.1
14	mb 4.2		0.75	–72.8 ± 2.3	–56.7 ± 3.6
15	mb 4.3		1.0	–68.9 ± 6.1	–53.3 ± 5.1
16	mb 5.1	**L5**	0.2	–68.9 ± 1.2	–43.5 ± 5.0
17	mb 5.2		0.5	–72.2 ± 3.7	–44.1 ± 4.5
18	mb 5.3		1.0	–47.1 ± 1.7	–31.8 ± 3.4
19	mb 5.4		6.0	–71.1 ± 1.1	–62.5 ± 6.0
20	mb 6	-	10 wt %	–14.41 ± 5.0	–14.9 ± 4.8

The membranes (approximately 100 mg total weight) were dissolved in 1 mL of THF. 10 μL of each membrane composition was cast onto Pt disk electrodes of 2 mm diameter incorporated inside Teflon and soaked for at least 12 h in a 0.01 mol/L solution of NaCl and tested *vs* a saturated calomel electrode (SCE) reference in individual solutions of KF^–^, NS^–^, and interfering IB^–^, Benz^–^, ClO_4_^–^, SCN^–^, NO_3_^–^, NO_2_^–^, Cl^–^, Br^–^, AcO^–^, HCO_3_^–^, and SO_4_^2–^ anions in the 1.0 × 10^–7^–1.0 × 10^–4^ mol/L concentration range. The calibration solutions were prepared by consecutive additions of calculated amounts of corresponding 0.1 mmol/L and 0.01 mol/L stock solutions of different salts to 50 mL of distilled water used as a background solution. Each membrane was tested in parallel with two freshly prepared ion-selective electrodes (ISEs), and the measurements were repeated for three consecutive sessions (*n* = 6). Prior to measurements, electrode potentials were stabilized to constant potential values (it takes approximately 60–600 s). In order to plot together and compare ISE responses of the tested analytes in solution without background influence, the mathematical correction of the electrode baseline signal in a distilled water background was applied to obtain the same initial potential value.

### Potentiometric Measurements and Selectivity Coefficient Estimation

Before each measurement, the electrodes were rinsed with distilled water and carefully dried with filter paper; electrode responses were tested by successive additions of increasing amounts of the tested analyte to the background solution. For this, the potentials of the galvanic cell comprising the tested electrodes and an SCE reference were measured with primary ion solutions in the concentration range of 1.0 × 10^–7^–1.0 × 10^–4^ mol/L as well as in the interfering ion solutions in the same concentration range. During the measurements, the solutions were stirred with a magnetic stirrer; the pH of all tested solutions was controlled with an Orion 9165BNWP combination sure-flow pH glass electrode (Thermo Scientific). Between measurements, the electrodes were stored in a 0.01 mol/L solution of NaCl.

The effect of pH on the **L1–L5**-based membrane responses was evaluated, as previously described,^31^ by continuous readings of the membrane responses in a universal buffer solution (UBS, prepared with 6.7 mmol/L citric acid, 11.4 mmol/L boric acid, and 0.01 mol/L NaH_2_PO_4_, initial pH 2.8) upon the addition of equal small amounts (50 μL) of 1 mol/L NaOH to 50 mL of UBS up to the final pH of 10.14. A pH glass electrode was employed for pH readings during the measurements to control solution acidity.

The selectivity of the **L1**–**L5**-based membranes was estimated with the separate solution method (SSM) according to the methodological recommendations described in the literature.^32^ The selectivity coefficients were estimated for solutions of 10^–4^ mol/L concentration using the following equation
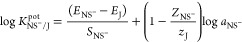
1where *E*_NS^–^_ is the potential of the electrode in the primary NS^–^ ion solution; *E*_J_ is the potential of the electrode in the interfering ion solution; *S*_NS^–^_ is the slope in the primary NS^–^ ion solution (the theoretical value of −59.2 mV/dec was used for calculus; the cases where the selectivity coefficient values were calculated in the absence of a close-to-Nernstian slope for the primary ion are specified separately; see Figure 4 for details); *Z*_NS^–^_ is the primary ion charge; and *z*_J_ is the interfering ion J charge. It should be noted that the presented estimated selectivity coefficient values may be dependent on experimental conditions.

Concentrations of HCO_3_^–^ and SO_4_^2–^ interfering ions were calculated according to their acidic dissociation constants and pH.^33^

The standard addition method was employed to estimate the KF^–^ amount in real pharmaceuticals, Okitask by Dompé in particular. For this, the content of an Okitask 40 mg pocket was weighed and then ground in a mortar. The amount of powder corresponding to the active substance according to its solubility in water (1.9 mg) was weighed and dissolved in 2.5 mL of freshly distilled water (pH 6.9) and sonicated for 10 min for dissolution. The sample solutions of concentration 4.75 × 10^–5^ mol/L were prepared by adding 1.25 mL of the stock solution into 50 mL of distilled water; the final solution pH of 4.15 was measured with a pH glass electrode. The potentials of selected membranes were measured versus the SCE reference before (E1) and after the two additions (E2) of 250 μL of 2.2 × 10^–2^ mol/L Lys-KF standard solution (variation of KF^–^ from 1.0 × 10^–4^ to 2.0 × 10^–4^ mol/L) to the sample solution. The concentration of KF^–^ in the tested sample was calculated as
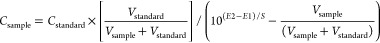
2where *S* is a potentiometric response slope estimated as the electrode potential difference *vs* the added analyte concentration variation upon two consecutive additions. The accuracy of KF^–^ assessment was estimated through the percentage of known initial concentration recovery, *R* %, and the relative error of analysis, RSD%.

### Multisensory Data Treatment

Multisensory data treatment was performed with a commercial Unscrambler (v 9.1, 2004, CAMO PROCESS AS, Oslo, Norway). Chemometric data analysis included identification, classification, and quantitative estimation of the NSAID concentration.^34^ The principal component analysis (PCA) technique was employed for identification. Partial least-square regression (PLS) was used to estimate KF^–^ and NS^–^ concentrations. The mean normalization procedure was used for raw data through data analysis. Due to the restricted number of measurements composing the data set, a leave-one-out validation was applied. The RMSEP (root-mean-square error of prediction) and correlation coefficients, *R*^2^, of predicted *vs* measured correlation lines were used to evaluate the efficiency of the constructed regression models.

## Results and Discussion

### Binding Properties of L1–L5 in Solution toward NaNS and NaKF

The binding properties of **L1**–**L5** toward NS^–^ and KF^–^ (as their sodium salts) were preliminarily studied by means of ^1^H NMR titrations in DMSO-*d*_6_/0.5% water and DMSO-*d*_6_/10% water by following the downfield shift of the signals attributed to the squaramide NHs and the indole NHs. The association constants and related errors (%) calculated using 1:1 and 1:2 binding models by the open-source program BindFit are summarized in Table 2 and Figures S5–S18.

**Table 2:** Association Constants (*K*_ass_/(mol/L)^−1^)^a^ for the Formation of the 1:1 (*K*_11_) and 1:2 (*K*_12_) Host–Guest Adducts of L1–L4, with the Sodium Salts of Naproxen (NaNS) and Ketoprofen (NaKF) in DMSO-*d*_6_/0.5% Water and DMSO-*d*_6_/10% Water at 298 K

	*K*_ass_ (mol/L)^−1^							
	1:1 stoichiometry							
	L1		L2		L3		L4	
anionic species	DMSO-*d*_6_/0.5% water	DMSO-*d*_6_/10%water	DMSO-*d*_6_/0.5% water	DMSO-*d*_6_/10%water	DMSO-*d*_6_/0.5% water	DMSO-*d*_6_/10%water	DMSO-*d*_6_/0.5% water	DMSO-*d*_6_/10%water
**NS**^**–**^	N.D.	2.38 × 10^4^	N.D.	N.D.	1.70 × 10^2^			
**KF**^**–**^	2.15 × 10^4^	2.10 × 10^3^	N.D.	N.D.	1.30 × 10^2^			
1:2 stoichiometry								
**NS**^**–**^							1.475 × 10^5^	5.00 × 10^2^
**KF**^**–**^							1.35 × 10^3^	2.00 × 10^2^

a *K*_11_ refers to the equilibrium *L* + *I*^–^ = *LI*^–^; *K*_12_ refers to the equilibrium *LI*^–^ + *I*^–^ = *LI*_2_^2–^; see also the SI.

**Figure 1:**
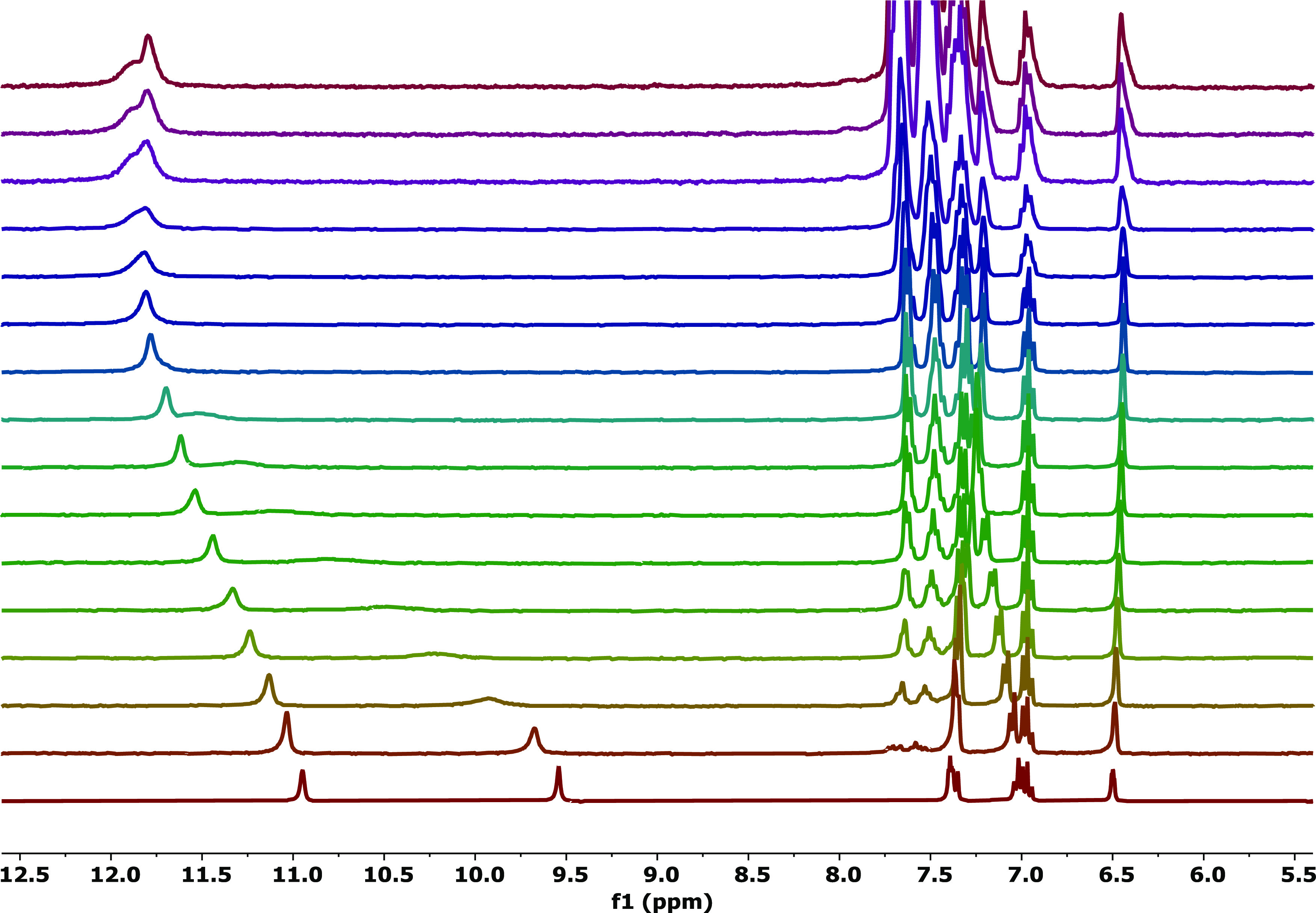
Stack plot of the ^1^H NMR titration of **L1** (5.0 × 10^–3^ mol/L) with NaKF (7.5 × 10^–2^ mol/L) in DMSO-*d*_6_/0.5% water.

In DMSO-*d*_6_/0.5% water, **L1** strongly interacts with both the anionic guests KF^–^ and NS^–^. Particularly, as reported in the stack plots for the titrations of **L1** in the presence of increasing amounts of NaKF and NaNS (Figures 1 and S9, respectively), the signal attributed to the squaramide NHs (at 9.55 ppm) was dramatically downfield-shifted (Δδ = 2.35 ppm and Δδ = 2.51 ppm for the addition of NaKF and NaNS, respectively), while the downfield shift of the signal attributed to the indole NHs at 10.95 ppm was less significant (Δδ = 0.85 ppm and Δδ = 0.95 ppm for the addition of NaKF and NaNS, respectively). This experimental evidence suggested a strong host–guest interaction between **L1** and the guest species. However, only in the case of the titration in the presence of NaKF the fitting of the data allowed to calculate the association constant for the formation of the 1:1 adduct, whereas the data for the titration with NaNS could not be fitted.

For this reason, we decided to repeat the titrations in a more competitive solvent mixture (DMSO-*d*_6_/10% water) to modulate the host–guest interaction. As expected, under these novel experimental conditions, we were able to calculate the association constants for the formation of the 1:1 adducts of **L1** with both NS^–^ and KF^–^. Indeed, an association constant of 1 order of magnitude higher for the formation of the 1:1 adduct with NS^–^ was estimated with respect to that with KF^–^ (see Table 2 and Figures S8 and S10 in the SI for the stack plot of titrations of **L1** with NaKF and NaNS in DMSO-*d*_6_/10% water, respectively).

In the case of **L2**, the signals attributed to the squaramide NHs (at 10.15 and 9.99 ppm) disappeared during the titrations conducted in DMSO-*d*_6_/0.5% water solution, whereas the downfield shift of the signal attributed to the indole NH at 11.04 ppm was observed (see the SI, Figures S11 and S12 for the stack plots of the titrations in the presence of NaKF and NaNS, respectively). This evidence suggested an interaction between **L2** and both NS^–^ and KF^–^, but the titration data did not allow calculation of the association constants. Indeed, also in this case, we decided to perform the titrations in a more competitive solvent mixture (DMSO-*d*_6_/10% water). Unfortunately, also in this case, the experimental data could not be fitted (see the SI Figures S13 and S14 for the stack plots of the titrations of **L2** in the presence of NaKF and NaNS, respectively).

**L3** showed a low affinity toward the guests, and no selectivity was observed even in the DMSO-*d*_6_/0.5% water solution. This behavior could be explained by considering a possible open conformation of the receptor in which the indole and the squaramide NHs point in different directions (as shown in Scheme 3), causing a scarce cooperation between the two hydrogen-bond donor sites in the anion binding. This hypothesis is confirmed by the fact that the signal attributed to the indole NHs (at 10.93 ppm) did not shift upon the addition of an increasing amount of anionic guests (see the SI, Figures S5 and S6 for the stack plots of the titrations of **L3** with NaKF and NaNS, respectively). It is interesting to note that, according to the values of the association constants in Table 2, **L4** strongly binds both the anionic guests with a host–guest 1:2 stoichiometry in DMSO-*d*_6_/10% water. This is probably due to the presence of nine hydrogen-bond donor sites and to the intrinsic flexibility of the TREN unit used as a spacer among the squaramide moieties. Indeed, upon the addition of increasing amounts of anionic species, the signals attributable to the squaramide NHs (at 9.69 ppm for the NH adjacent to the indolyl moiety and 7.22 ppm for the NH adjacent to the alkyl chain of the TREN moiety, respectively) and the signal corresponding to the indole NHs (at 10.87 ppm) undergo a dramatic downfield shift, which is more significant in the presence of NaNS (see Figures S15 and S16 for the ^1^H NMR titration in the presence of NaKF and NaNS, respectively).

The obtained ^1^H NMR titration curves were fitted with 1:1 and 1:2 binding models, and the results demonstrated a strong interaction with both the anionic guests only in a 1:2 stoichiometry. Encouraged by these results, we decided to conduct the same titrations in a more competitive solvent mixture (DMSO-*d*_6_/10% water; see Figures S17 and S18). As expected, under these novel experimental conditions, we were still able to calculate the association constants for the formation of the 1:2 adducts and to confirm the strong affinity of **L4** toward the anionic guests, even in this more competitive medium. In the case of **L5**, the results of the ^1^H NMR solution studies with both NaNS and NaKF have been reported elsewhere and demonstrate the formation of 1:2 adducts with a stronger affinity to NS^–^.^28^

### Potentiometric Properties of L1–L5-Based ISEs

The properties of 19 membranes (entries 1–19 in Table 1) of different compositions obtained by incorporation of 0.5 wt % **L1–L5** inside PVC/TOP polymeric matrices were investigated and compared with the response of only the TDMACl (10 wt %) anion-exchanger-based membrane (entry 20, Table 1). For each receptor, the amount of added anion-exchanger varied in a different range (from 0.2 to 6 eq) to stabilize membrane neutrality and to ensure the analyte anions’ membrane permselectivity. The sensitivity tests of **L1**–**L5**-based membranes were carried out across the KF^–^ and NS^–^ concentration range of 1.0 × 10^–7^–1.0 × 10^–4^ mol/L, chosen in accordance with the amounts of KF^–^ and NS^–^ ions in wastewater^3,4^ (about 10^–9^–10^–6^ mol/L) and in common pharmaceutical compositions^2,35^ (about 10^–4^ mol/L and higher). All of the tested ISEs demonstrated that the anionic sensitivity toward both analytes varied upon the variation of the receptor/anion-exchanger ratio; Table 1.

### Plasticizer Selection and pH Cross-Response

In previous works on KF^–^ and NS^–^ selective electrodes’ development reported by Lenik’s group, the best sensitivity parameters were reached for membranes based either on the methyltrioctylammonium chloride ion-exchanger (MTOA-Cl)^13d^ or on ion pairs, such as naproxen-tetraoctylammonium (TOA-NS), or tetraoctylammonium 6-methoxy-α-methyl-2-naphthaleneacetate,^14b^ plasticized with tris-butyl- or tris-octylphosphate plasticizers (TBP and TOP, respectively), having a similar medium-low polarity (relative dielectric constant, ε_TOP_ = 7.9). Moreover, it was demonstrated that the TBP plasticizer dissolves well the membrane active components, especially high-molecular-weight ionophores, for instance, cyclodextrins, and significantly increases the membrane conductivity.^17c^ We hence have selected the TOP plasticizer that nicely dissolves all of the membrane components and lowers the membrane resistance to investigate the binding affinity of acyclic squaramide receptors **L1**–**L5** toward KF^–^ and NS^–^ ions inside PVC-based polymeric membranes. The well-known cation-solvating properties of the TOP plasticizer, as well as its ability to compete with a primary ion in carrier binding,^36^ were also taken into account by keeping the amount of ionophores fixed and systematically varying the amount of the TDMACl ion-exchanger within the membrane phase. Furthermore, the effect of plasticizers with different functional groups and the correlation between the dielectric constant and lipophilicity of the plasticizer (and the membrane) should be considered for an accurate tuning of the selective properties of the acyclic squaramide ionophore-based membranes, and they will be the subject of a further investigation.

**Figure 2:**
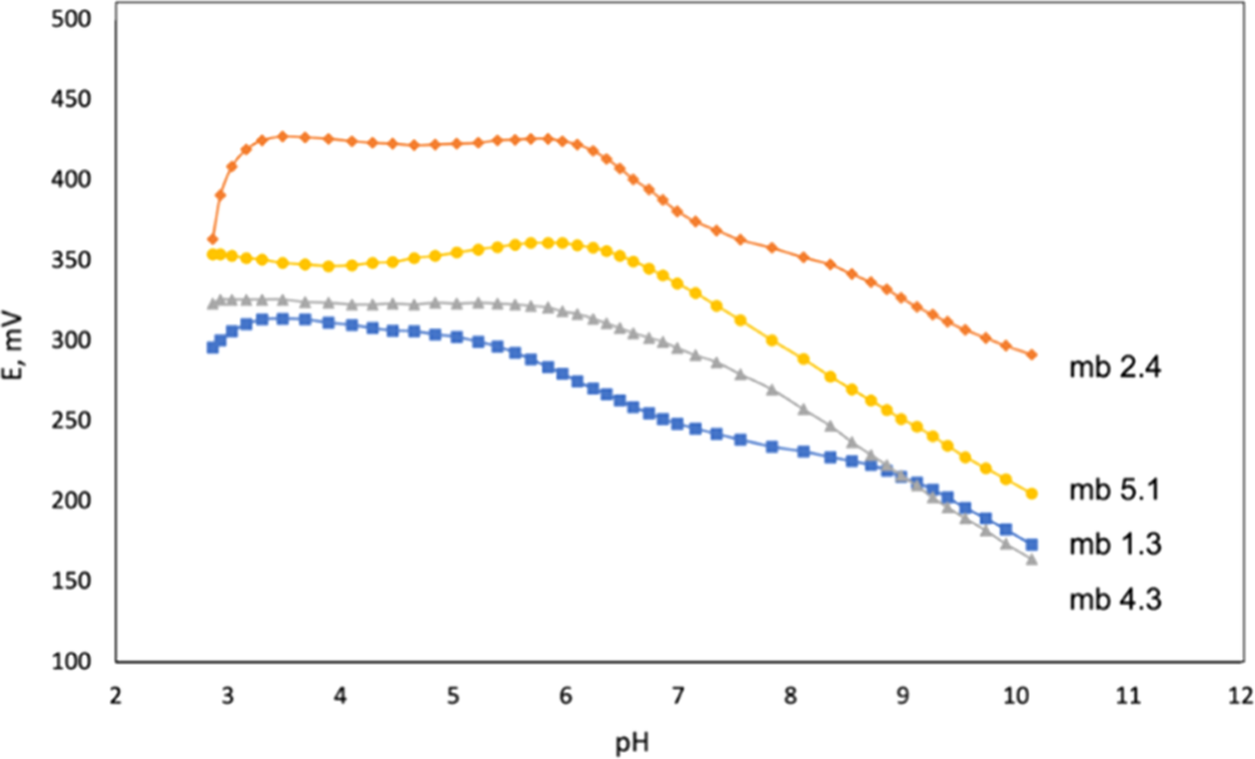
Effect of pH on the potential response of selected membranes.

The tests of the influence of pH on the potentiometric responses of **L1**–**L5**-based membranes toward KF^–^ and NS^–^ ions were performed in the pH range of 2.8–10.14 upon the addition of 1 mol/L NaOH solution into UBS. The results for the selected membranes based on receptors **L1**, **L2**, **L4**, and **L5** are illustrated in Figure 2. No pH side-effect on the response of the membranes based on **L4** and **L5** in the pH range 2.9–6.4 was recorded, while a narrower pH stability range from 3.2 to 5.2 and from 3.2 to 6.0 units was found for membranes mb 1.3 and mb 2.4, respectively, based on smaller-sized (and less −NH groups bearing) ligands **L1** and **L2**. To prove the stability and effective functionality of the developed membranes in the pH range 3.0–6.0, all further potentiometric evaluations were performed on a distilled water background.

During the calibrations, the pH of individual aqueous solutions of the tested anions was monitored with a pH glass electrode; the pH change of KF^–^ and NS^–^ solutions did not exceed 0.5 units upon 3 orders of magnitude of concentration variation (from 1.0 × 10^–7^ to 1.0 × 10^–4^ mol/L).

### Sensitivity of **L1**–**L5**-Based ISEs toward KF^–^ and NS^–^

**Figure 3:**
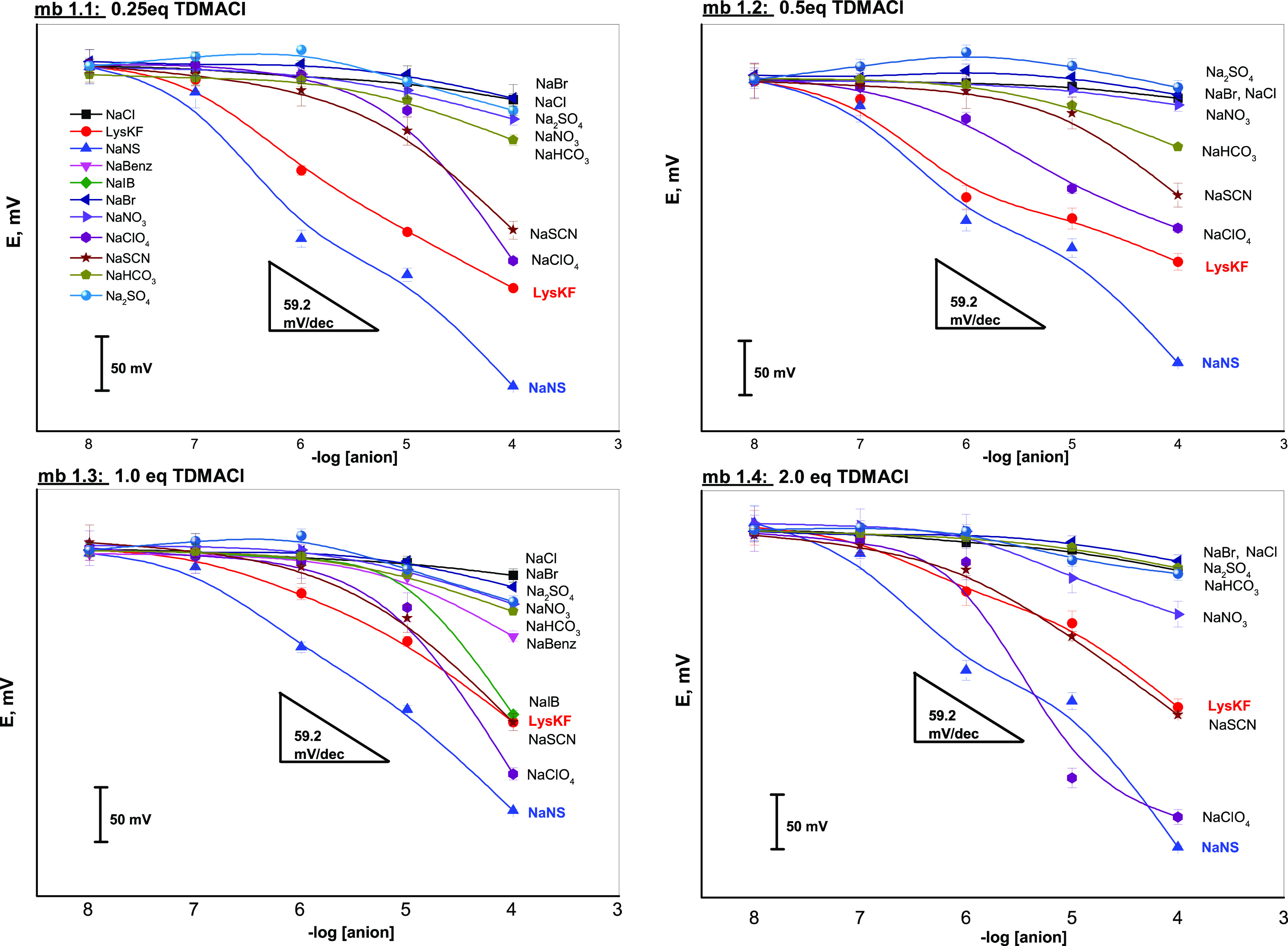
Potentiometric calibration curves of membranes mb 1.1–mb 1.4 doped with **L1** and 0.25, 0.5, 1.0, and 2.0 equiv of TDMACl, respectively, in individual solutions of KF^–^, NS^–^, and various interfering ions in the concentration range of 1.0 × 10^–7^–1.0 × 10^–4^ mol/L. Plots show error bars of four individual measurements (*n* = 4).

The potentiometric response curves of membranes mb 1.1–1.4, based on **L1** and containing correspondingly 0.25, 0.5, 1.0, and 2.0 equiv of TDMA^+^ lipophilic sites in individual solutions of the tested anions, are shown in Figure 3.

The enhanced sensitivity toward KF^–^ and NS^–^ with respect to other interfering anions can be observed over the tested concentration range. Furthermore, in the case of membranes mb 1.1 and mb 1.2, a higher sensitivity of only NS^–^ over KF^–^ was observed, and a linear response of mb.1.1 with a close-to-Nernstian slope of −54.5 mV/dec toward KF^–^ was recorded. The increase of the TDMA^+^/**L1** ratio from 0.25 to 0.5, and then to 1.0 and 2.0, resulted in a slight decrease of KF^–^ sensitivity with a decrease of the slope to a sub-Nernstian −44.4 mV/dec value for membrane mb 1.4 and an increase of the interfering influence of highly lipophilic ClO_4_^–^ and SCN^–^ ions. The highest sensitivity toward NS^–^ of membrane mb 1.3, containing 1 equiv of TDMACl with a slope of −63.8 mV/dec close to the theoretical Nernstian value, well corresponds to the higher formation constant of the 1:1 adduct between **L1** and this anion (see above), probably due to the cooperativity between the indole and the squaramide −NH groups in stabilizing the anion adducts. Indeed, as was previously demonstrated for the binding properties of **L1** toward chloride species,^25,37^ the lone pairs of the chloride ion interact with both types of H-bond donor groups of the receptor, resulting in a 1:1 host–guest adduct formation (see the SI, section 3.1, for a detailed discussion). Even a 10-fold higher association constant value was reported for acetate anion binding (*K*_ass_ > 10^4^ and 1199 mol^–1^/L^–1^ for AcO^–^ and Cl^–^, respectively, in DMSO-*d*_6_/0.5%).^25^ A similar binding mechanism might be expected for KF^–^ and NS^–^ anions, which should occur *via* the interaction of −NH groups of the receptor with the lone pairs of the two oxygen atoms of the carboxylate group (−COO^–^) in KF^–^ and NS^–^.

The analysis of both potentiometric calibration curves for membranes doped with **L1** (Figure 3) and **L2**–**L5** (Figures S19–S22), and slopes (Table 1), highlighted the influence of the chemical structure of **L1**–**L5**, the effective number of hydrogen-bond donor groups in the ionophores, and the ionophore/TDMACl molar ratio on the sensitivity of developed ISEs toward NSAIDs.

Despite the simple and symmetric structure of **L1**, and its binding properties toward KF^–^ and NS^–^, which have been studied in detail by ^1^H NMR titrations in DMSO-*d*_6_/water (see above), the interpretation of the potentiometric sensitivity of **L1**-based membranes is quite ambiguous. The super-Nernstian slopes of **L1**-based membranes (see Table 1) suggest an interaction mechanism for the ionophore/anion mixture involving multiple binding/dissociation and/or ion-exchange processes, which may occur simultaneously or in sequence. The well-known ability of squaramide receptors to self-assemble by forming head-to-tail H-bonds in different solvent systems has been investigated and experimentally confirmed by crystallographic and differential scanning calorimetry (DSC) studies for various squaramide-based receptors.^19b,37,38^ Therefore, the super-Nernstian sensitivities of membranes mb 1.1–1.4 doped with **L1** might be ascribed to this ability and to the formation of **L1** aggregates (see Figure S23) in the membranes able to interact with the guests (see the SI for a more detailed description).

The replacement of one of the indole substituents in **L1** with the 3,5-bis(trifluoromethyl)phenyl moiety to give the nonsymmetric squaramide **L2** allowed to consider the effect of the presence of an electron-withdrawing group (EWG) on the lipophilicity of the receptor. Moreover, the effect of changing the position of the indole NH with respect to the cyclobutadiene ring by using the tryptophan methyl ester moiety for the symmetric squaramide **L3** was also taken into account. On the other hand, the effect of increasing the number of H-bond donor groups was evaluated in the case of the flexible TREN derivative **L4** and in the dansyl derivative **L5**.

In comparison to the **L1**-based membranes, the lack of one indole NH group in the structure of **L2** along with the introduction of the EWG resulted in a significant lowering of both KF^–^ and NS^–^ sensitivity for membranes mb 2.1–2.3 (doped with 0.25, 0.5, and 1 equiv of TDMACl, respectively, Table 1), with slightly higher (although sub-Nernstian) slopes registered for KF^–^ solutions (Figure S19). The membrane mb 2.4, with a higher amount of anion-exchanger (3.75 equiv with respect to the **L2** ionophore), showed higher potentiometric responses with a super-Nernstian slope of −74.3 ± 6.8 mV/dec for NS^–^ and −57.5 ± 6.4 mv/dec for KF^–^ and exhibited anti-Hofmeister selectivity (see the next sections for more details). Among all prepared membranes, only **L2**-based membranes with a low anion-exchanger/ionophore ratio (namely, mb 2.1–mb.2.3) exhibited a higher sensitivity in terms of higher slopes (although sub-Nerstian) toward the smaller sized and nonlinear KF^–^ anion with respect to NS^–^ ions. This higher sensitivity can be tentatively ascribed to the nonsymmetric **L2** squaramide structure allowing easier approaching and better fitting of nonlinear KF^–^ ions inside the ionophore cavity, as well as to the presence of the EWGs, which might additionally stabilize the two aromatic phenyl rings of the KF^–^ anion.

For the receptor **L3**, which adopts a conformation featuring spatially separated and oppositely directed indole and squaramide NH groups, a lower potentiometric response was observed toward both target anions, and in particular for the smaller-sized KF^–^ ion, probably due to the less effective receptor-anion binding discussed above (see Figure S20). This behavior is only barely affected by the amount of anion-exchanger used. The low affinity toward the guests and the absence of selectivity are in agreement with the low association constant values (*K*_ass_/(mol/L)^−1^) reported in Table 2.

The incorporation of **L4** in PVC-based membranes resulted in very efficient anion binding, in agreement with the results obtained from the binding studies performed by ^1^H NMR titrations with NaKF and NaNS in DMSO-*d*_6_/0.5% water. As can be noticed from Figure S21, the almost univariate response slopes toward KF^–^ (−55.6 ± 2.0 mV/dec) and NS^–^ (−71.2 ± 2.0 mV/dec) upon increasing the amount of anion exchanger from 0.3 to 0.75 and 1.0 equiv inside the membrane suggested that all of the binding NH sites in **L4** were initially occupied by chloride anions (most probably from the 0.01 mol/L NaCl solution used for membrane conditioning), and no ligand dimerization and **L4**–**L4** species formation occur in the membrane phase. This assumption is also supported by previously reported data on carboxylates binding on secondary squaramides in polar media.^24b^ Upon membrane calibration, the more lipophilic target KF^–^ or NS^–^ ions from solution could, in principle, substitute Cl^–^ ions from the corresponding ionophore/anion adducts inside the membrane and then form 1:1 and 1:2 adducts with the ionophore. The super-Nernstian membrane response could indicate the formation of host–guest adducts with different stoichiometries (i.e., 1:1, 1:2, etc.) inside the membrane. As stated above, the cooperation between different H-bond donor groups in **L4** might be the cause for the dramatic improvement of the anion recognition properties.

In comparison to **L1**, the introduction of two supplementary H-bond donor groups in the structure of **L5** resulted in a better sensitivity to KF^–^ for membrane mb 5.2 (44.1 ± 4.5 mv/dec) containing 50 mol % cationic sites with respect to the ionophore (see Figure S22). Interestingly, while **L1** forms with KF^–^ only the 1:1 adduct in the DMSO/H_2_O mixture (see Table 2), **L5** forms the 1:2 adduct in the same experimental conditions.^28^ Similar to membranes based on **L4**, the super-Nernstian slopes observed for the bigger-sized NS^–^ anion indicate the formation of a mixture of 1:1 and 1:2 adducts inside the membrane phase. The more rigid structure of **L5** and the longer aliphatic spacers result in the decrease of potentiometric response slopes for both KF^–^ or NS^–^ ions in comparison to **L1**- and **L4**-based membranes prepared with the same (or close) ionophore/TDMACL anion-exchanger ratio; see Table 1. Moreover, the low solubility of **L5** inside the solvent polymeric membrane phase did not permit fully characterizing the **L5** receptor performance as a selective ionophore for NSAID anions.

### Selectivity Evaluations

The incorporation of cationic lipophilic sites inside the anion-selective membranes is required both to promote the flux of analyte ions inside the membrane phase and to ensure membrane electroneutrality. Moreover, the amount of anion-exchanger introduced inside the membrane in different ratios with respect to the receptor concentration may elucidate the stoichiometry of the receptor-analyte adducts formed through an application of the phase-boundary model for selectivity prediction.^39^ According to this model, membrane potentiometric selectivity is controlled by the stability and stoichiometry of the adducts between the ionophore, **L**, and the target and interfering anions. However, systems in which the target and interfering ions each form adducts of only one stoichiometry have been mainly considered. Situations in which the ionophore may bind more than one target anion, or in which adducts of different stoichiometries are concurrently present in a membrane phase, have been less exploited.^40^ In fact, by varying the ratio of ionophore to ionic sites inside the membrane, the membrane selectivity may be interpreted by the presence of ionophore/target anion adducts of only one or different stoichiometries.

The following system of equations was simultaneously solved^40a^ to describe the mass balance inside the polymeric membrane in the case of competitive 1:1 and 1:2 ionophore/monovalent primary anion (I^–^) adduct formation
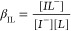

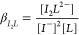

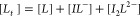
3
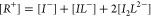

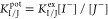

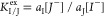
where β_IL_ and β_*I*_2_L_ are the binding constants of the primary anion I^–^ and the receptor **L** for formation of adducts of a 1:1 or 1:2 stoichiometry (see the SI), and *J*^–^ is a noncoordinating anion. *L*_t_ is the total ionophore concentration in the membrane, which is determined by the sum of the concentrations of the uncomplexed and complexed ionophore with the anion I^–^; R^+^ is the concentration of cationic lipophilic sites in a membrane that will keep the membrane electroneutrality for measurements with samples containing only ion I^–^. *K*_*I*/J_^pot^ is a potentiometric selectivity coefficient, determined according to the SSM method, and [I^–^] and [J^–^] are the concentrations of these ions in the sensing membrane when the membranes are exposed only to ions I^–^ or ions J^–^, respectively.

*K*_I/J_^ex^ is the single ion-exchange constant for the exchange of the primary ion I^–^ with the interfering J^–^ ion between an aqueous sample phase and the ionophore-free ISE membrane phase. If the membrane is exposed to solutions containing only J^–^ (for instance, during conditioning in NaCl, and *J*^–^ = Cl^–^), the J^–^ enters the membrane but does not form adducts with *L*, and hence, [*J*^–^] = [*R*^+^].

**Figure 4:**
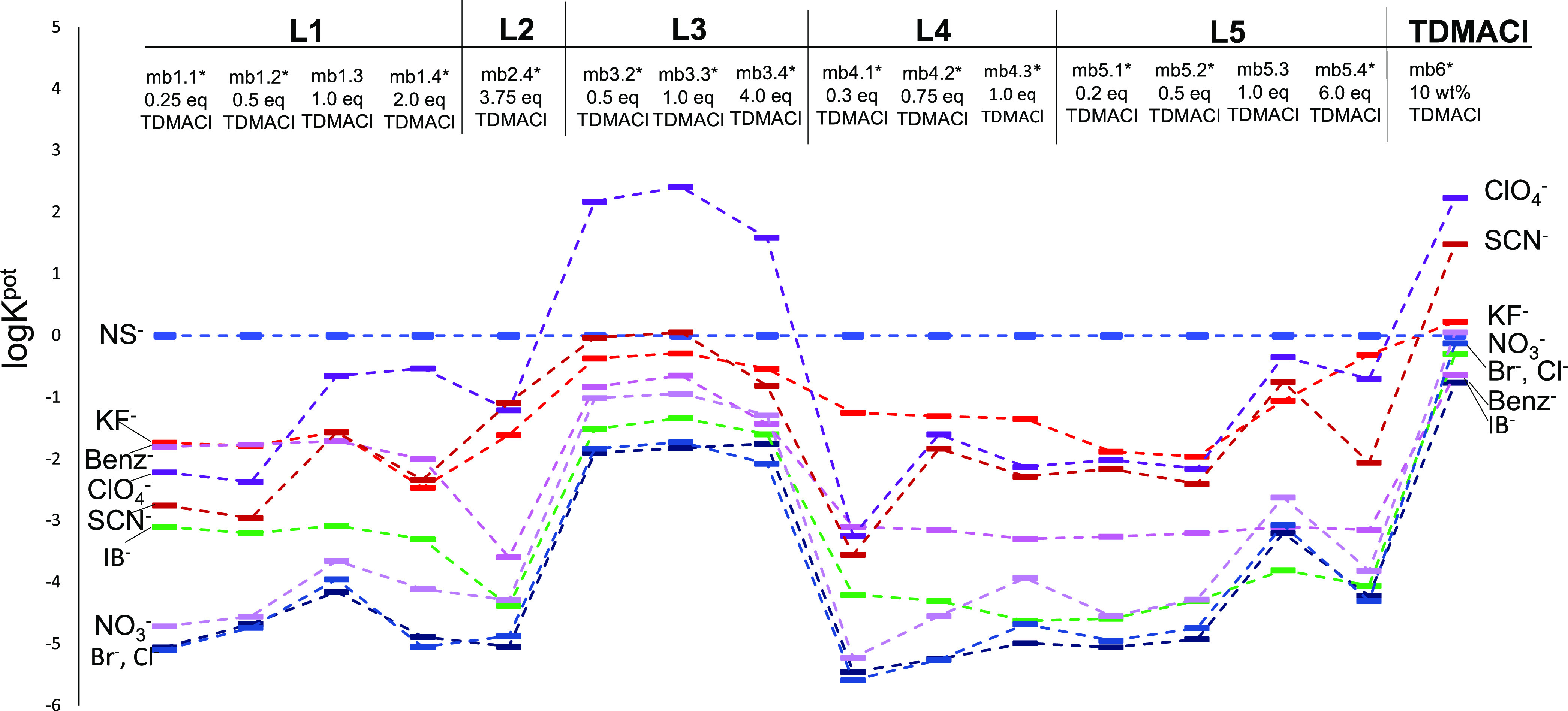
Potentiometric selectivity coefficients of membranes based on **L1**–**L5** ligands with varied amounts of the TDMACl anion exchanger (indicated in equiv relative to ligand content) for NS^–^ as the primary ion. The values of log *K*_I/J_^pot^ were estimated with the SSM method, and the slope of −59.2 mV/dec was used for calculus. For comparison, the selectivity of mb 6, formulated only with 10 wt % TDMACl, is also shown. The membranes not exhibiting Nernstian response are indicated with the (*) mark.

The values of *K*_I/J_^pot^ were predicted by solving the system of eq 3, where *L*_t_ is experimentally determined, and also may be expressed through different complexed forms calculated from the experimentally obtained binding constants *K*_11_ and *K*_12_ (see the SI for the details on equations solved for selectivity parameters), and plotted as a function of the lipophilic additive-to-ionophore ratio, [*R*^+^]/[*L*_t_]; Figures S24 and S25. The ionophore-primary ion binding mechanism may be elucidated by comparison of the obtained parametric plots in coordinates log *K*_I/J_^pot^*vs* [*R*^+^]/[*L*_t_] as reported in Figure 4, where the experimental potentiometric selectivity coefficients of the tested membranes based on **L1**–**L5** receptors for NS^–^ as the primary ion, and various interfering ions determined with the SSM method, are shown. The numerical values of log *K*_I/J_^pot^ are listed in Table S2. Additionally, the log *K*_I/J_^pot^ values for **L1**–**L5** based for KF^–^ as the primary ion are reported in Figure S26 and are listed in Table S3.

In the present work, we have considered two representative cases for membranes based on **L1** and **L4** receptors with the following experimental parameters: [**L1**_t_] = 14.6 mM, [**L4**_t_] = 6.4 mM, with β_IL_ for **L1** and β_I_2_L_ for **L4** equal to those experimentally evaluated by ^1^H NMR titration in DMSO-*d*_6_/10% water at 298 K (Table 2). The obtained parametric plots are shown in Figures S24 and S25, respectively. From Figure S24, it may be seen that for the receptor **L1**, for which the 1:1 ionophore/monovalent primary anion (I^–^) complexes are prevalently formed (*K*_11_ is 2.38 × 10^4^ mol^–1^/L^–1^ and *K*_12_ was settled to be 2 orders of magnitude smaller, 2.50 × 10^2^ (mol/L)^−1^; see SI), the selectivity for the primary ion decreases as the amount of lipophilic cationic sites in the membrane increases with respect to the ionophore.

On the contrary, for the **L4**-based membrane, where *K*_12_ is 1.475 × 10^5^ (mol/L)^−1^ as reported in Table 2, and *K*_11_ was set to be 2 orders of magnitude smaller, 1.55 × 10^3^ mol^–1^/L^–1^, the selectivity first remains unchanged and then slightly decreases with growth of the R^+^ amount in the membrane phase, while the estimated concentration of [I^–^] in the membrane remains almost unchanged due to 1:2 ionophore/I^–^ complex formation.

The obtained theoretical simulations are in accordance with the experimental results shown in Figure 4. As may be noticed from Figure 4, all of the tested acyclic squaramide receptors **L1**–**L5** have shown an anti-Hofmeister selectivity with high affinity toward KF^–^ and NS^–^ anions. Among all of the tested membranes, mb 1.1, mb 2.4, mb 4.1, and mb 5.1 showed a very low influence of most tested anions on NS^–^ selective response (except for perchlorate and benzoate anions, which significantly influenced the **L1**-based membrane response to KF^–^). For **L1**-based membranes, the increase of the ligand/anion-exchanger ratio from 0.25 to 2 equiv resulted in a slight decrease of selectivity for the membrane mb 1.3 doped with 1 equiv of TDMACl; at the same time, this membrane exhibited the best sensitivity with a close-to-Nernstian response of −57.0 mV/dec to the NS^–^ anion in a concentration range from 10^–6^ to 10^–4^ mol/L, confirming the 1:1 analyte/ionophore adduct formation. On the contrary, the significant increase in selectivity toward both KF^–^ and NS^–^ anions upon the lowering of the TDMACl amount to 0.3 and 0.5 equiv for **L4** and **L5** doped membranes, respectively, indicates the concurrent formation of primary ion complexes with mixed stoichiometries (1:1, 1:2).

The prevalent 1:2 analyte/ionophore binding for **L4** and **L5** is also confirmed by ^1^H NMR binding studies in DMSO-*d*_6_/0.5% water and DMSO-*d*_6_/10% water solutions. Unfortunately, the low solubility of **L5** inside the solvent polymeric membrane phase did not permit full characterization of the **L5** performance as a selective ionophore for NSAID anions. The **L3**-based membranes mb 3.1–3.3, along with the lowest selectivity among all of the tested membrane compositions, demonstrated a particularly high influence of perchlorate ions onto NS^–^ response, which represents a serious drawback of **L3** to be used as a selective receptor for NSAID detection.

### Real Sample Analysis and Multisensory Application

Since better discrimination among the two target ions NS^–^ and KF^–^ was observed for mb 1.3 and mb 4.3, these membranes, as well as mb 2.4 and mb 5.1, were employed for the assessment of KF^–^ ions in real pharmaceuticals, in particular, Okitask by Dompé. The results are reported in Table 3. The possibility of determining a known spiked amount of KF^–^ ions with a relative error, R%, around 1% was demonstrated. Moreover, the direct estimation of ketoprofen lysine salt in Okitask formulation with the developed ISEs with recoveries in the range of 95.1–111.8% indicates the suitability of acyclic squaramide ionophores for potentiometric NSAID sensing.

**Table 3:** Results of Lys-KF Determination in Okitask Formulation Using the Developed Arylic Squaramide-Based Sensors

		found			
added		mb 1.3	mb 2.4	mb 4.3	mb 5.1
[KF^–^]_spiked_, mol/L	1.0 × 10^–4^	1.11 × 10^–4^	9.5 × 10^–5^	9.3 × 10^–5^	1.1 × 10^–4^
RSD^a^,%		1.39	1.06	0.99	1.37
[KF^–^]_sample_^b^, mol/L	4.75 × 10^–5^	4.52 × 10^–5^	4. 61 × 10^–5^	5. 03 × 10^–5^	5.10 × 10^–5^
recovery, %		95.1 ± 1.2	95.4 ± 1.6	103.3 ± 5.6	111.8 ± 4.6

a average of four measurements.

b calculated with eq 2.

The selectivity test showed a very low influence of most tested inorganic anions, IB^–^ and Benz^–^ ions, on the potentiometric properties of **L1-**, **L4**-, and **L5**-based membranes. At the same time, less discrimination was observed between two target ions, NS^–^ and KF^–^, with the only exception for **L2**-based membranes, which show higher slopes toward KF^–^ ions compared to NS^–^ in a tiny concentration range from 10^–6^ to 10^–4^ mol/L (see Figure S19).

We have hence decided to address this problem by applying the multisensory approach since the utility of low-selective sensor arrays was previously demonstrated for NSAID assessment. Thus, for instance, the potentiometric sensor array based on the combination of six PVC membranes based on the sodium tetraphenylborate (NaTPB) cation-exchanger, TDMACl, tetrabutylammonium perchlorate (TBAP), and 3-aminophenylboronic acid hydrochloride (APBA) anion-exchangers, chloride ionophore II, and ETH 5350 chromoionophore III (used as pH-indicator) was developed to discriminate ibuprofen-based batch pharmaceuticals (Ibuflam 4%) with respect to their bitter/sweet taste characteristic changes.^12^ Upon the application of the principal component analysis (PCA) technique, the proposed potentiometric sensor array was able to indicate changes of bitterness and adding of masking excipients, such as sodium chloride and sweeteners, and to detect the slight changes in Ibuflam 4% samples’ taste. An application of a nonspecific sensor array with optical transduction based on highly fluorescent positively charged poly(para phenyleneethynylene), PPE, and its complex with a weakly fluorescent anionic pyridine containing poly(para-aryleneethynylene), PAE, serving as a quencher, at two different pHs 10 and pH 13, for the discrimination among “profens”, “salycilates”, “fenamic”, and “arylacetic” groups of painkillers, as well as their “counterfeits”, was reported by the Bunz group.^5^ The combination of hydrophobic and electrostatic interactions of the analytes with the PPE conjugated polymer and/or the PPE/PAE complexes resulted in fluorescence intensity variation of the sensor array that was interpreted by linear discriminant analysis (LDA).

With the purpose of identifying KF^–^ and NS^–^ anions, a small e-tongue array was prepared with sensors having membranes mb 1.3, mb 2.4, mb 4.3, and mb 5.1 (based on receptors **L1**, **L2**, **L4**, and **L5**, respectively), in two replicates, with eight sensors in total. While the membranes mb 1.3, mb 4.3, and mb 5.1 showed the highest sensitivity and potentiometric selectivity to the NS^–^ anion, the prevalent binding of KF^–^ anions by mb 2.4 based on the **L2** receptor was expected to give an important contribution to the e-tongue discrimination ability between KF^–^ and NS^–^ ions present in analyzed samples. The sensor responses were measured simultaneously in individual aqueous solutions of KF^–^ and NS^–^ of different concentrations (10^–7^, 10^–6^, 10^–5^ mol/L), as well as the pharmacological formulations (Oki and Synflex) containing as main components Lys-KF and NaNS salts.

**Figure 5:**
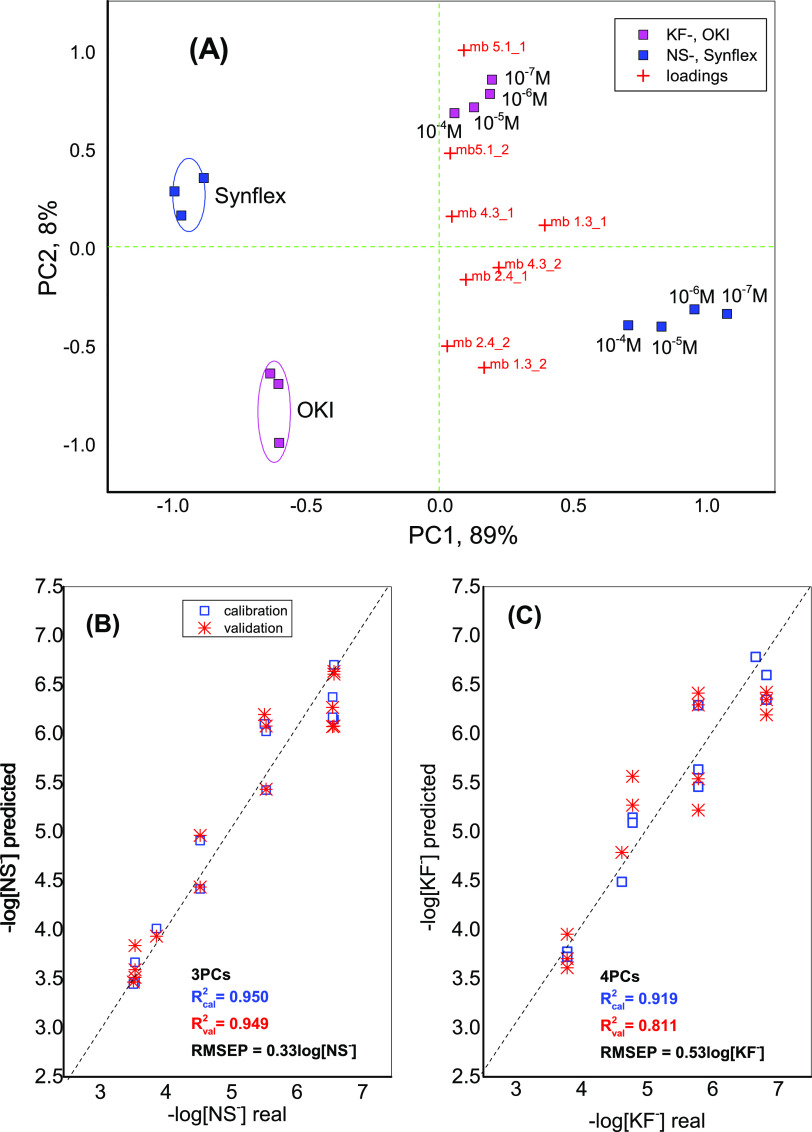
(A) PCA biplot (scores and loadings) of the **L1**–**L5**-based potentiometric e-tongue response in Lys-KF and NaNS aqueous solutions of different concentrations and in two different pharmacological formulations, based on these analytes (Oki and Synflex, respectively). PLS1 regression models for (B) NaNS and (C) Lys-KF. The points on the graph represent the mean value of six repeated measurements (*n* = 6).

As shown in Figure 5, the application of PCA to the numerical outputs of the e-tongue response permitted clear identification of all tested samples; the 97% total variance was explained by the first two PCs (principal components PC1 and PC2), and all of the tested membranes showed a significant influence in terms of loadings onto the analyzed sample identification. Finally, the PLS1 regression models were constructed to correlate the e-tongue output to the known concentrations of KF^–^ and NS^–^ in calibration solutions, and the possibility of detecting these analytes at concentrations as low as 0.15 and 0.07 μmol/L, with the correlation coefficient between the real and e-tongue predicted analyte concentrations of *R*^2^ = 0.947 (RMSEV 0.33 log[NS^–^], 3 PCs) for NaNS and *R*^2^ = 0.919 (RMSEV 0.53 log[KF^–^], 4 PCs) for Lys-KF, respectively, being demonstrated.

Finally, we compared the main characteristics of the developed NSAID sensors based on acyclic squaramides with those of the NS^–^-selective electrodes previously reported in the literature. The results are summarized in Table 4. The close-to-the-theoretical Nernstian slopes, the wide linear range of response, and the low detection limits of the developed ISEs based on acyclic squaramide ionophores **L1**–**L5** demonstrate their potential application as potentiometric sensors for NSAIDs.

**Table 4:** Comparison of NS^–^-Selective Electrodes Previously Reported in the Literature with the Developed NSAID Sensors/Sensory System Based on Acyclic Squaramides

lonophore	detection limit, mol/L	linear range, mol/L	slope, mV/dec	[refs]
methyltrioctylammonium	5 × 10^–5^	1 × 10^–4^–1 × 10^–1^	–59.3 ± 1.3	(13b)
tetraheptylammonium	1.4 × 10^–4^	1 × 10^–4^–1 × 10^–1^	–61.0	(13e)
tetraoctylammonium (S)-6-methoxy-a-methyl-2-naphthaleneacetate	2.95 × 10^–5^	1 × 10^–4^–1 × 10^–1^	–59.2 ± 1.7	(14c)
SPE/Calix/SWCNTs	1 × 10^–8^	1 × 10^–8^–1 × 10^–2^	–61.0 ± 0.6	(14a)
β-cyclodextrin	5 × 10^–5^	5 × 10^–5^–1 × 10^–2^	–59.0 ± 0.5	(17a)
L1/TDMACI, 1 equiv mb 1.3	1.2 × 10^–7^	1.0 × 10^–7^–1.0 × 10^–4^	–63.8 ± 2.3	this work
**sensor array**			PLS1 parameters:	this work
mb 1.3 (L1),	7.0 × 10^–8^	1.0 × 10^–7^–1.0 × 10^–4^	R^2^ = 0.947,	
mb 2.4 (L2),			RMSEV 0.33 log[NS^–^],	
mb 4.3 (L4),			3 PCs	
mb 5.1 (L5)				

## Conclusions

The obtained results demonstrate the potential use of novel acyclic squaramide receptors as selective hydrogen-bonding ionophores for the potentiometric detection of nonsteroidal anti-inflammatory drugs, NSAIDs. In particular, a small library of acyclic squaramides, **L1**–**L5**, has been investigated; solution-phase ^1^H NMR binding studies have shown a high affinity of the tested receptors toward KF^–^ and NS^–^ ions through the formation of H-bonds between the carboxylate groups of KF^–^ and NS^–^ and the receptors through indole and squaramide NH binding groups. The optimization of PVC-based polymeric membrane composition prepared with ionophores **L1**–**L5** was performed by varying the ligand/anion-exchanger ratio and evaluating the selectivity properties. The study has demonstrated the improved sensitivity and non-Hofmeister selectivity series for membranes based on acyclic squaramide ligands **L1**, **L4**, and **L5**. The best potentiometric properties for KF^–^ and NS^–^ ion sensing were shown by the **L1**-based membranes doped with 1 equiv of the TDMACl anion-exchanger (with respect to the ionophore), which have exhibited a close-to-Nernstian response of −63.8 mV/dec to the NS^–^ anion in the concentration range from 1.0 × 10^–7^ to 1.0 × 10^–4^ mol/L, confirming the 1:1 analyte/ionophore adduct formation. For membranes based on **L4** and **L5**, the significant increase in selectivity toward both KF^–^ and NS^–^ anions upon decreasing the amount of TDMACl to 0.3 and 0.5 equiv, respectively, indicated the formation of complexes with mixed stoichiometries (1:1, 1:2); the prevalent 1:2 analyte/ionophore binding for **L4** and **L5** is also confirmed by ^1^H NMR-binding studies in DMSO-*d*_6_/0.5% water and DMSO-*d*_6_/10% water solutions. The low solubility of **L5** inside the solvent polymeric membrane phase did not allow full characterization of the **L5** performance as a selective ionophore for NSAID anions. The **L2**-based membranes have demonstrated a slightly higher sensitivity (even if characterized with sub-Nernstian slopes) toward the smaller-sized nonlinear KF^–^ anion. We explained this behavior by considering the nonsymmetrical **L2** structure and the presence of EWGs, which contribute to the additional stabilization of the two benzene rings of the KF^–^ anion. On the other hand, for the receptor **L3**, with an open conformation and spatially separated and oppositely directed indole and squaramide NH groups, lower potentiometric responses toward NS^–^, and in particular toward the smaller KF^–^ ion, were observed due to the less effective receptor-anion binding.

The developed sensors were employed for a high precision detection of KF^–^ in pharmaceutical compositions, with relative errors of analysis, RSD%, as low as 0.99–1.4% and recoveries, R%, in the range of 95.1–111.8%. Additionally, the effectiveness of the potentiometric sensor array composed of four sensors based on ligands **L1, L2, L4**, and **L5** of optimized composition allowed the discrimination between structurally very similar KF^–^ and NS^–^ anions. The possibility of detecting these analytes at concentrations as low as 0.07 μmol/L with *R*^2^ of 0.947 and at 0.15 μmol/L with *R*^2^ of 0.919 for NS^–^ and KF^–^, respectively, was shown, thus indicating the utility of the potentiometric e-tongue based on acyclic squaramide receptors as a fast and indirect tool for the screening and discrimination of anti-inflammatory KF^–^ and NS^–^ pharmaceuticals and opening new perspectives for the assessment and control of these drugs.

## Supporting Information Available

NMR spectra; ^1^H NMR titrations; and potentiometric testing data (PDF)

## References

[ref1] Brooks P. M.; Day R. O.; et al. Nonsteroidal Antiinflammatory Drugs — Differences and Similarities. N. Engl. J. Med. 1991, 324, 1716–1725. 10.1056/NEJM199106133242407. 2034249

[ref2] https://www.nhs.uk/conditions/nsaids/.

[ref3] Nishi I.; Kawakami T.; Onodera S. Monitoring the concentrations of nonsteroidal anti-inflammatory drugs and cyclooxygenase-inhibiting activities in the surface waters of the Tone Canal and Edo River Basin. J. Environ. Sci. Health, Part A 2015, 50, 1108–1115. 10.1080/10934529.2015.1047647. 26191985

[ref4] aEslami A.; Amini M. M.; Yazdanbakhsh A. R.; Rastkari N.; Mohseni-Bandpei A.; Nasseri S.; Piroti E.; Asadi A. Occurrence of non-steroidal anti-inflammatory drugs in Tehran source water, municipal and hospital wastewaters, and their ecotoxicological risk assessment. Environ. Monit. Assess. 2015, 187, 734 10.1007/s10661-015-4952-1. 26553436

[ref5] Han J.; Wang B.; Bender M.; Kushida S.; Seehafer K.; Bunz U. H. F. Poly(aryleneethynylene) Tongue That Identifies Nonsteroidal Anti-Inflammatory Drugs in Water: A Test Case for Combating Counterfeit Drugs. ACS Appl. Mater. Interfaces 2017, 9, 790–797. 10.1021/acsami.6b11690. 27982567

[ref6] aFan Y.; Feng Y.-Q.; Da S.-L.; Wang Z.-H. In-tube solid phase microextraction using a β-cyclodextrin coated capillary coupled to high performance liquid chromatography for determination of non-steroidal anti-inflammatory drugs in urine samples. Talanta 2005, 65, 111–117. 10.1016/j.talanta.2004.05.040. 18969772

[ref7] aEl-Sadek M.; El-Adl S.; Abou-Kull M.; Sakr S. M. Spectrophotometric determination of ketoprofen in pharmaceutical preparations by means of charge transfer complex formation. Talanta 1993, 40, 585–588. 10.1016/0039-9140(93)80021-I. 18965670

[ref8] Lenik J. Application of PVC in Construction of Ion-Selective Electrodes for Pharmaceutical Analysis: A Review of Polymer Electrodes for Nonsteroidal, Anti-Inflammatory Drugs. In Handbook of Polymers for Pharmaceutical Technologies, 2015; pp 195–227.

[ref9] aEssam H. M.; Bassuoni Y. F.; Elzanfaly E. S.; Zaazaa H. E.-S.; Kelani K. M. Potentiometric sensing platform for selective determination and monitoring of codeine phosphate in presence of ibuprofen in pharmaceutical and biological matrices. Microchem. J. 2020, 159, 105286 10.1016/j.microc.2020.105286.

[ref10] Lenik J.; Nieszporek J. Construction of a glassy carbon ibuprofen electrode modified with multi-walled carbon nanotubes and cyclodextrins. Sens. Actuators, B 2018, 255, 2282–2289. 10.1016/j.snb.2017.09.034.

[ref11] Alizadeh N.; Samaei E.; Kalhor H. Electrochemically controlled solid phase microextraction of ibuprofen based on nanostructure conducting molecular imprinted polypyrrole and selective analysis in biological and formulation samples using ion mobility spectrometry. Anal. Methods 2014, 6, 2909–2915. 10.1039/c3ay42090f.

[ref12] Shishkanova T. V.; Broncová G.; Skálová A.; Prokopec V.; Člupek M.; Kral V. Potentiometric Electronic Tongue for Taste Assessment of Ibuprofen Based Pharmaceuticals. Electroanalysis 2019, 31, 2024–2031. 10.1002/elan.201900334.

[ref13] aAhmed D. A.; El-Rahman M. K. A.; Lotfy H. M.; Weshahy S. A. Double-Dip Approach: Simultaneous Dissolution Profiling of Pseudoephedrine and Ibuprofen in a Combined Dosage Form by Ion Selective Electrodes. J. Electrochem. Soc. 2018, 165, H999 10.1149/2.1291814jes.

[ref14] aLenik J. Properties of ion-selective electrodes with polymeric membranes for ketoprofen determination. J. Anal. Chem. 2012, 67, 543–549. 10.1134/S106193481206007X.

[ref15] Khaled E.; Shoukry E. M.; Amin M. F.; Said B. A. M. Novel Calixarene/Carbon Nanotubes Based Screen Printed Sensors for Flow Injection Potentiometric Determination of Naproxen. Electroanalysis 2018, 30, 2878–2887. 10.1002/elan.201800602.

[ref16] Hassan S. S. M.; Mahmoud W. H.; Elmosallamy M. A. F.; Almarzooqi M. H. Novel Ibuprofen Potentiometric Membrane Sensors Based on Tetraphenylporphyrinato Indium(III). Anal. Sci. 2003, 19, 675–679. 10.2116/analsci.19.675. 12769363

[ref17] aJunco S.; Casimiro T.; Ribeiro N.; Nunes Da Ponte M.; Cabral Marques H. A comparative study of naproxen–beta cyclodextrin complexes prepared by conventional methods and using supercritical carbon dioxide. J. Inclusion Phenom. Macrocyclic Chem. 2002, 44, 117–121. 10.1023/A:1023022008337.

[ref18] Nazarov V. A.; Sokolova E.; Andronchik K.; Egorov V.; Belyaev S.; Yurkshtovich T. Ibuprofen-selective electrode on the basis of a neutral carrier, N-trifluoroacetylbenzoic acid heptyl ester. J. Anal. Chem. 2010, 65, 960–963. 10.1134/S1061934810090121.

[ref19] aIan Storer R.; Aciro C.; Jones L. H. Squaramides: physical properties, synthesis and applications. Chem. Soc. Rev. 2011, 40, 2330–2346. 10.1039/c0cs00200c. 21399835

[ref20] aAmendola V.; Bergamaschi G.; Boiocchi M.; Fabbrizzi L.; Milani M. The Squaramide versus Urea Contest for Anion Recognition. Chem. - Eur. J. 2010, 16, 4368–4380. 10.1002/chem.200903190. 20222093

[ref21] aBusschaert N.; Kirby I. L.; Young S.; Coles S. J.; Horton P. N.; Light M. E.; Gale P. A. Squaramides as Potent Transmembrane Anion Transporters. Angew. Chem., Int. Ed. 2012, 51, 4426–4430. 10.1002/anie.201200729. 22461434

[ref22] aElmes R. B.; Jolliffe K. A. Amino acid-based squaramides for anion recognition. Supramol. Chem. 2015, 27, 321–328. 10.1080/10610278.2014.976221.

[ref23] Jin C.; Zhang M.; Deng C.; Guan Y.; Gong J.; Zhu D.; Pan Y.; Jiang J.; Wang L. Novel calix[4]arene-based receptors with bis-squaramide moieties for colorimetric sensing of anions via two different interaction modes. Tetrahedron Lett. 2013, 54, 796–801. 10.1016/j.tetlet.2012.11.117.

[ref24] aProhens R.; Tomàs S.; Morey J.; Deyà P. M.; Ballester P.; Costa A. Squaramido-based receptors: Molecular recognition of carboxylate anions in highly competitive media. Tetrahedron Lett. 1998, 39, 1063–1066. 10.1016/S0040-4039(97)10728-6.

[ref25] Picci G.; Kubicki M.; Garau A.; Lippolis V.; Mocci R.; Porcheddu A.; Quesada R.; Ricci P. C.; Scorciapino M. A.; Caltagirone C. Simple squaramide receptors for highly efficient anion binding in aqueous media and transmembrane transport. Chem. Commun. 2020, 56, 11066–11069. 10.1039/D0CC04090H. 32812561

[ref26] aFan L.; Xu T.; Feng J.; Ji Z.; Li L.; Shi X.; Tian C.; Qin Y. Tripodal Squaramide Derivative as a Neutral Chloride Ionophore for Whole Blood and Sweat Chloride Measurement. Electroanalysis 2020, 32, 805–811. 10.1002/elan.201900693.

[ref27] Picci G.; Carreira-Barral I.; Alonso-Carrillo D.; Busonera C.; Milia J.; Quesada R.; Caltagirone C. The role of indolyl substituents in squaramide-based anionophores. Org. Biomol. Chem. 2022, 20, 7981–7986. 10.1039/D2OB01444K. 36196986

[ref28] Picci G.; Aragoni M. C.; Arca M.; Caltagirone C.; Formica M.; Fusi V.; Giorgi L.; Ingargiola F.; Lippolis V.; Macedi E.; Mancini L.; Mummolo L.; Prodi L. Fluorescent sensing of non-steroidal anti-inflammatory drugs naproxen and ketoprofen by dansylated squaramide-based receptors. Org. Biomol. Chem. 2023, 21, 2968–2975. 10.1039/D3OB00324H. 36938589

[ref29] Rostami A.; Colin A.; Li X. Y.; Chudzinski M. G.; Lough A. J.; Taylor M. S. N,N′-Diarylsquaramides: General, High-Yielding Synthesis and Applications in Colorimetric Anion Sensing. J. Org. Chem. 2010, 75, 3983–3992. 10.1021/jo100104g. 20486682

[ref30] aBrynn Hibbert D.; Thordarson P. The death of the Job plot, transparency, open science and online tools, uncertainty estimation methods and other developments in supramolecular chemistry data analysis. Chem. Commun. 2016, 52, 12792–12805. 10.1039/C6CC03888C. 27779264

[ref31] Lvova L.; Natale C. D.; D’Amico A.; Paolesse R. Corrole-based ion-selective electrodes. J. Porphyrins Phthalocyanines 2009, 13, 1168–1178. 10.1142/S1088424609001510.

[ref32] Umezawa Y.; Umezawa K.; Sato H. Selectivity coefficients for ion-selective electrodes: Recommended methods for reporting KA, Bpot values (Technical Report). Pure Appl. Chem. 1995, 67, 507–518. 10.1351/pac199567030507.

[ref33] Levitchev S. S.; Smirnova A. L.; Khitrova V. L.; Lvova L. B.; Bratov A. V.; Vlasov Y. G. Photocurable carbonate-selective membranes for chemical sensors containing lipophilic additives. Sens. Actuators, B 1997, 44, 397–401. 10.1016/S0925-4005(97)00232-3.

[ref34] Lvova L.; K D.; Legin A.; Di Natale C. Multisensor Systems for Chemical Analysis, 1st ed.; Jenny Stanford Publishing, 2014.

[ref35] Aramini A.; Bianchini G.; Lillini S.; Bordignon S.; Tomassetti M.; Novelli R.; Mattioli S.; Lvova L.; Paolesse R.; Chierotti M. R.; Allegretti M. Unexpected Salt/Cocrystal Polymorphism of the Ketoprofen–Lysine System: Discovery of a New Ketoprofen–l-Lysine Salt Polymorph with Different Physicochemical and Pharmacokinetic Properties. Pharmaceuticals 2021, 14, 555 10.3390/ph14060555. 34200917 PMC8230491

[ref36] Eugster R.; Rosatzin T.; Rusterholz B.; Aebersold B.; Pedrazza U.; Rüegg D.; Schmid A.; Spichiger U. E.; Simon W. Plasticizers for liquid polymeric membranes of ion-selective chemical sensors. Anal. Chim. Acta 1994, 289, 1–13. 10.1016/0003-2670(94)80001-4.

[ref37] Rotger M. C.; Piña M. N.; Frontera A.; Martorell G.; Ballester P.; Deyà P. M.; Costa A. Conformational preferences and self-template macrocyclization of squaramide-based foldable modules. J. Org. Chem. 2004, 69, 2302–2308. 10.1021/jo035546t. 15049622

[ref38] Prohens R.; Portell A.; Puigjaner C.; Tomas S.; Fujii K.; Harris K. D.; Alcobe X.; Font-Bardia M.; Barbas R. Cooperativity in solid-state squaramides. Cryst. Growth Des. 2011, 11, 3725–3730. 10.1021/cg200772e.

[ref39] aBakker E.; Bühlmann P.; Pretsch E. The phase-boundary potential model. Talanta 2004, 63, 3–20. 10.1016/j.talanta.2003.10.006. 18969400

[ref40] aSeah G. E. K. K.; Tan A. Y. X.; Neo Z. H.; Lim J. Y. C.; Goh S. S. Halogen Bonding Ionophore for Potentiometric Iodide Sensing. Anal. Chem. 2021, 93, 15543–15549. 10.1021/acs.analchem.1c03719. 34767713

