# Supplementary material for: Potentiometric Sensing of Nonsteroidal Painkillers by Acyclic Squaramide Ionophores
Source: ACS Sens. 2023 Aug 2;8(8):3225–39. doi: 10.1021/acssensors.3c00981 (PMC10463271; doi:10.1021/acssensors.3c00981)
Supplement: Supplementary file 1 — se3c00981_si_001.pdf [file se3c00981_si_001.pdf]

Potentiometric Sensing of Non-Steroidal Painkillers by Acyclic Squaramide Ionophores

Giacomo Picci[1], Sara Farotto[2], Jessica Milia[1], Claudia Caltagirone[1,*], Vito Lippolis[1], Maria Carla Aragoni[1], Corrado Di Natale[3], Roberto Paolesse[2], Larisa Lvova[2'*].

[1] Dipartimento di Scienze Chimiche e Geologiche, Università degli Studi di Cagliari, S.S. 554 Bivio per Sestu, 09042 Monserrato (CA), Italy. *E-mail: ccaltagirone@unica.it.

2 Department of Chemical Science and Technologies, University of Rome "Tor Vergata", 00133 Rome, Italy. *E-mail: larisa.lvova@uniroma2.it.

3 Department of Electronic Engineering, University of Rome "Tor Vergata", 00133 Rome, Italy.

SUPPORTING INFORMATION

1. **NMR SPECTRA** page 2
2. **^{1}H-NMR TITRATIONS** page 6
3. **POTENTIOMETRIC TESTING** page 26
   3.1 ***Super-Nernstian response of L1-doped membranes*** page 28
   3.2 ***Phase-boundary model calculations*** page 30

1. NMR Spectra

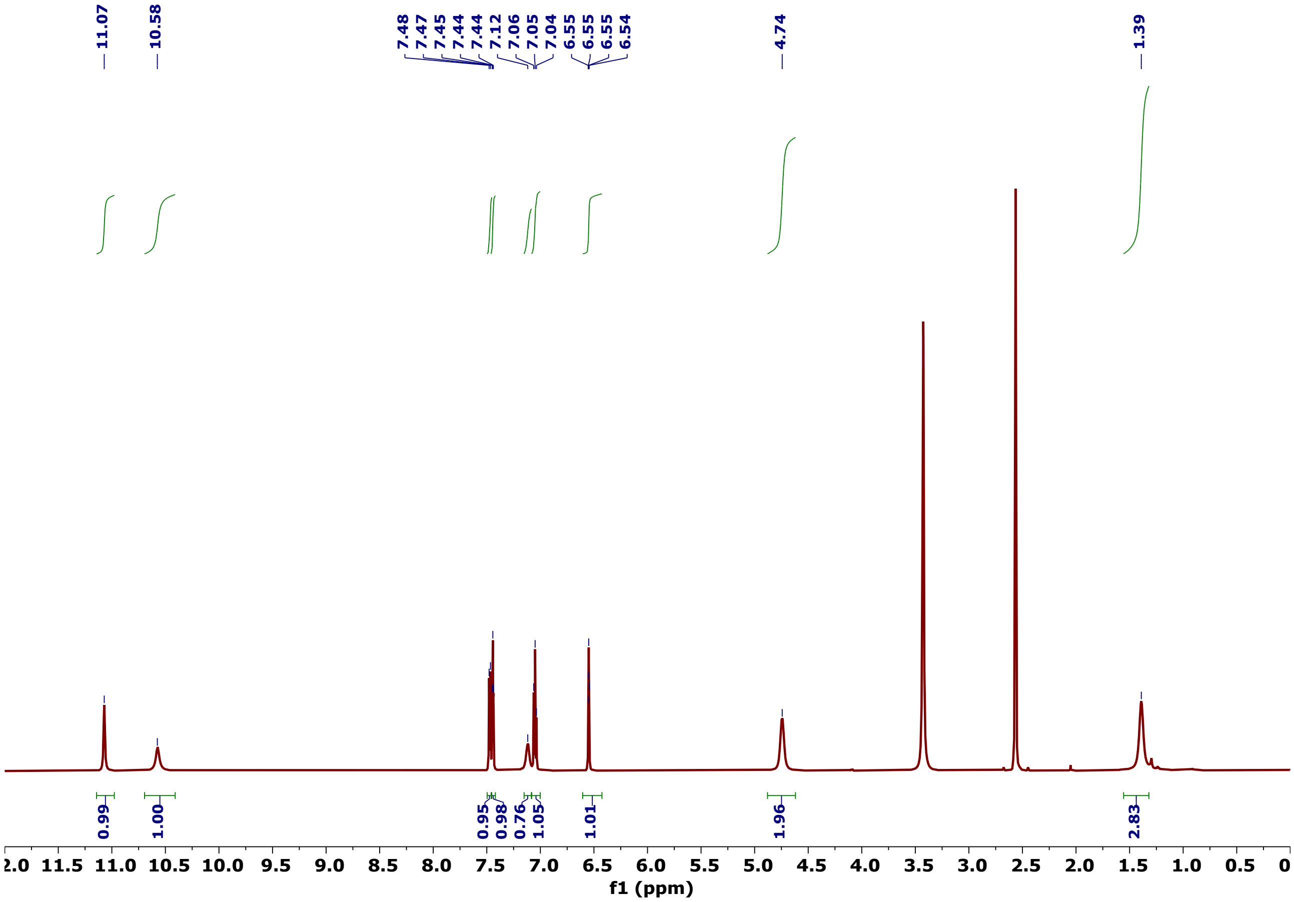

Figure S1 ^{1}H-NMR of **1** in DMSO-d_6.

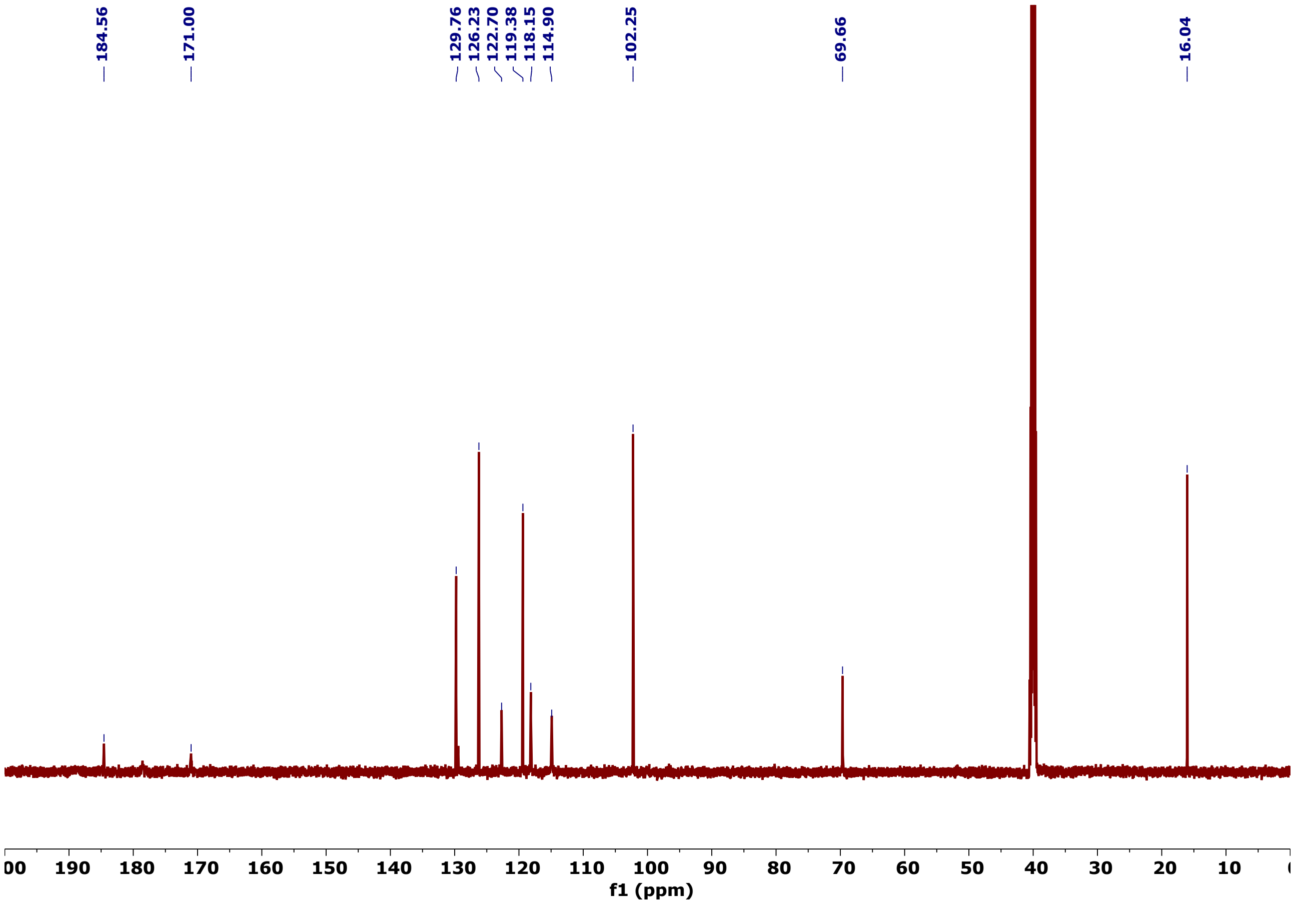

Figure S2 ^{13}C NMR of **1** in DMSO-d_6.

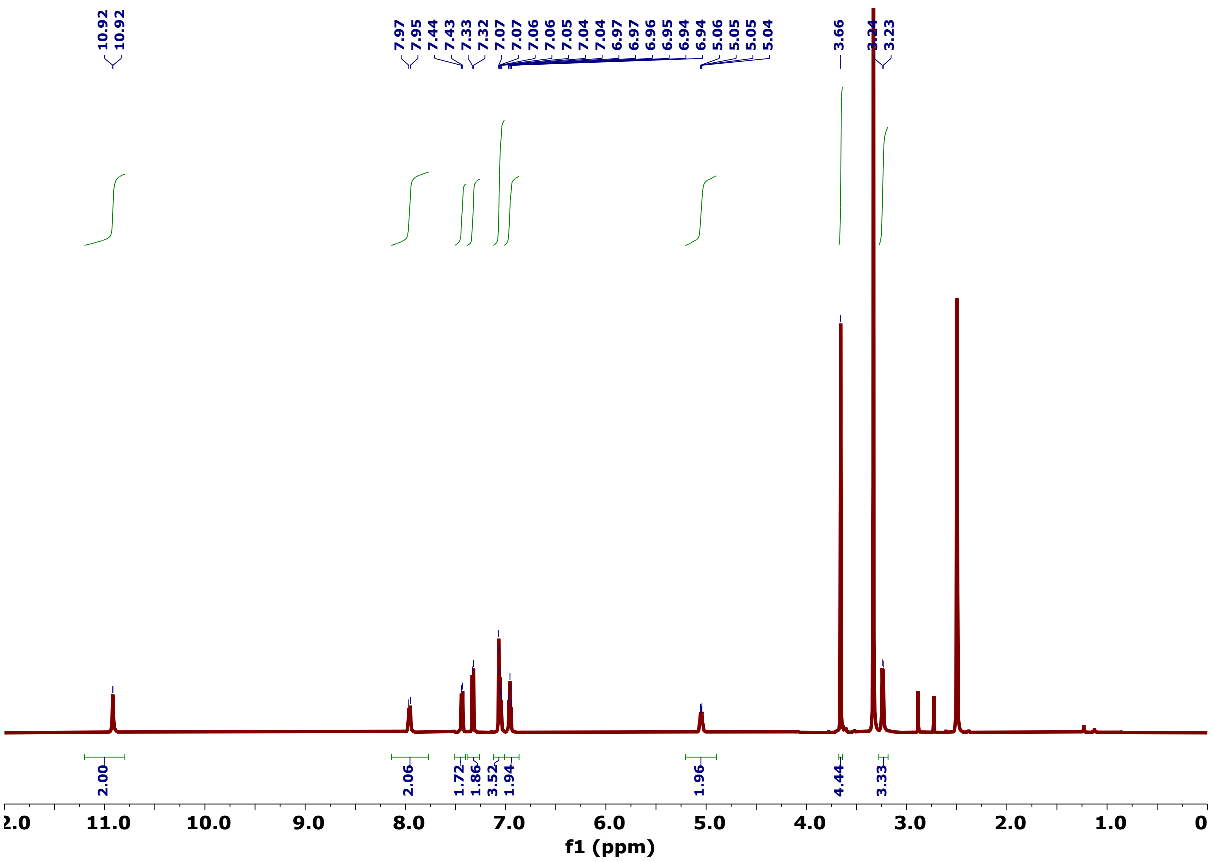

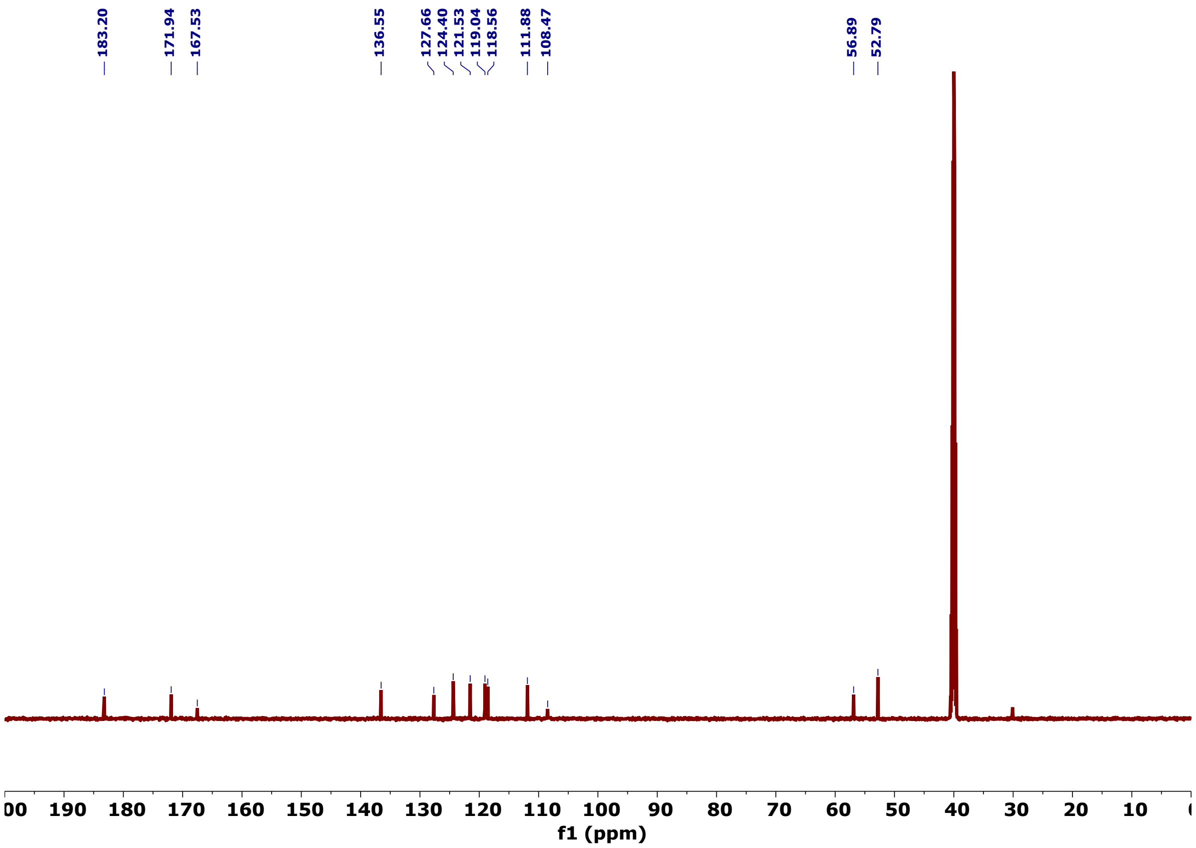

Figure 3. ^{1}H NMR and ^{13}C NMR spectra of **L3** in DMSO-d_6

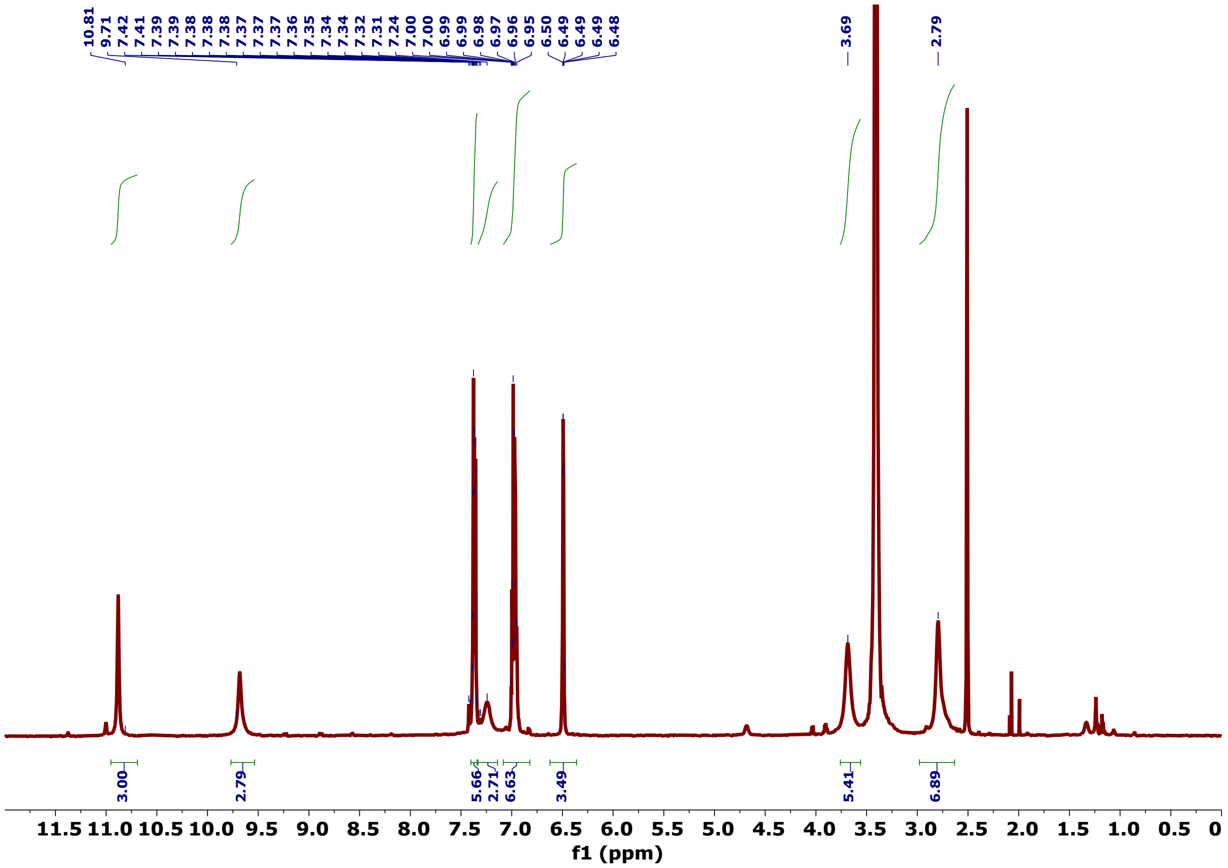

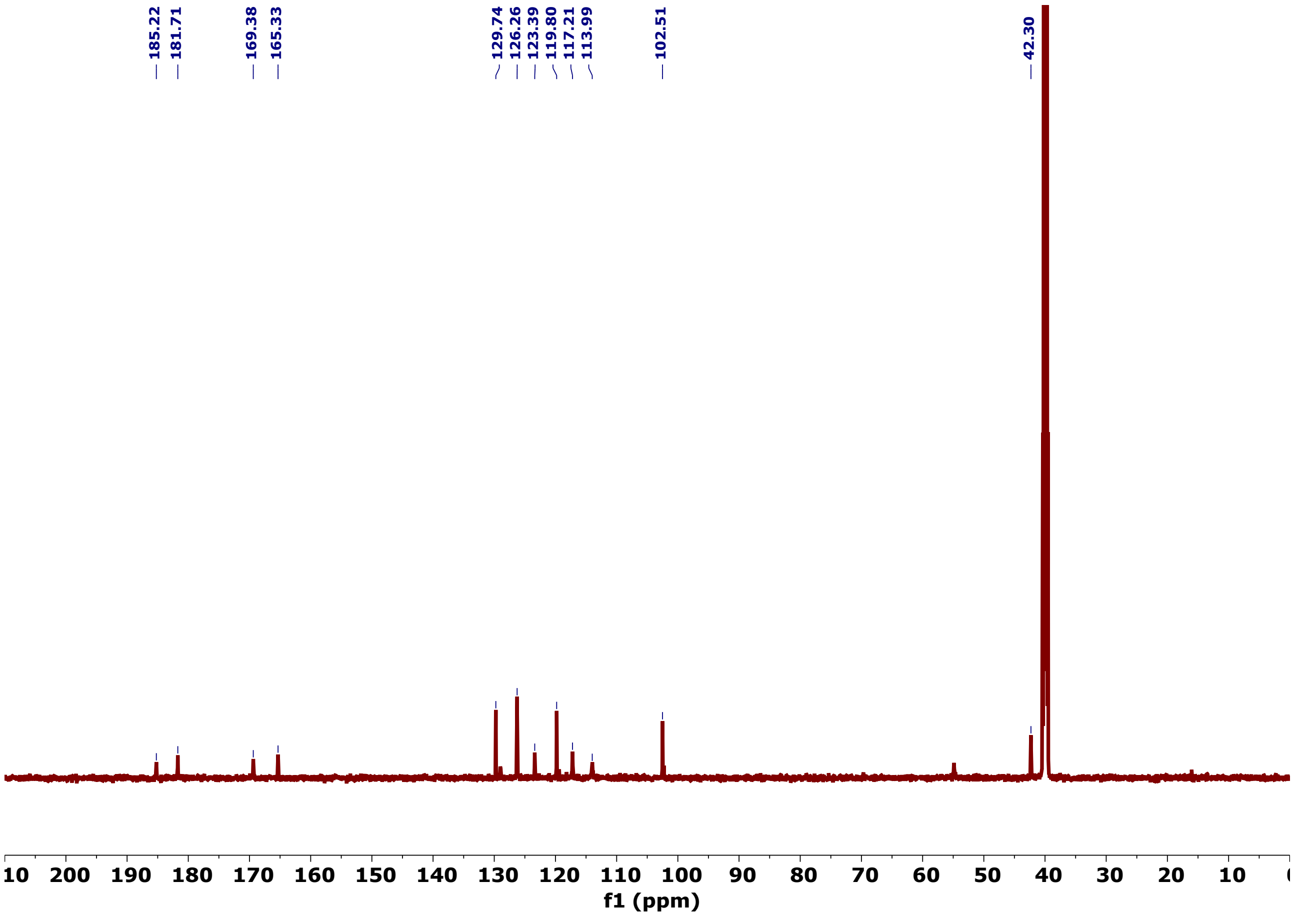

Figure S4. ^{1}H NMR and ^{13}C NMR spectra of **L4** in DMSO-d_6

2. ^{1}H-NMR Titrations

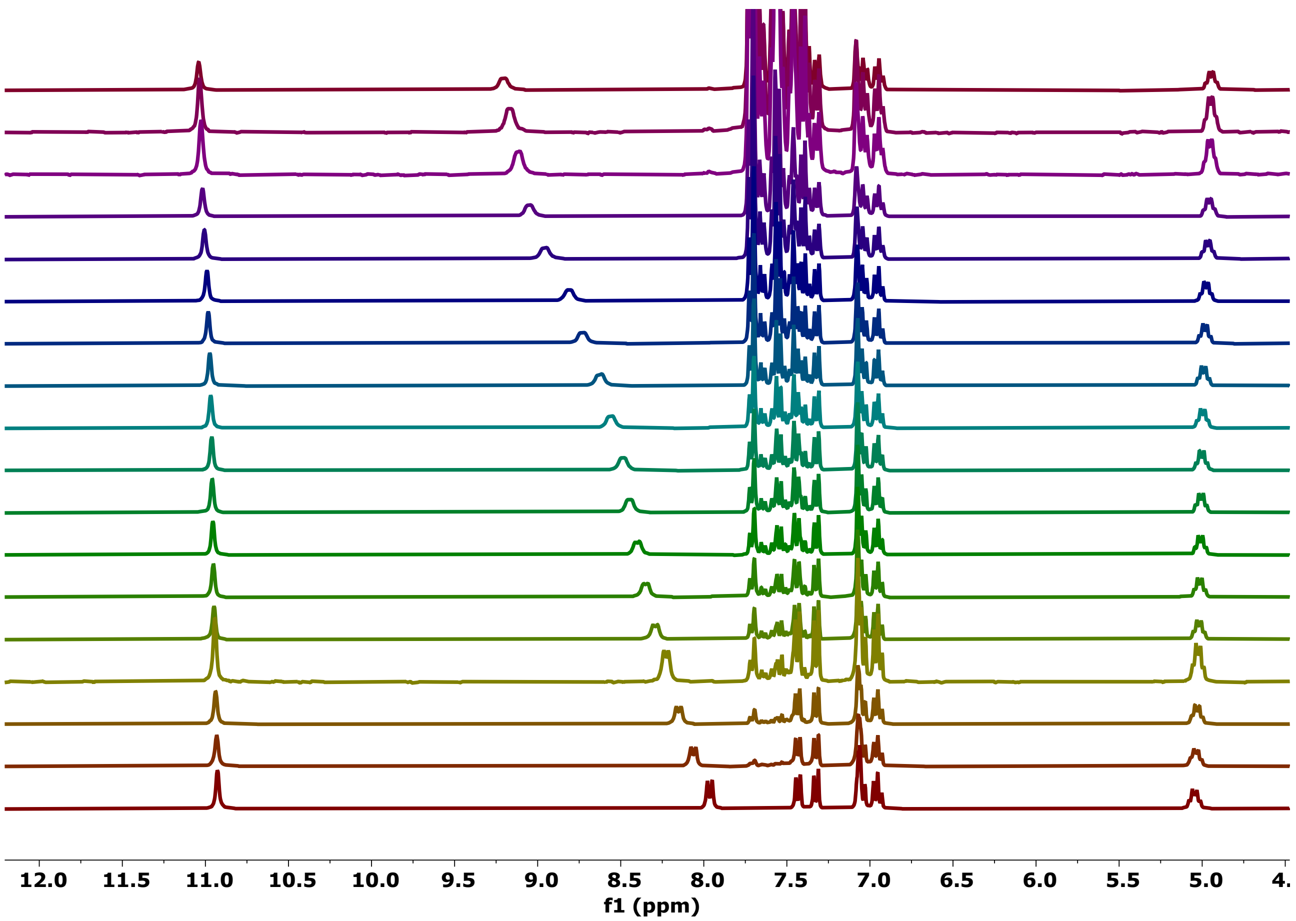

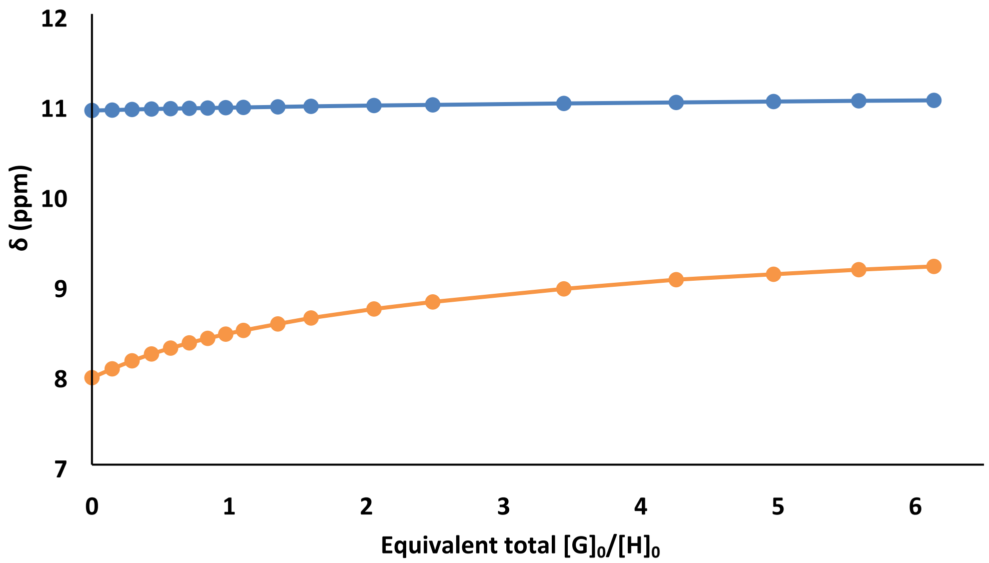

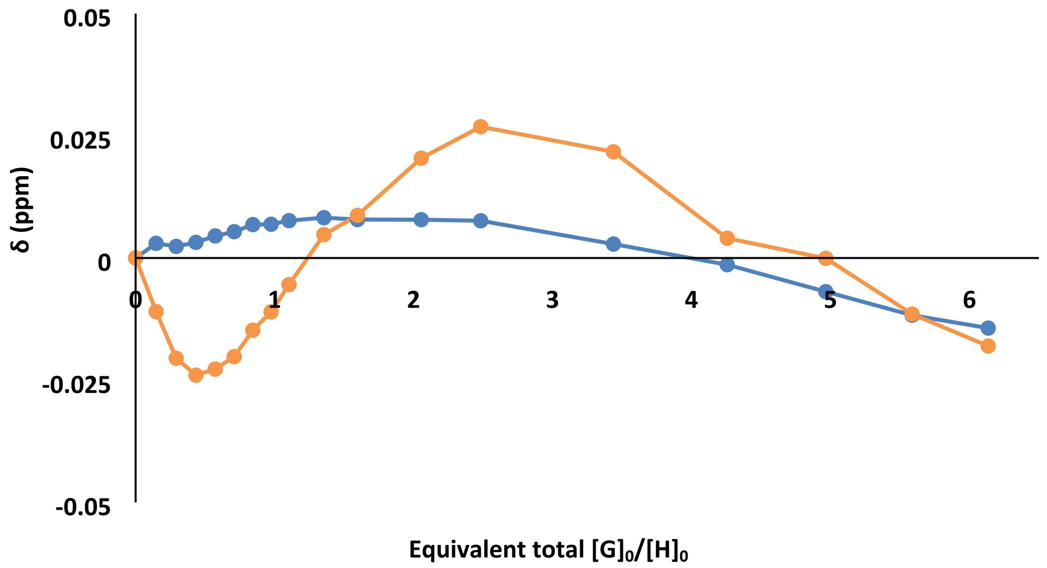

K	K error (%)	SSR	Datapoints fitted	Params fitted	H coeffs	HG coeffs	Raw coeffs 1	Raw coeffs 2
125,753913	2,45300876	0,00546617	36	3	10,929	11,0553237	10,929	11,0553237
					7,965	9,53786364	7,965	9,53786364

http://app.supramolecular.org/bindfit/view/a7c07b3a-3edc-4988-af0d-067e9081bea2

Figure S5. ^{1}H NMR titration of **L3** (5.0×10^{-3} mol/L) with NaKF (7.5×10^{-3} mol/L) in DMSO-d_6/0.5% water

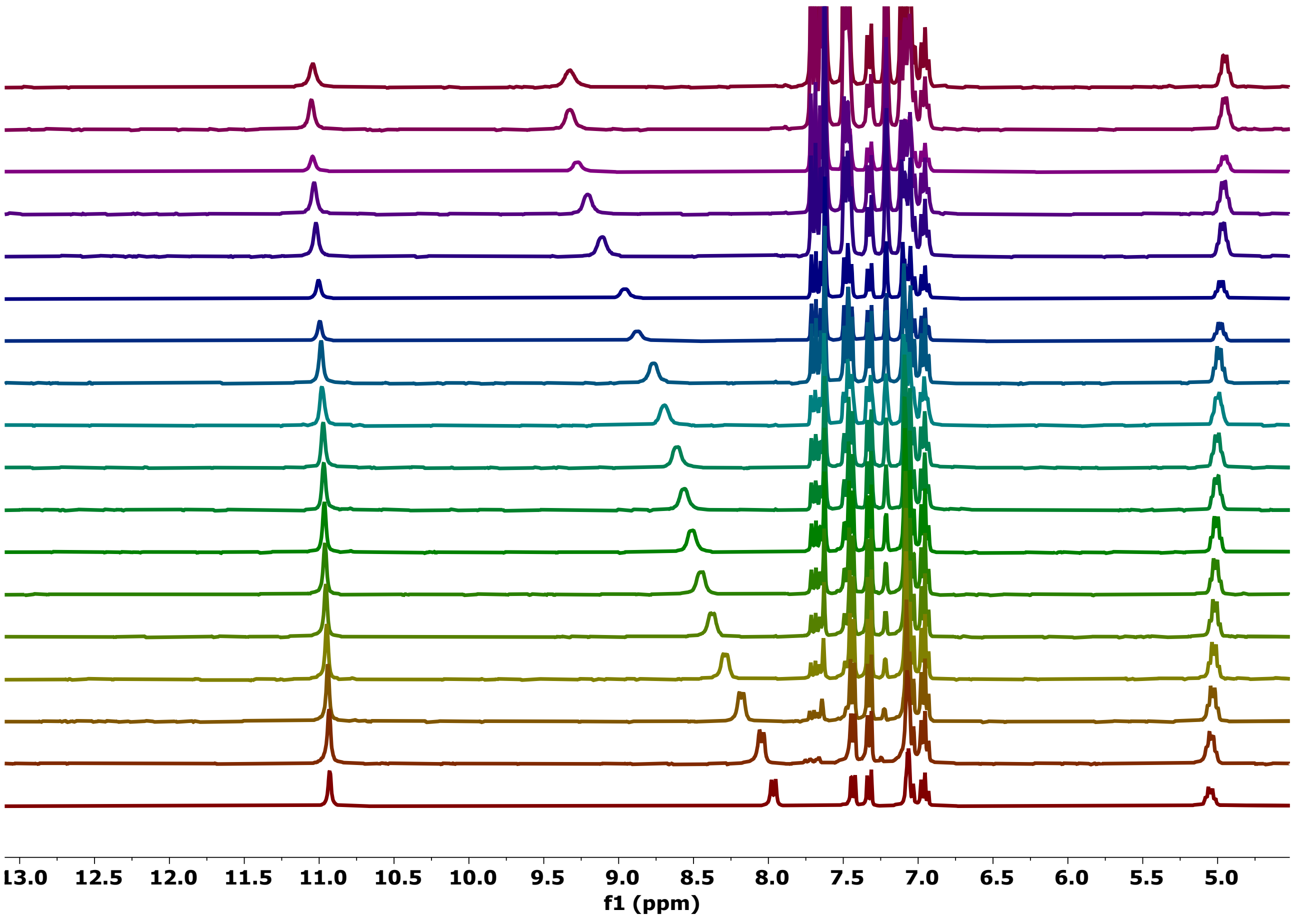

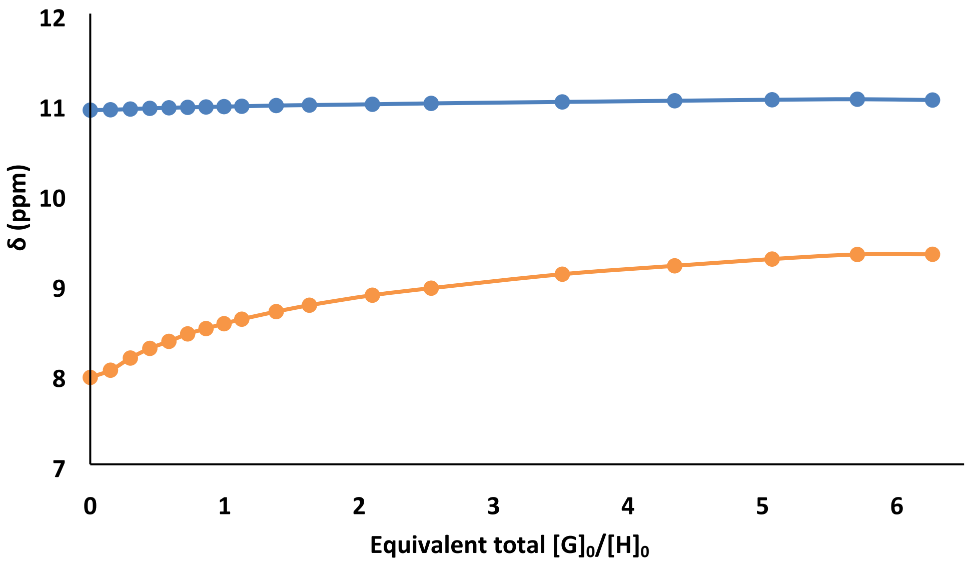

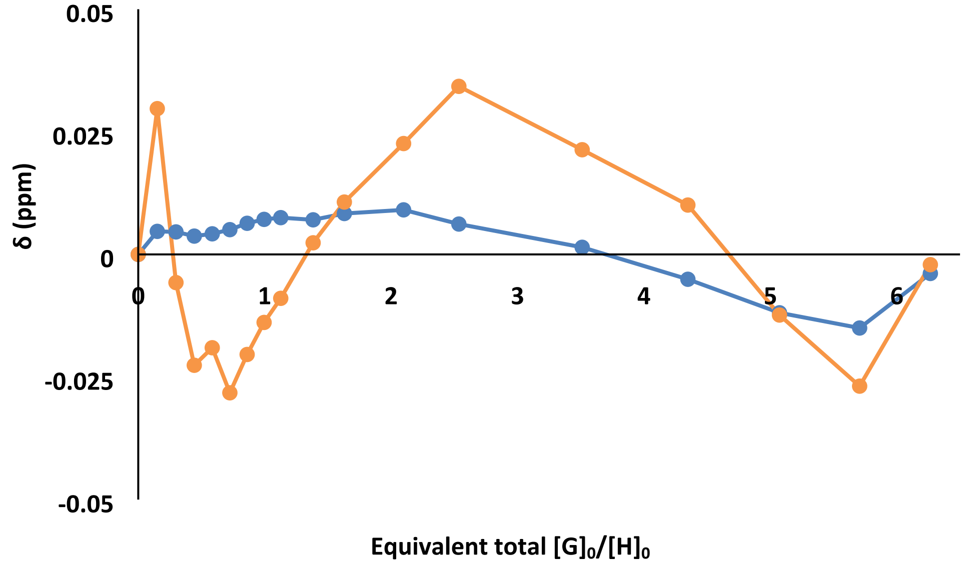

K	K error (%)	SSR	Datapoints fitted	Params fitted	H coeffs	HG coeffs	Raw coeffs 1	Raw coeffs 2
164,811178	2,78733585	0,00741462	36	3	10,929	11,0609487	10,929	11,0609487
					7,964	9,62862783	7,964	9,62862783

http://app.supramolecular.org/bindfit/view/9e8f34e0-0b57-4708-96b1-70a450a56a1a

Figure S6. ^{1}H NMR titration of **L3** (5.0×10^{-3} mol/L) with NaNS (7.5×10^{-3} mol/L) in DMSO-d_6/0.5% water

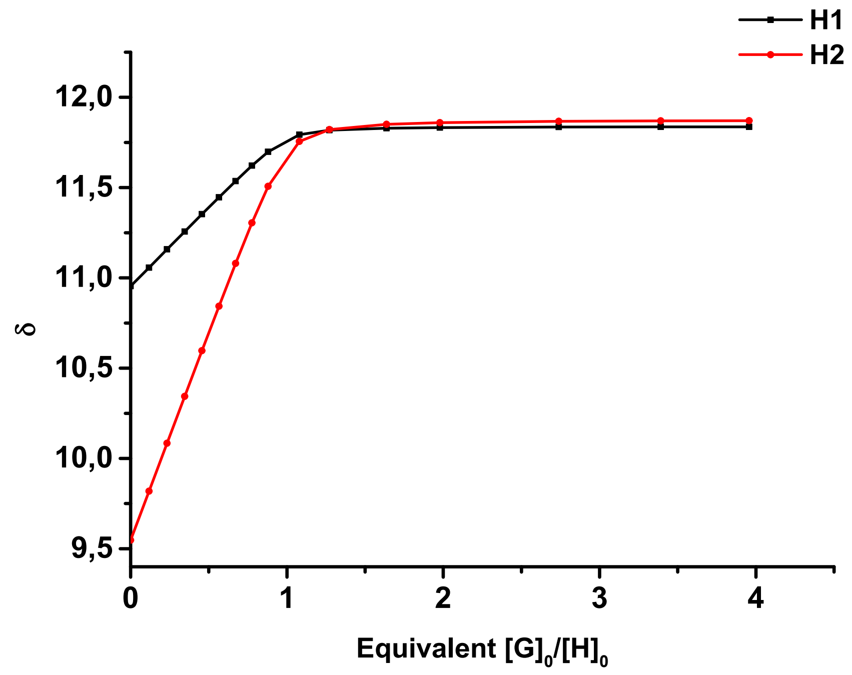

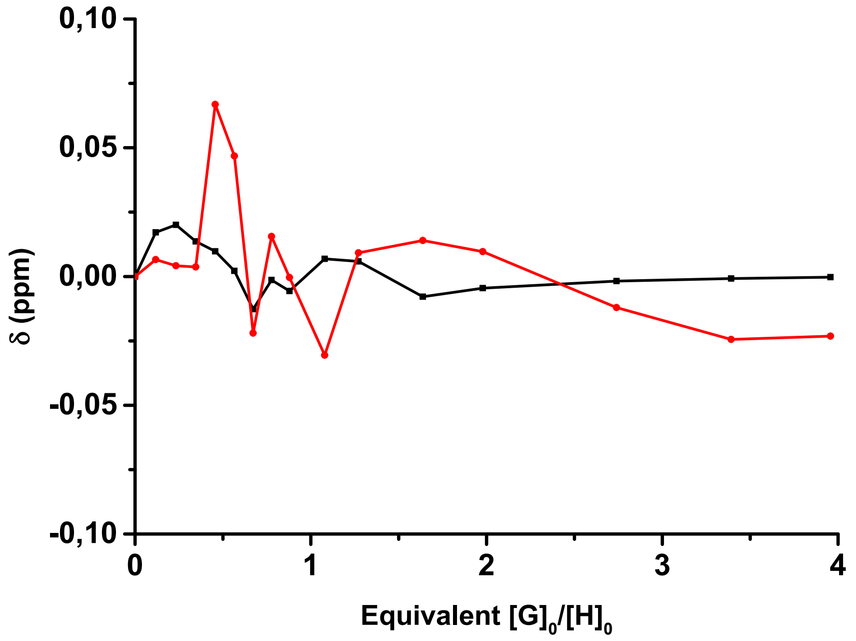

K	K error (%)	SSR	Datapoints fitted	Params fitted	H coeffs	HG coeffs	Raw coeffs 1	Raw coeffs 2
21528.0762	27.9568387	0.01138255	32	3	10.954	11.8388702	10.954	11.8388702
					9.547	11.876503	9.547	11.876503

http://app.supramolecular.org/bindfit/view/287ccf30-8e95-494c-8067-7f48055ecf9a

Figure S7. ^{1}H NMR titration of **L1** (5.0×10^{-3} mol/L) with NaKF (7.5×10^{-3} mol/L) in DMSO-d_6/0.5% water

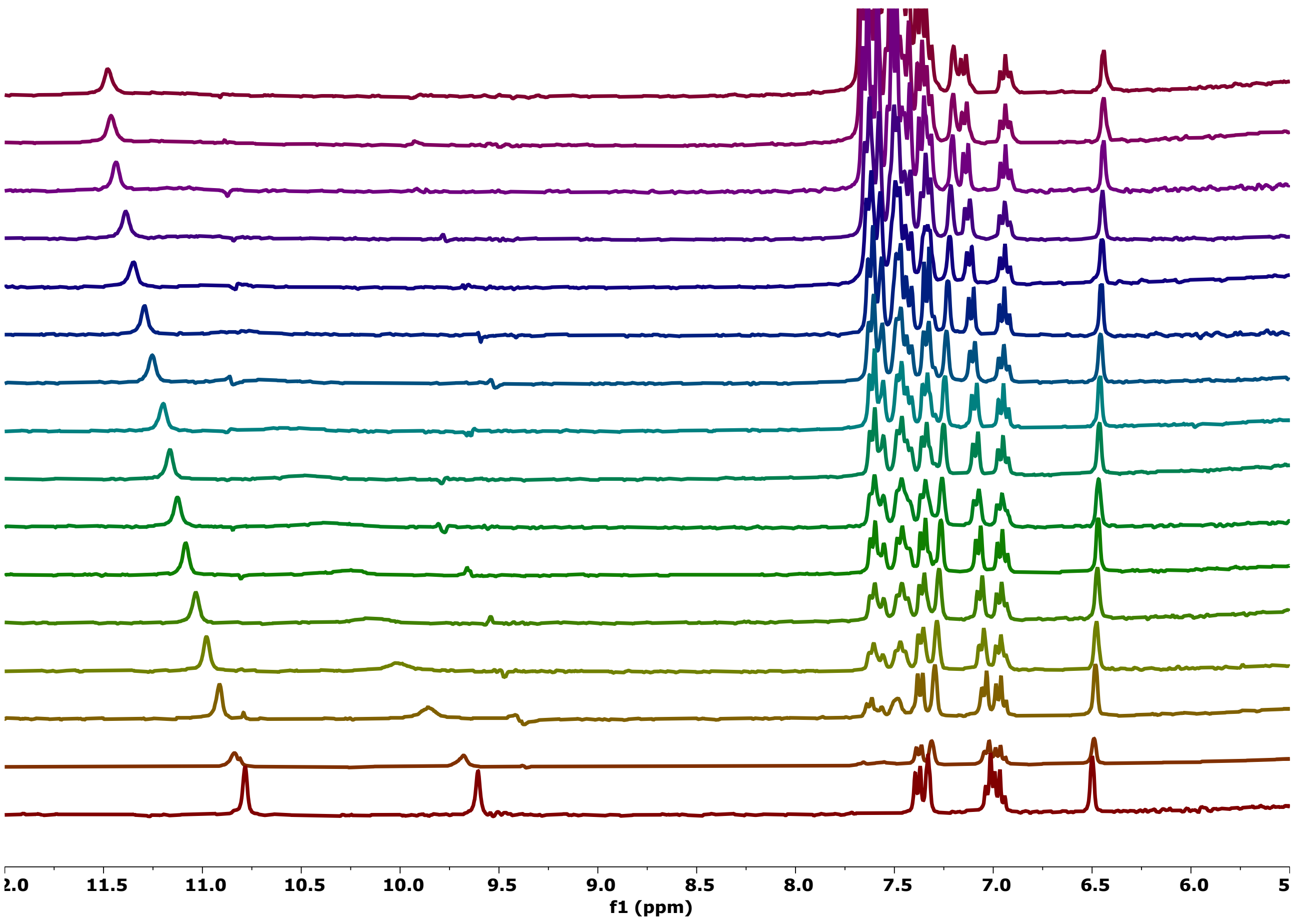

2.0
11.5
11.0
10.5
10.0
9.5
9.0
8.5
8.0
7.5
7.0
6.5
6.0
5
f1 (ppm)

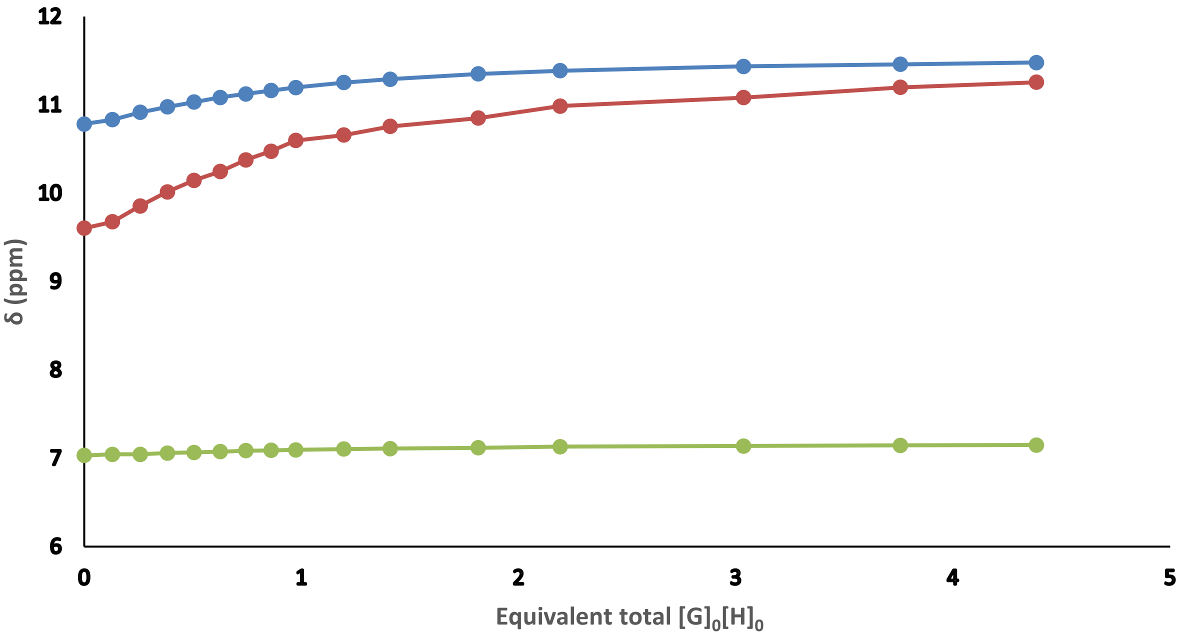

12
11
10
9
8
7
6
δ (ppm)
0
1
2
3
4
5
Equivalent total [G]0[H]0

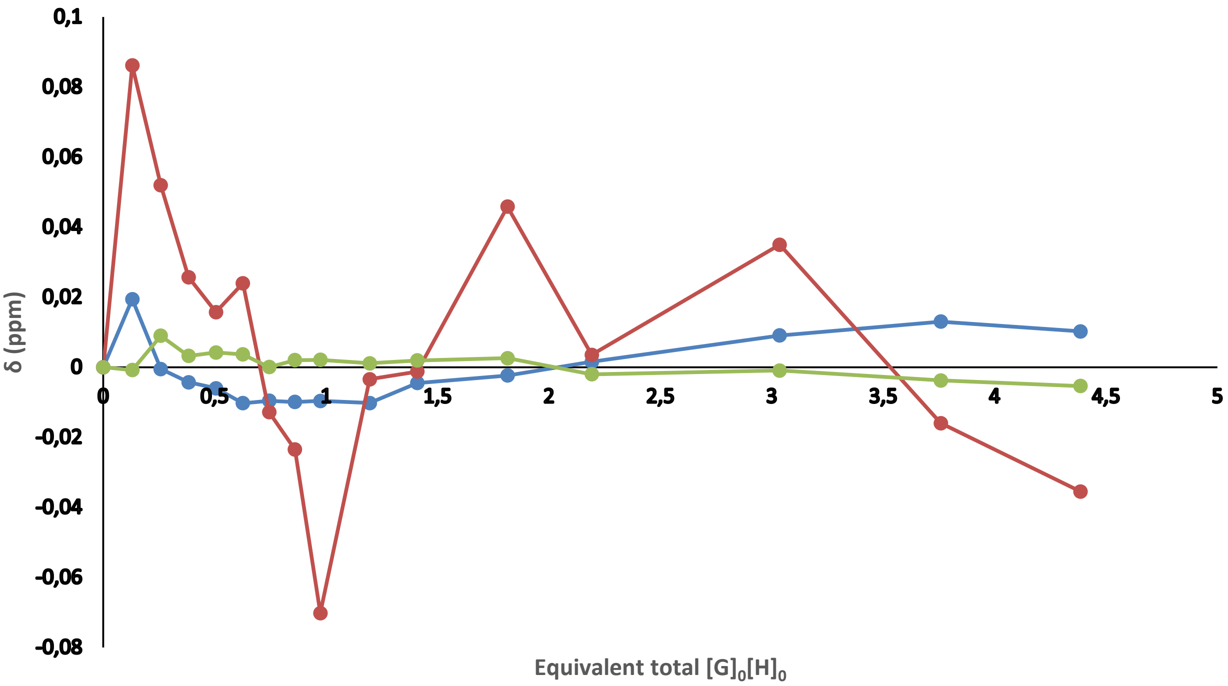

http://app.supramolecular.org/bindfit/view/9d816369-cb5c-4c03-9b4a-75c4b7be8bde

Figure S8. ^{1}H NMR titration of **L1** (5.0×10^{-3} mol/L) with NaKF (7.5×10^{-3} mol/L) in DMSO-d_6/10% water

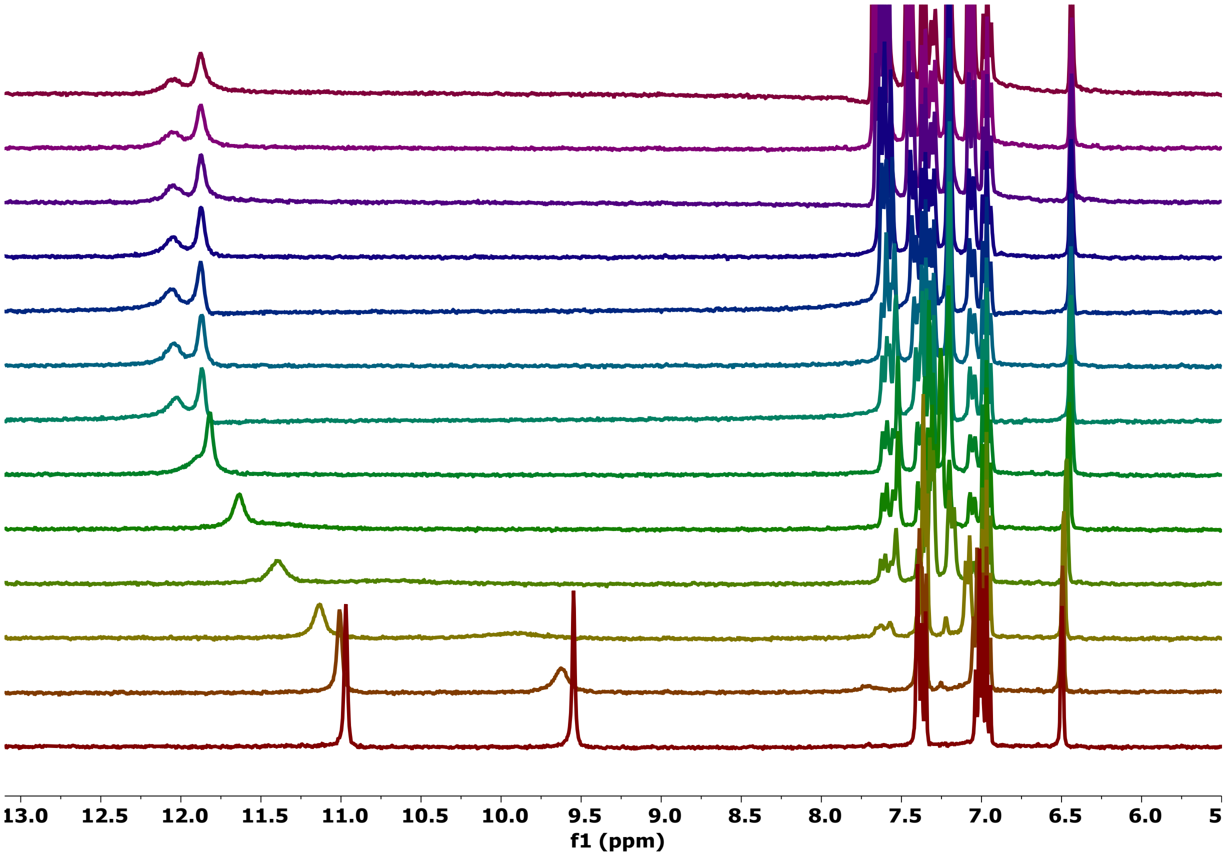

Figure S9 Stack plot of the ^{1}H NMR titration of **L1** (5.0×10^{-3} mol/L) with NaNS (7.5×10^{-3} mol/L) in DMSO-d_6/0.5% water

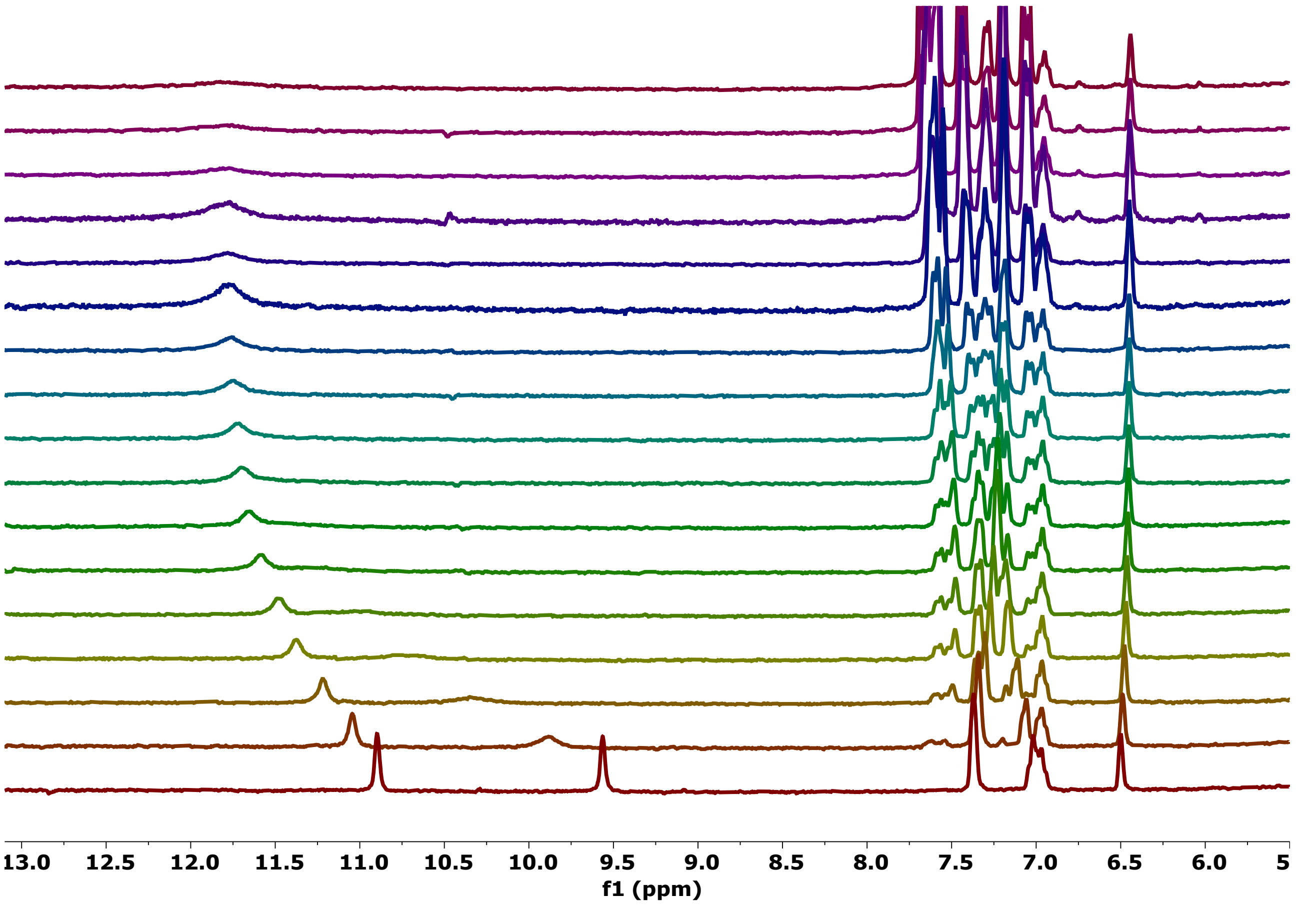
13.0
12.5
12.0
11.5
11.0
10.5
10.0
9.5
9.0
8.5
8.0
7.5
7.0
6.5
6.0
5
f1 (ppm)

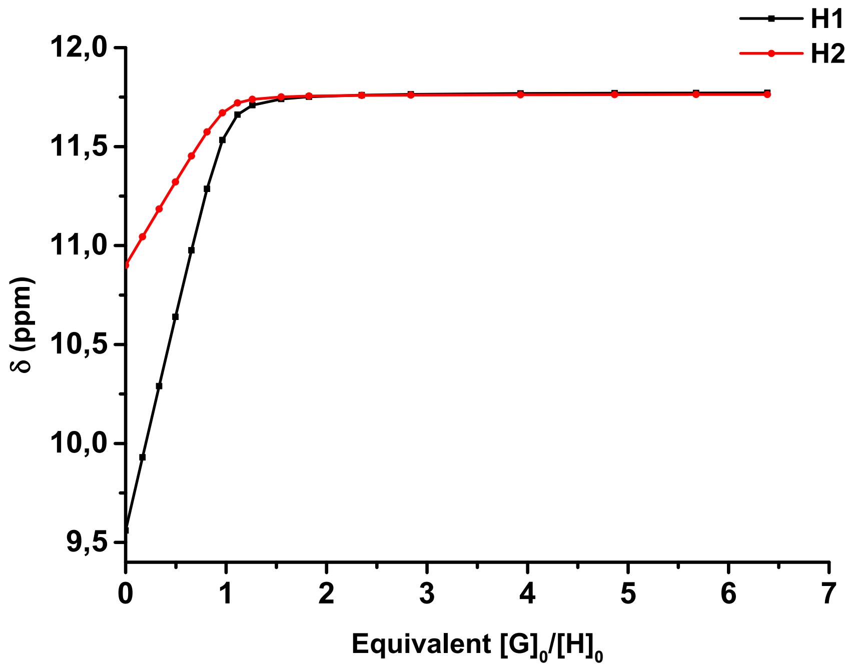
H1
H2
12,0
11,5
11,0
10,5
10,0
9,5
δ (ppm)
0
1
2
3
4
5
6
7
Equivalent [G]0/[H]0

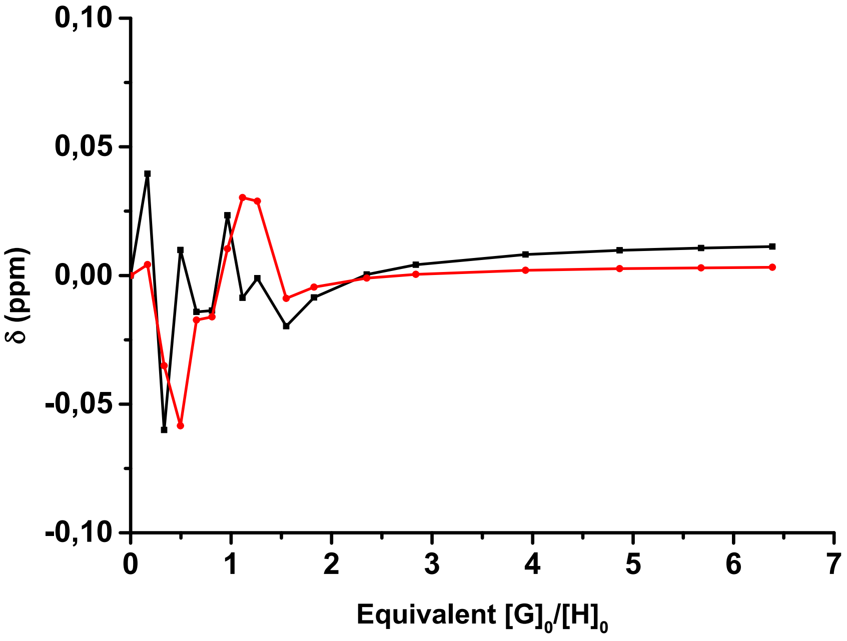

K	K error (%)	SSR	Datapoints fitted	Params fitted	H coeffs	HG coeffs	Raw coeffs 1	Raw coeffs 2
23766.6809	25.817434	0.01436628	34	3	9.56	11.7749013	9.56	11.7749013
					10.9	11.7646264	10.9	11.7646264

http://app.supramolecular.org/bindfit/view/0791d2c4-5d5d-4a67-8d02-d8191adc2599

Figure S10. Stack plot of the ^{1}H NMR titration of **L1** (5.0×10^{-3} mol/L) with NaNS (7.5×10^{-3} mol/L) in DMSO-d_6/10% water

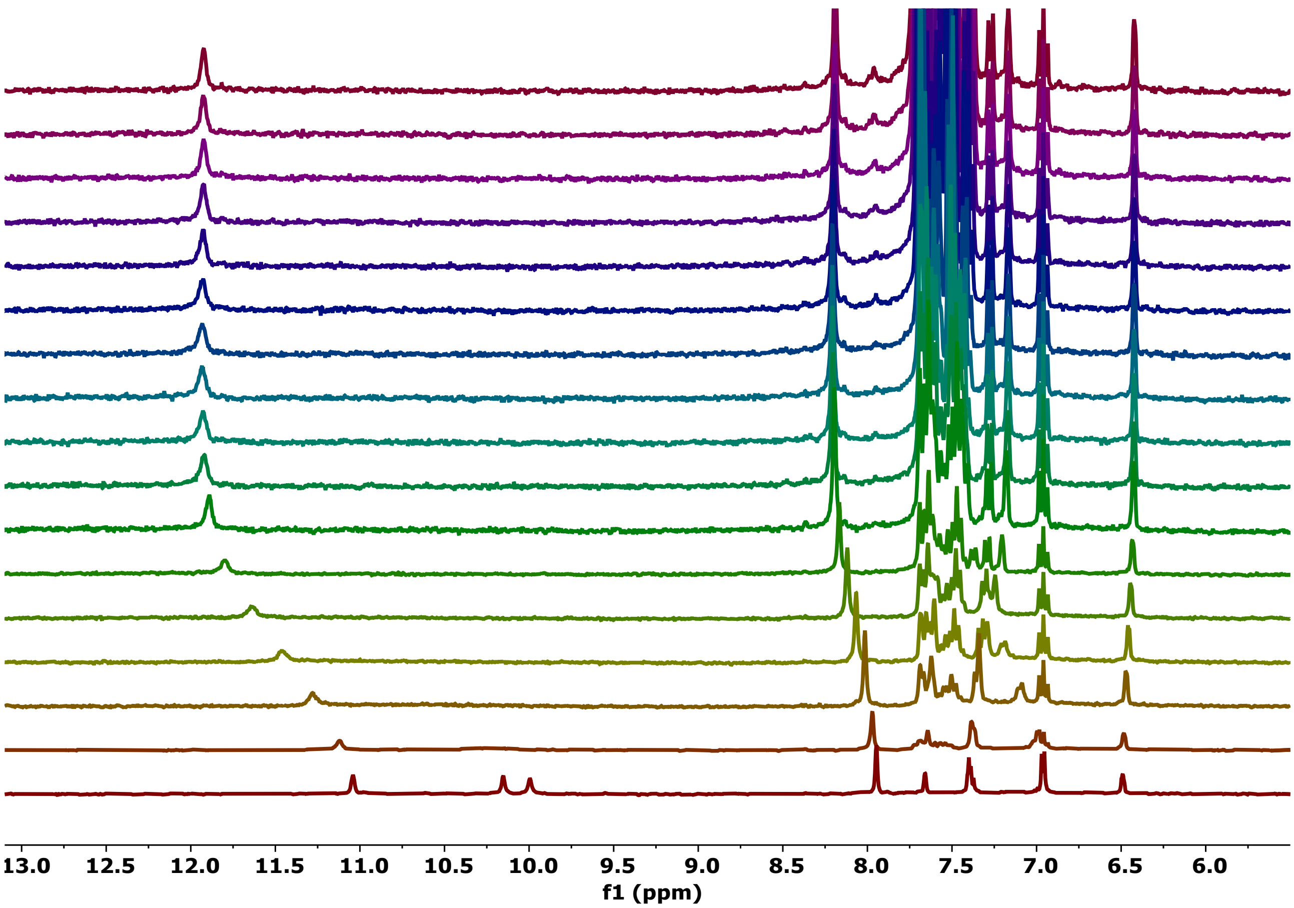

Figure S11. Stack plot of the ^{1}H NMR titration of **L2** (5.0×10^{-3} mol/L) with NaKF (7.5×10^{-3} mol/L) in DMSO-d_6/0.5% water.

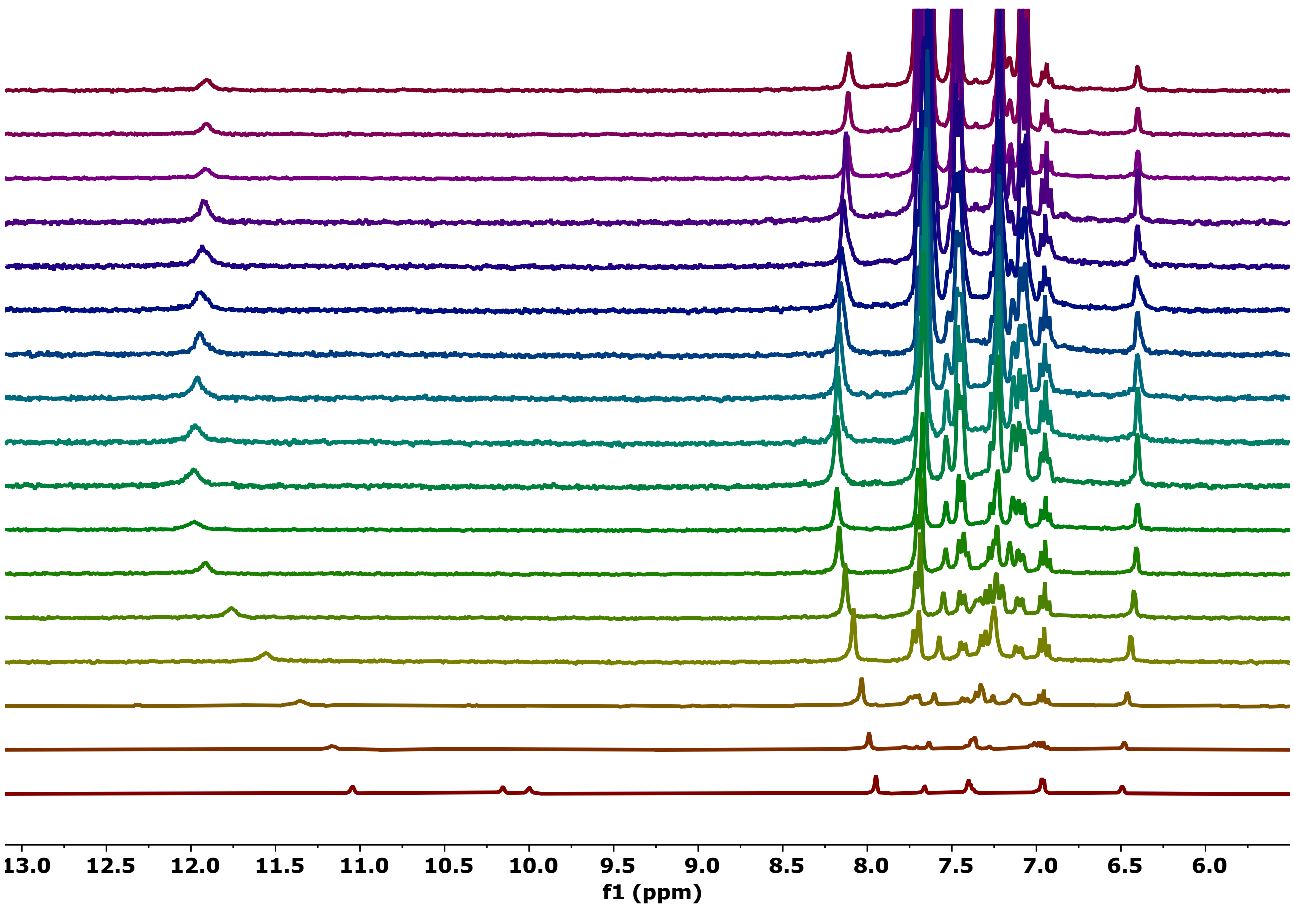

Figure S12. Stack plot of the ^{1}H NMR titration of **L2** (5.0×10^{-3} mol/L) with NaNS (7.5×10^{-3} mol/L) in DMSO-d_6/0.5% water

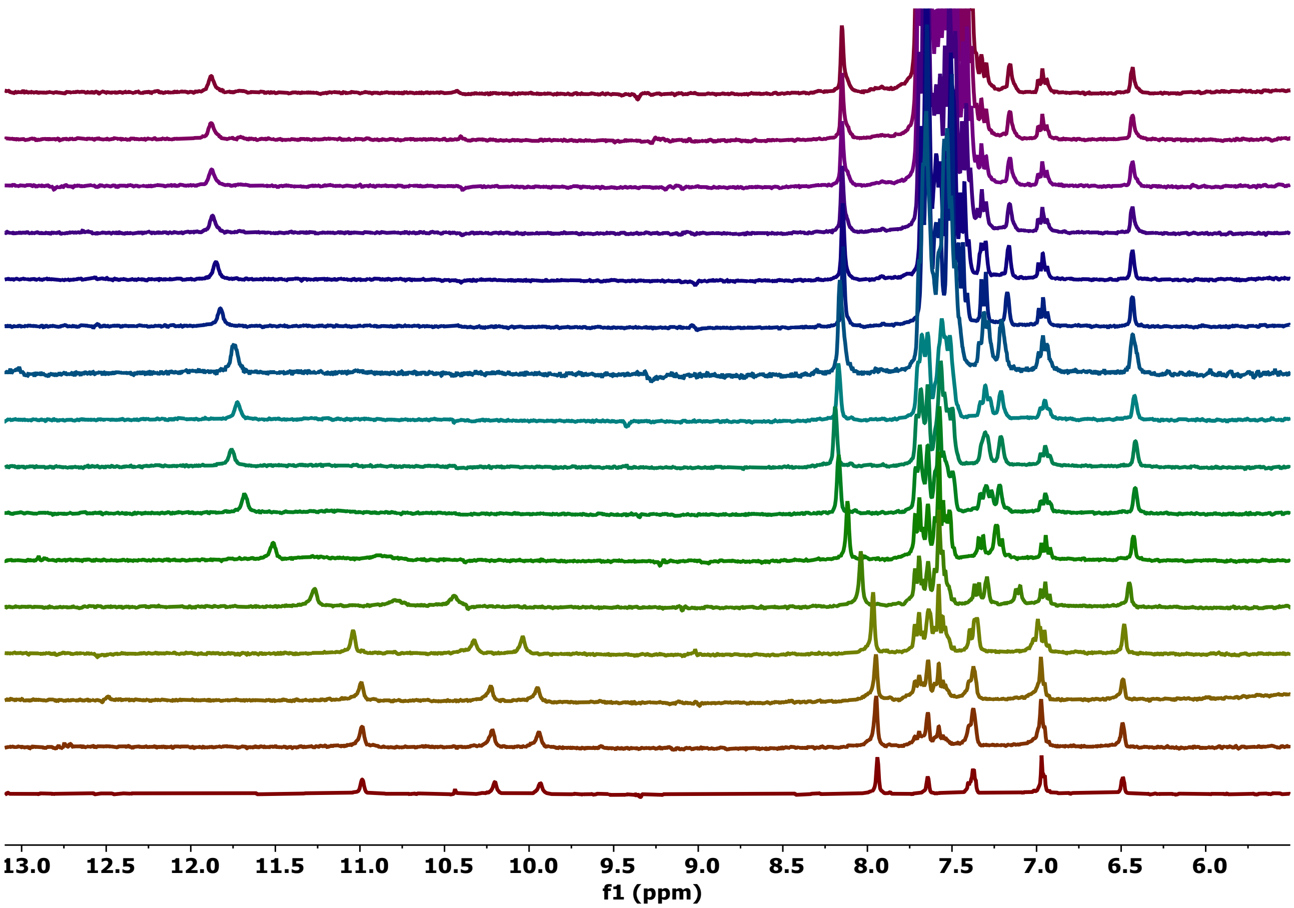

Figure S13. Stack plot of the ^{1}H NMR titration of **L2** (5.0×10^{-3} mol/L) with NaKF (7.5×10^{-3} mol/L) in DMSO-d_6/10% water.

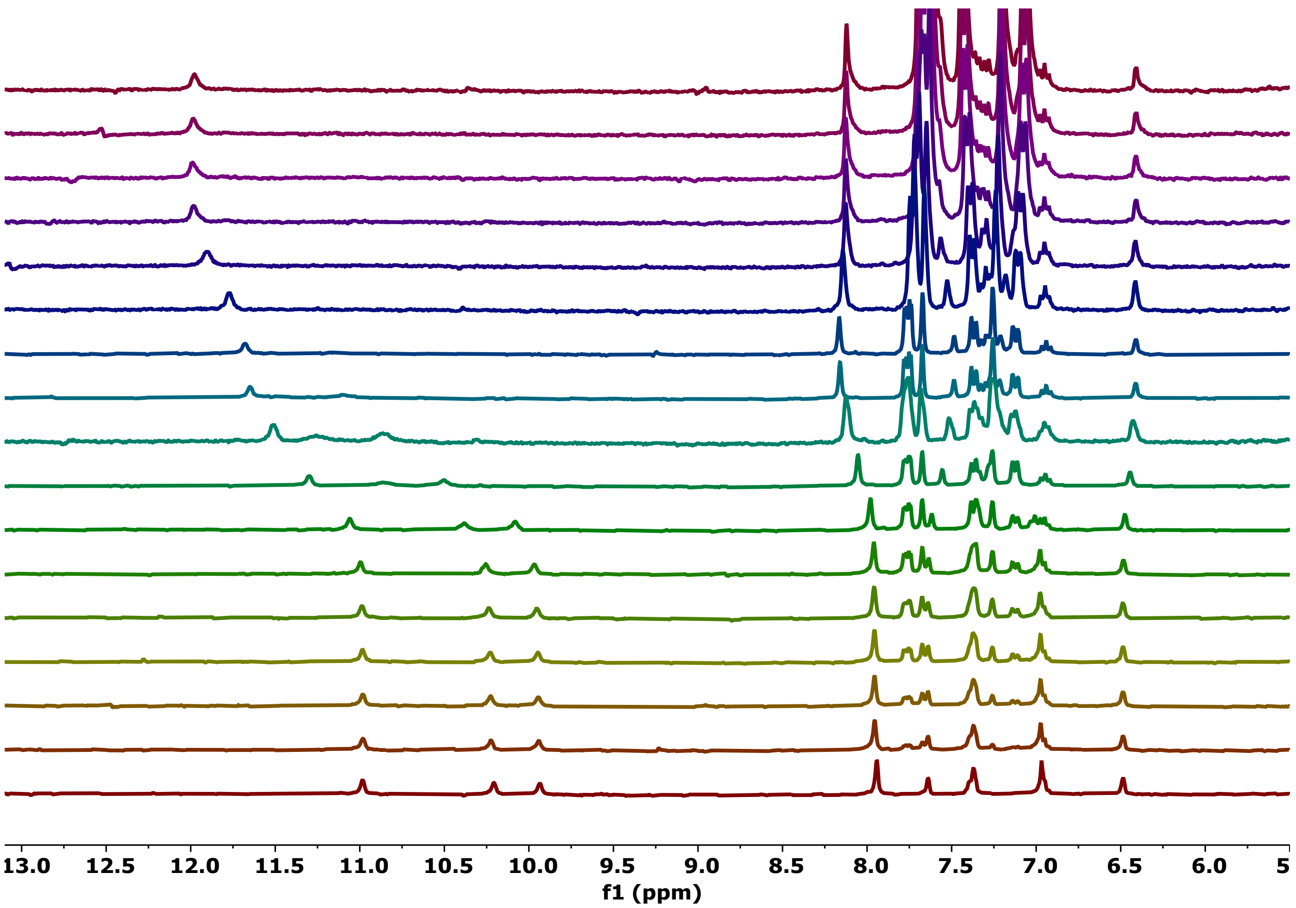

Figure S14. Stack plot of the ^{1}H NMR titration of **L2** (5.0×10^{-3} mol/L) with NaNS (7.5×10^{-3} mol/L) in DMSO-d_6/10% water.

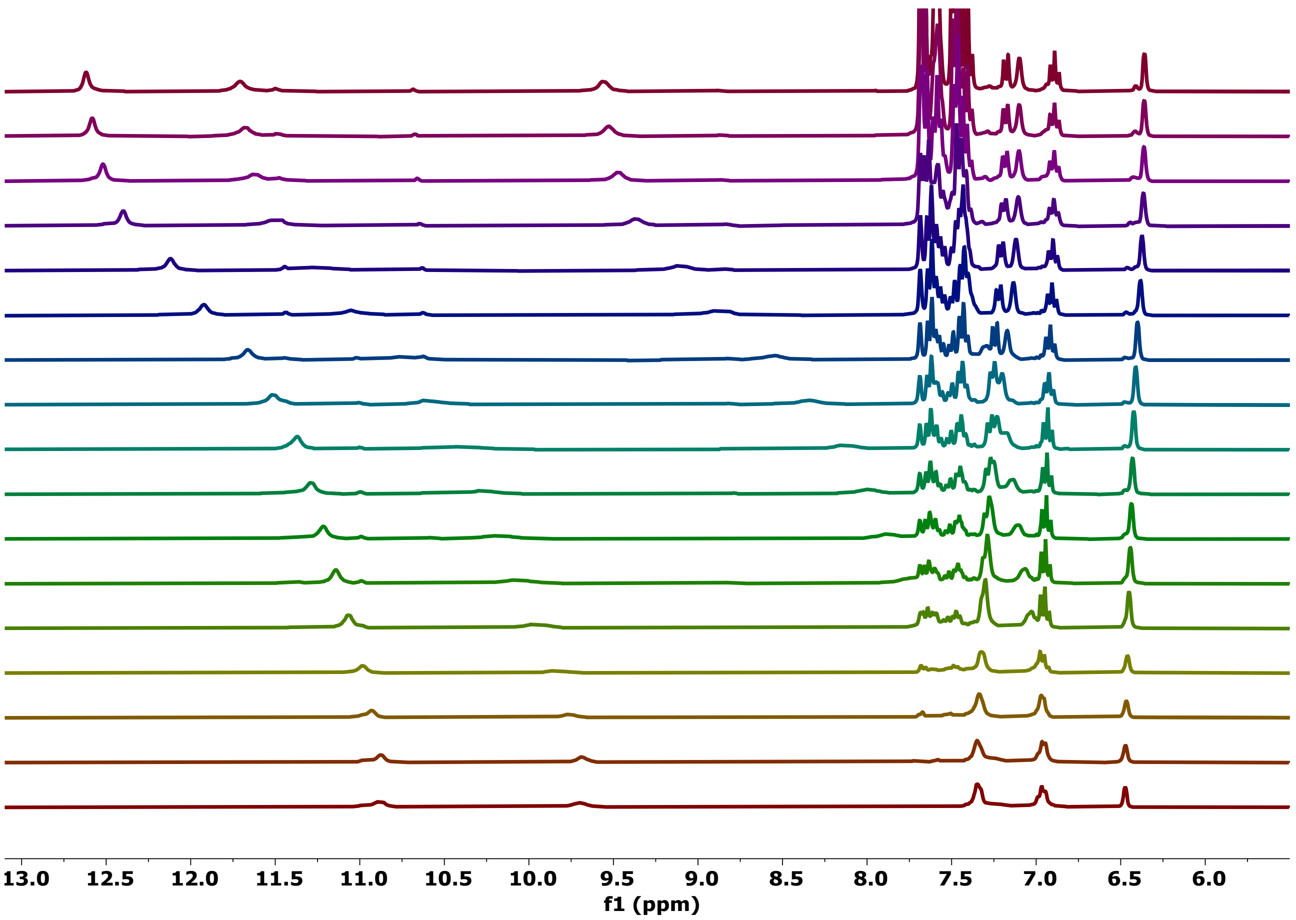
13.0
12.5
12.0
11.5
11.0
10.5
10.0
9.5
9.0
8.5
8.0
7.5
7.0
6.5
6.0
f1 (ppm)

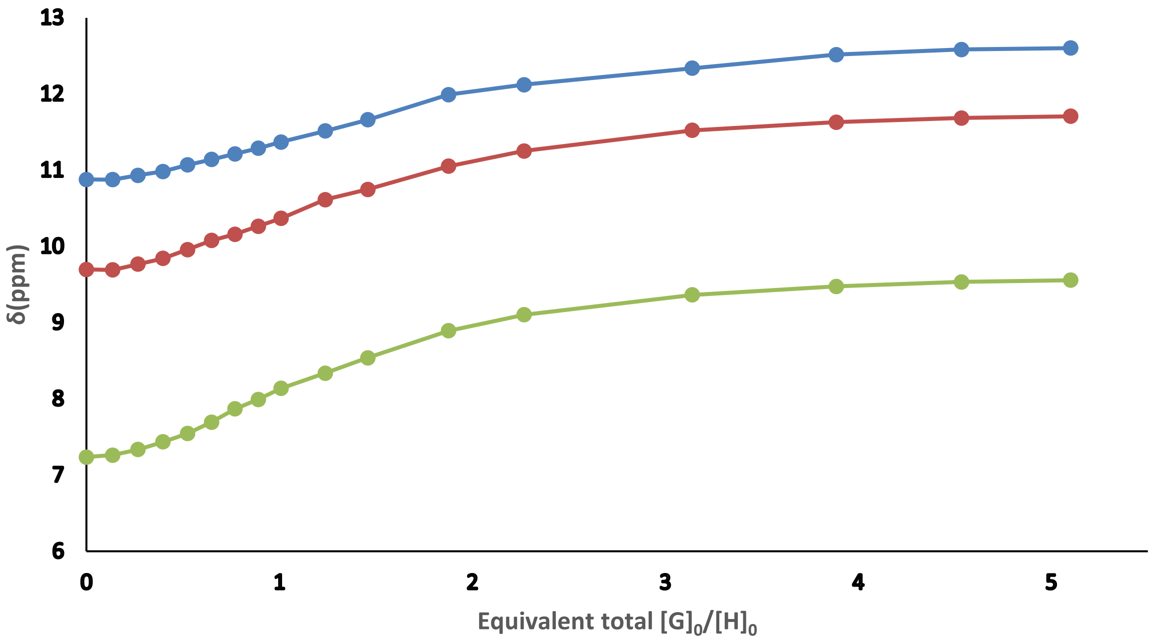
13
12
11
10
9
8
7
6
δ(ppm)
0
1
2
3
4
5
Equivalent total [G]0/[H]0

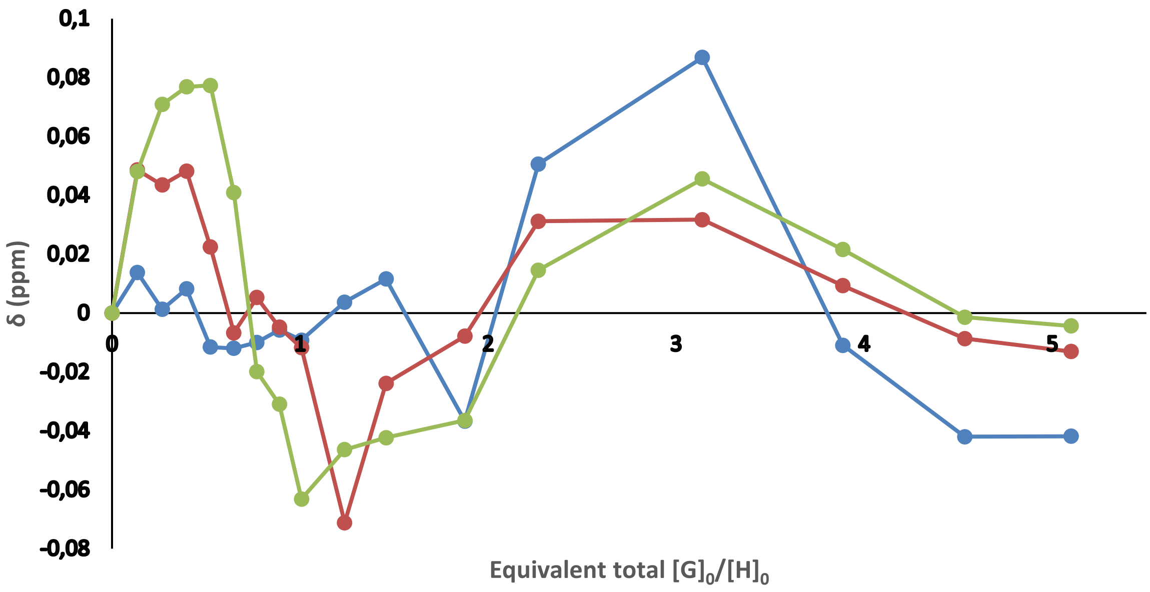

K_{11}	K_{12}	K_{11} error (%)	K_{12} error (%)	SSR	Datapoints fitted	Params fitted	H coeffs	HG coeffs	HG2 coeffs	Raw coeffs 1	Raw coeffs 2	Raw coeffs 3
596,7150599	1352,537096	40,87312408	8,800936831	0,065564306	51	8	10,879	10,68433269	12,64661338	10,879	10,68433269	12,64661338
							9,698	9,836418023	11,78189211	9,698	9,836418023	11,78189211
							7,236	7,714441436	9,641069889	7,236	7,714441436	9,641069889

http://app.supramolecular.org/bindfit/view/67e80016-f619-4aee-b480-ccee0463c0b6

Figure S15. ^{1}H NMR titration of **L4** (5.0×10^{-3} mol/L) with NaKF (7.5×10^{-3} mol/L) in DMSO-d_6/0.5% water

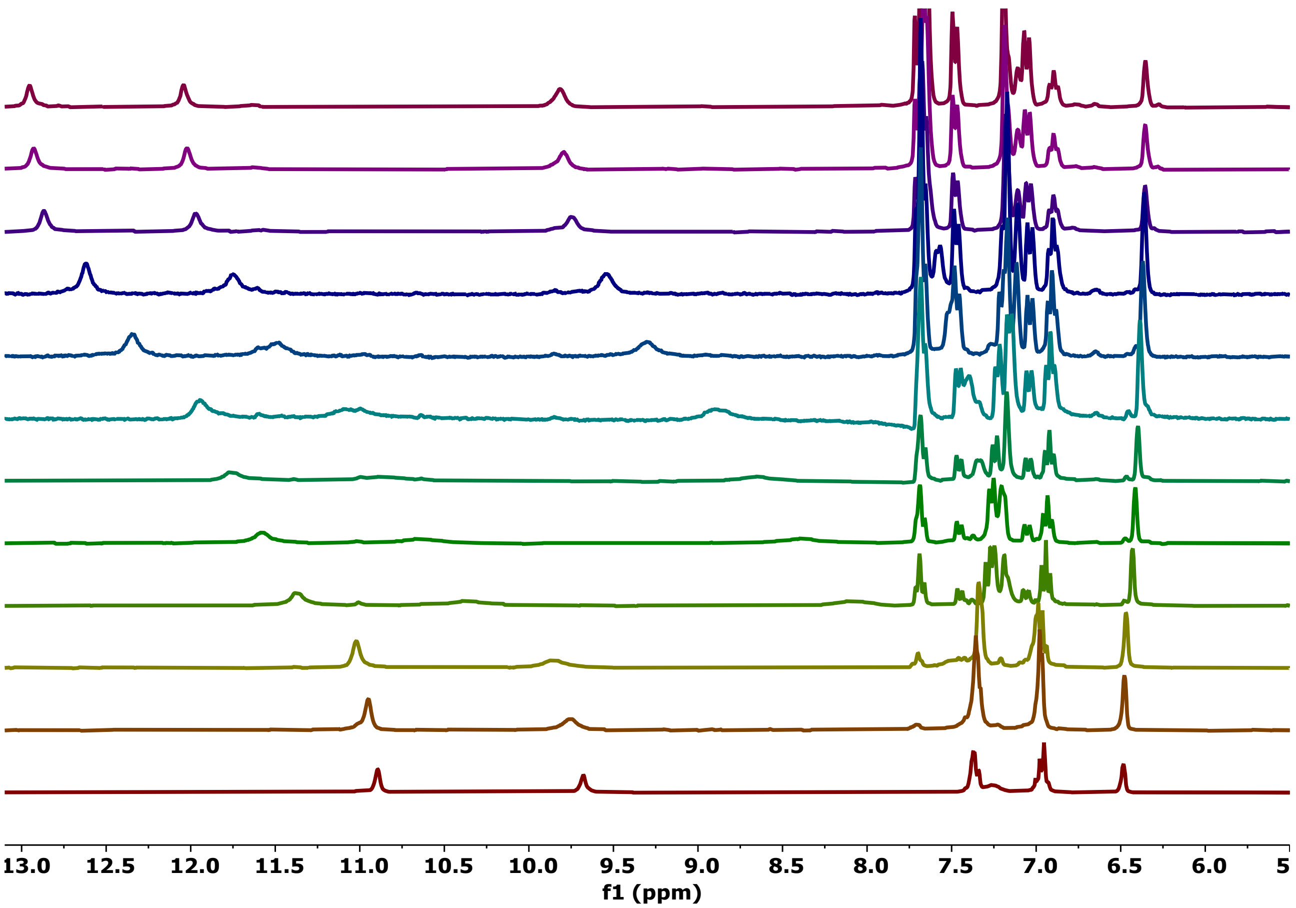
13.0
12.5
12.0
11.5
11.0
10.5
10.0
9.5
9.0
8.5
8.0
7.5
7.0
6.5
6.0
5
f1 (ppm)

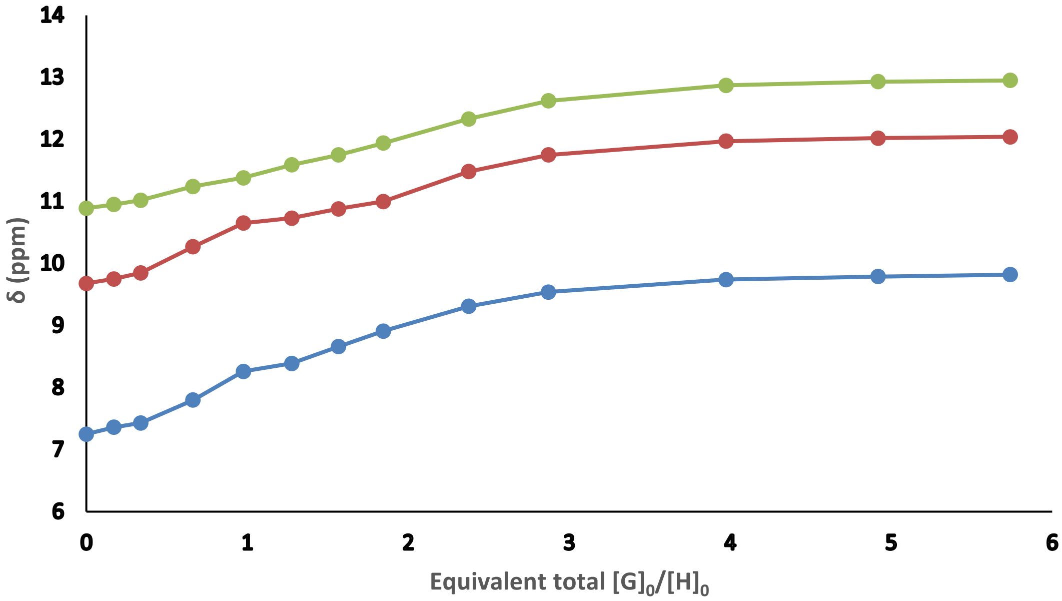
δ (ppm)
14
13
12
11
10
9
8
7
6
0
1
2
3
4
5
6
Equivalent total $[G]_0/[H]_0$

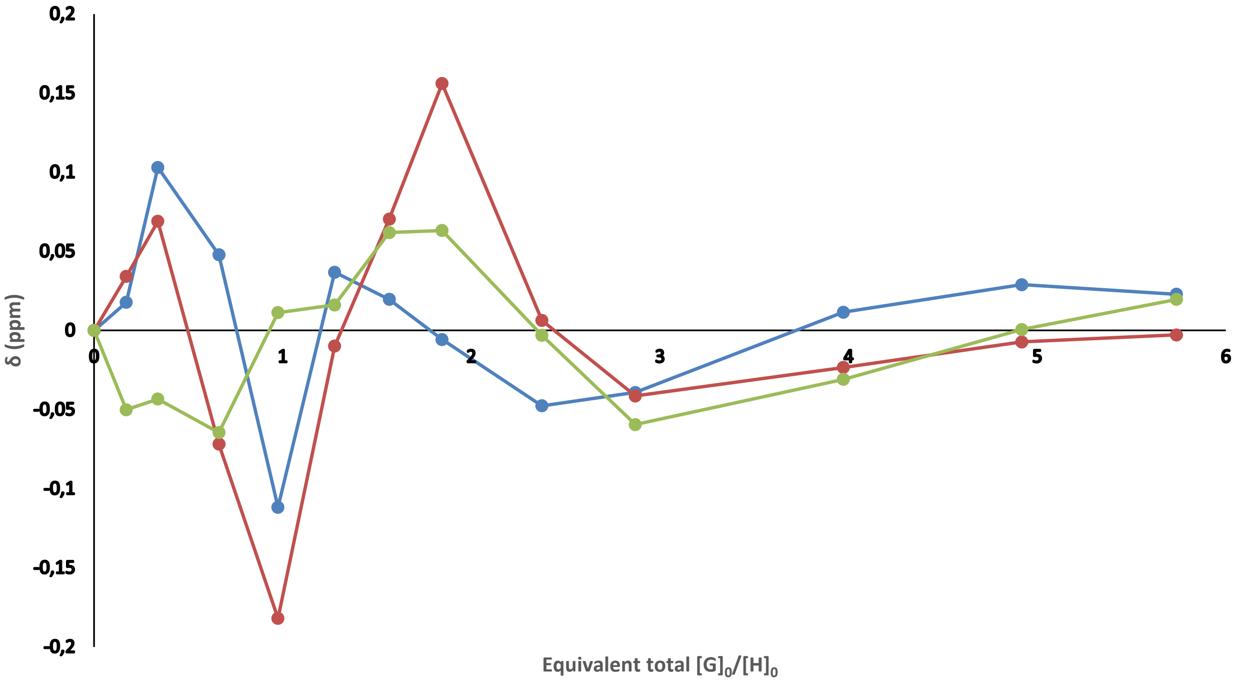

K_{11}	K_{12}	K_{11} error (%)	K_{12} error (%)	SSR	Datapoints fitted	Params fitted	H coeffs	HG coeffs	HG2 coeffs	Raw coeffs 1	Raw coeffs 2	Raw coeffs 3
0,809552	162510,1231	24,83348461	25,25836281	0,130375122	39	8	7,25	105,498879	9,866589089	7,25	105,498879	9,866589089
							9,68	68,18359992	12,06871168	9,68	68,18359992	12,06871168
							10,89	-146,6070637	13,06525425	10,89	-146,6070637	13,06525425

http://app.supramolecular.org/bindfit/view/cd864110-2354-41b9-ab58-1517748b14c6 (1)

http://app.supramolecular.org/bindfit/view/c0da35a2-4182-4b83-89ef-76236b1c62a6 (2).da valutare

Figure S16. ^{1}H NMR titration of **L4** (5.0×10^{-3} mol/L) with NaNS (7.5×10^{-3} mol/L) in DMSO-d_6/0.5% water

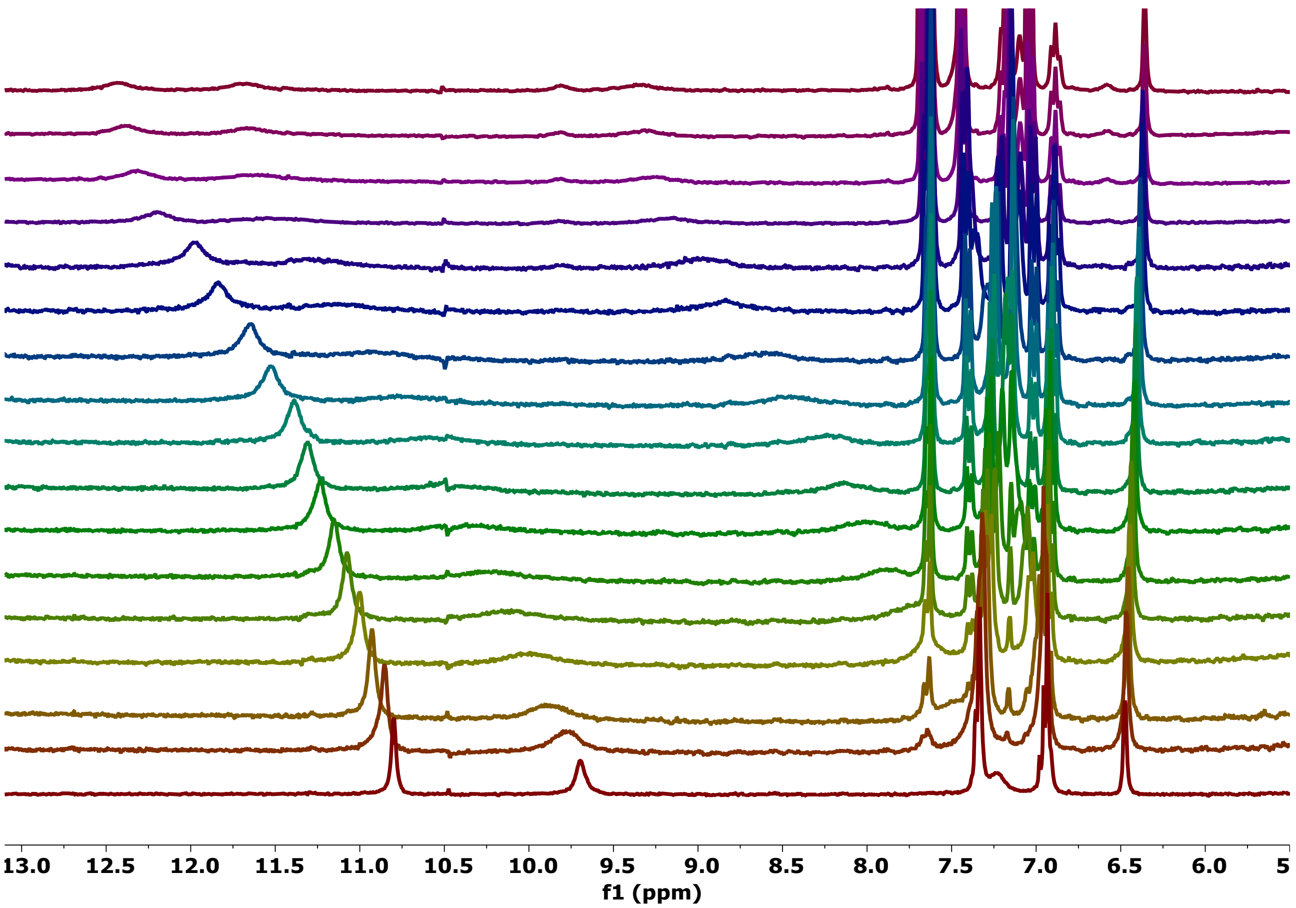
13.0
12.5
12.0
11.5
11.0
10.5
10.0
9.5
9.0
8.5
8.0
7.5
7.0
6.5
6.0
5
f1 (ppm)

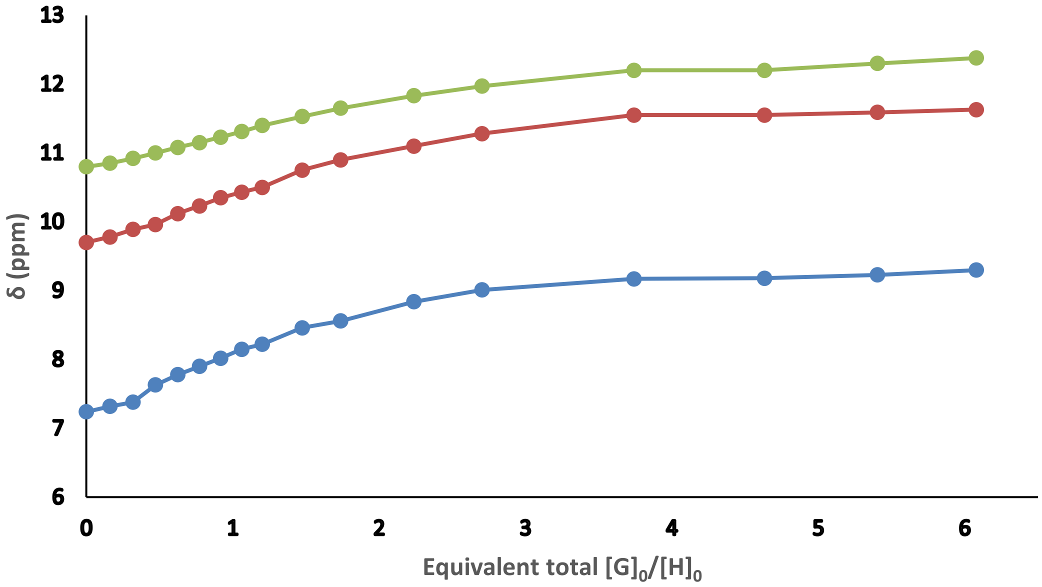
δ (ppm)
13
12
11
10
9
8
7
6
0
1
2
3
4
5
6
Equivalent total $[G]_0/[H]_0$

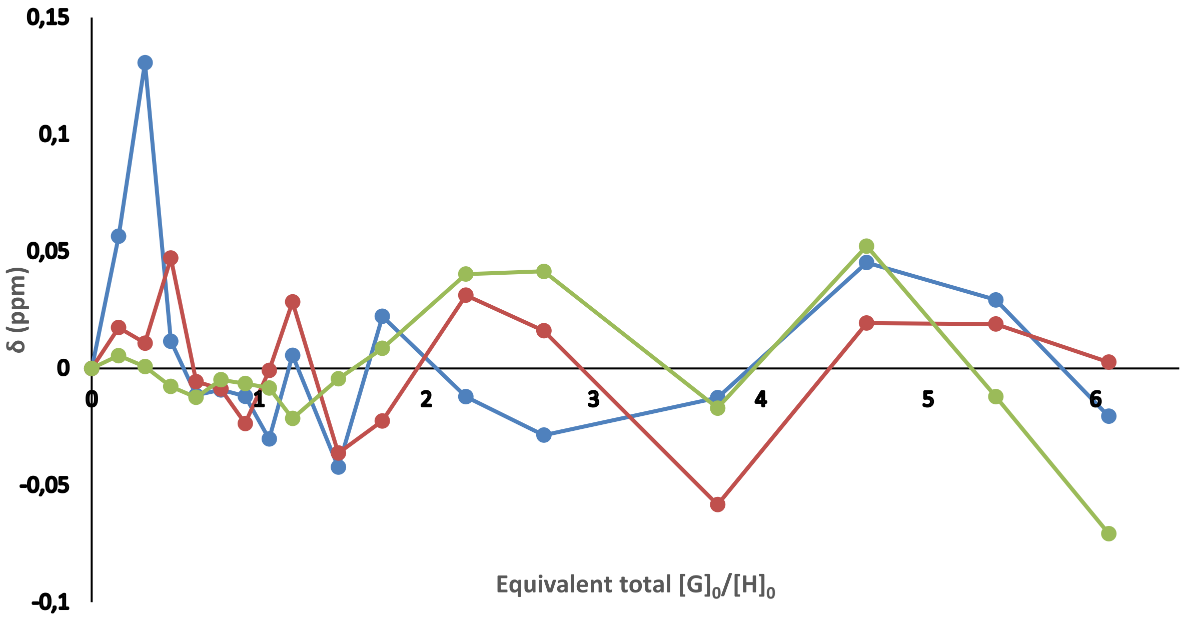

K_{11}	K_{12}	K_{11} error (%)	K_{12} error (%)	SSR	Datapoints fitted	Params fitted	H coeffs	HG coeffs	HG2 coeffs	Raw coeffs 1	Raw coeffs 2	Raw coeffs 3
949,9125128	498,4204574	33,77676113	12,58946063	0,052155713	51	8	7,24	8,268532247	9,389162706	7,24	8,268532247	9,389162706
							9,7	10,39759693	11,76372373	9,7	10,39759693	11,76372373
							10,8	11,16530791	12,42938585	10,8	11,16530791	12,42938585

http://app.supramolecular.org/bindfit/view/a6beac4a-9f25-45dd-a7bd-308771670ab6

Figure S17 ^{1}H NMR titration of **L4** (5.0 × 10^{-3} mol/L) with NaNS (7.5 × 10^{-3} mol/L) in DMSO-d_6/10% water

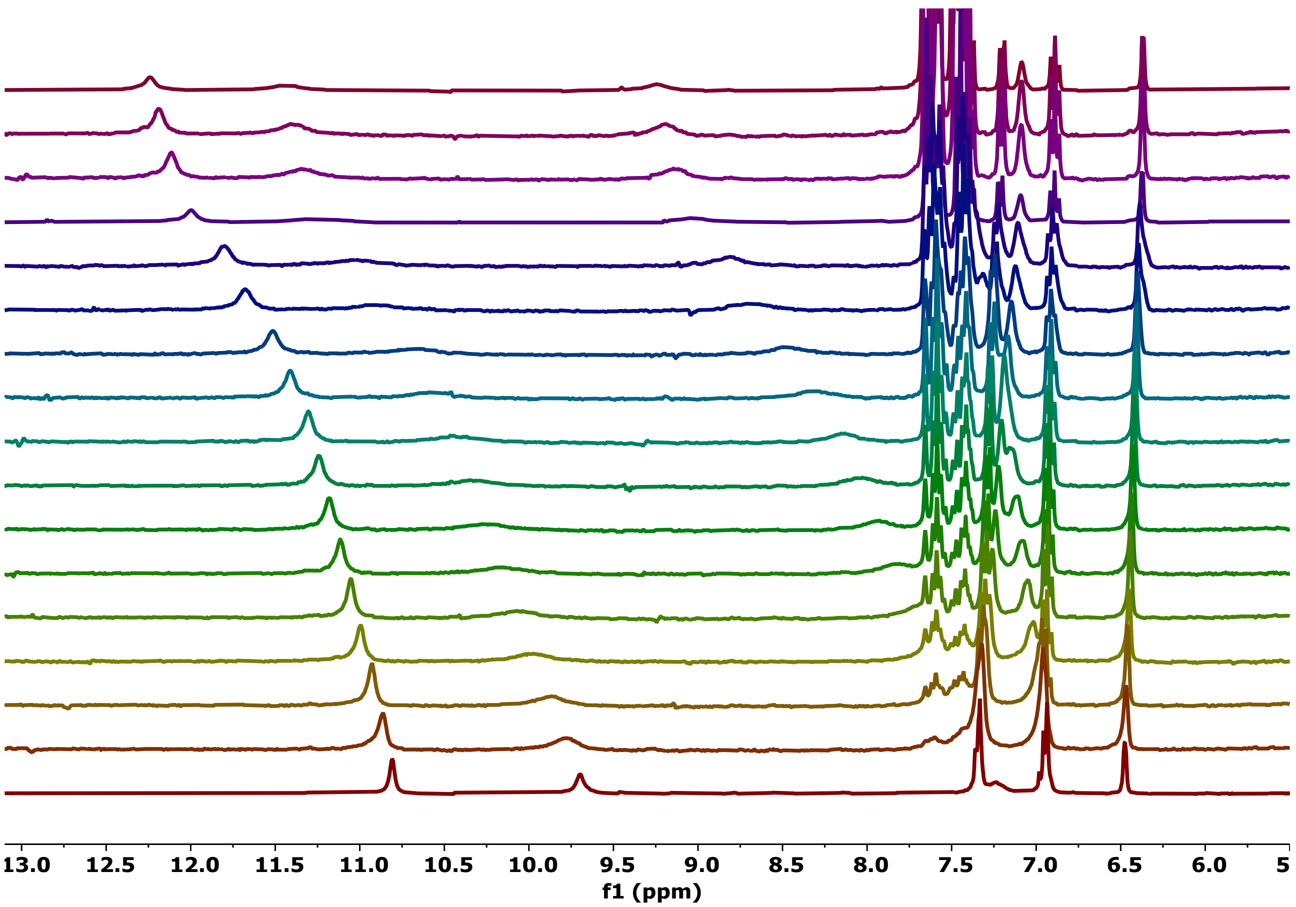
13.0
12.5
12.0
11.5
11.0
10.5
10.0
9.5
9.0
8.5
8.0
7.5
7.0
6.5
6.0
5
f1 (ppm)

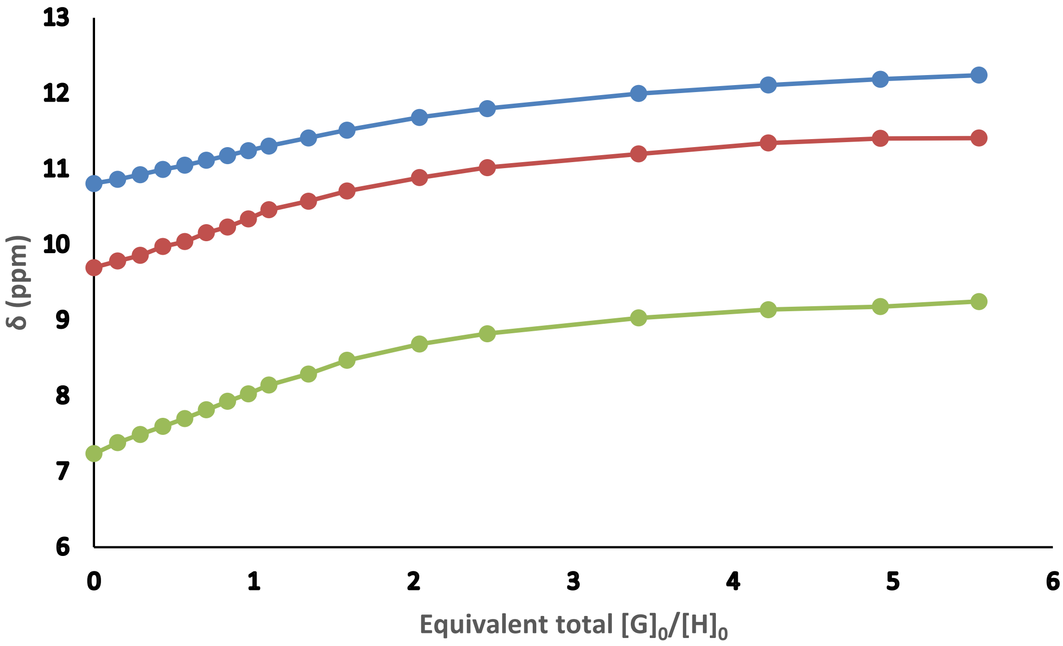
δ (ppm)
13
12
11
10
9
8
7
6
0
1
2
3
4
5
6
Equivalent total [G]0/[H]0

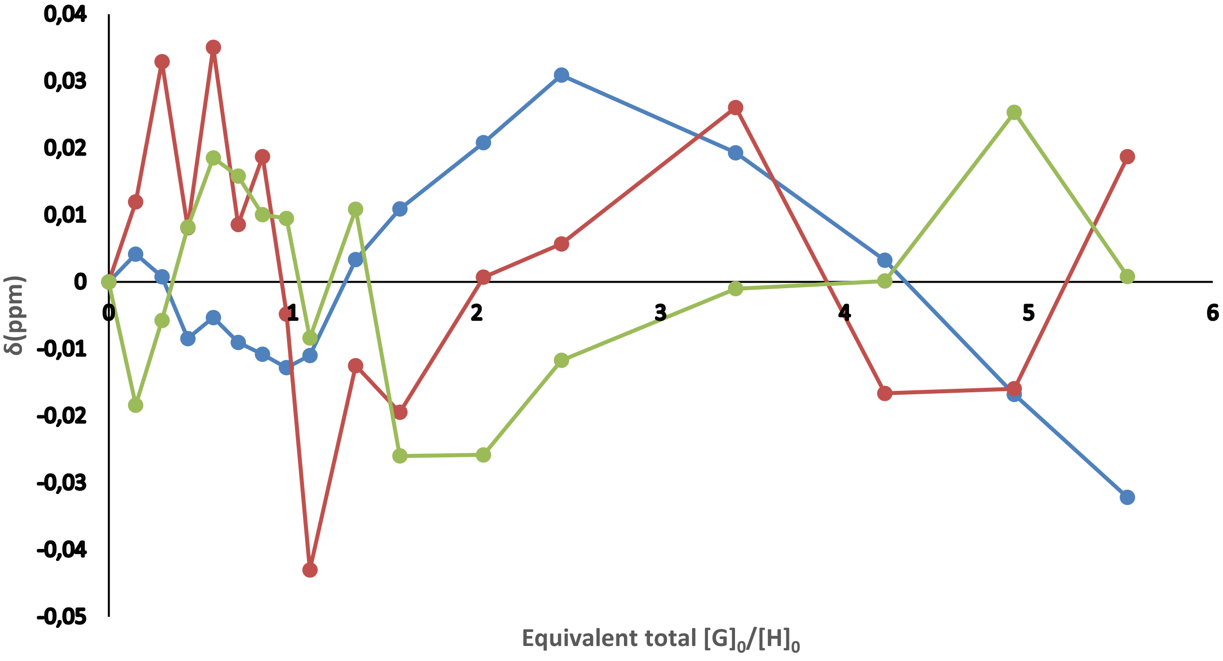

K_{11}	K_{12}	K_{11} error (%)	K_{12} error (%)	SSR	Datapoints fitted	Params fitted	H coeffs	HG coeffs	HG2 coeffs	Raw coeffs 1	Raw coeffs 2	Raw coeffs 3
4923,47356	199,052884	37,193245	4,18432206	0,01429811	51	8	10,8076	11,2254962	12,4675978	10,8076	11,2254962	12,4675978
							9,6967	10,3896352	11,7027759	9,6967	10,3896352	11,7027759
							7,2376	8,1310093	9,54558311	7,2376	8,1310093	9,54558311

http://app.supramolecular.org/bindfit/view/06360615-fdc6-40ee-8852-1d5bc0649d70

Figure S18. ^{1}H NMR titration of **L4** (5.0×10^{-3} mol/L) with NaKF (7.5×10^{-3} mol/L) in DMSO-d_6/10% water

3. Potentiometric testing

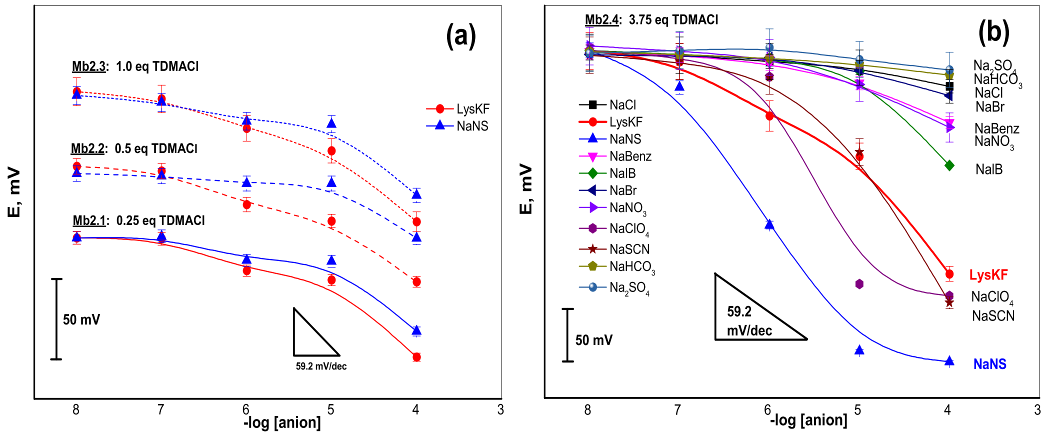

Figure S19. Potentiometric calibration curves of (a) membranes Mb2.1 – Mb2.3 doped with **L2** and 0.25, 0.5 and 1.0 eq of TDMACl anion-exchanger respectively in individual solutions of KF^-, NS^- ions in a concentration rage 1.0×10^{-7}–1.0×10^{-4} mol/L and (b) of membrane Mb2.4 in individual solutions of KF^-, NS^-, and various interfering ions. Plots shows error bars of 4 individual measurements (n=4).

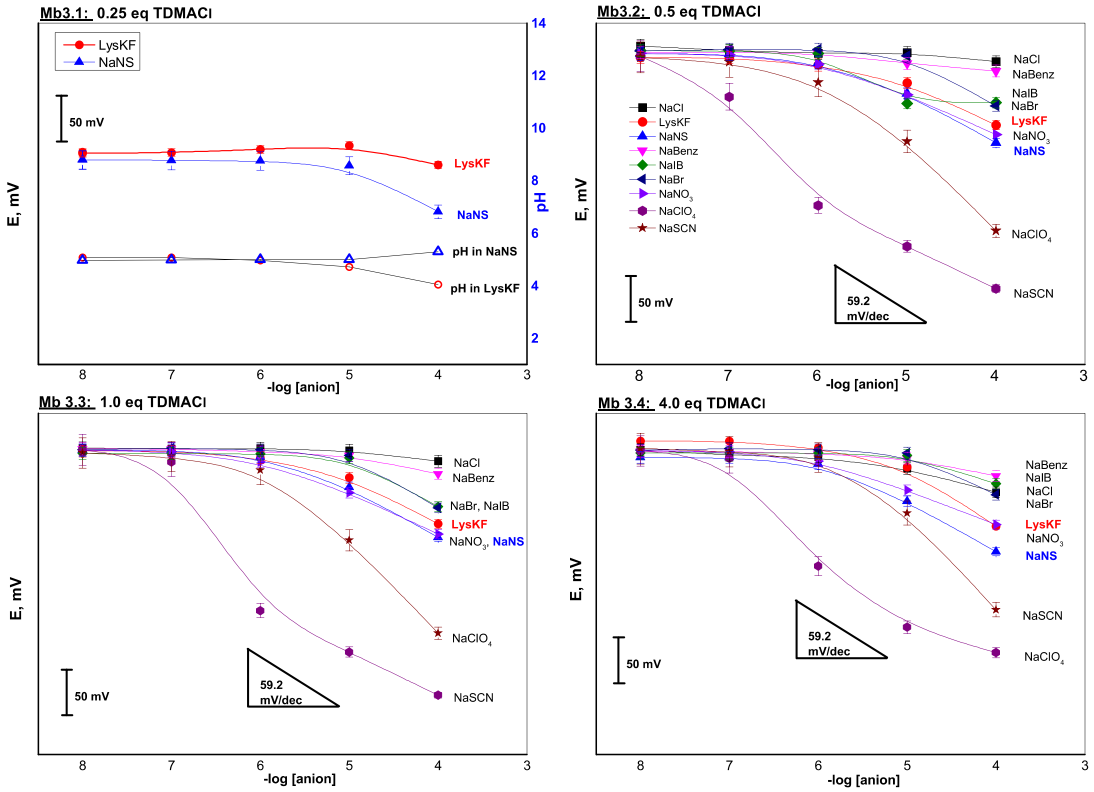

Figure S20. Potentiometric calibration curves of membranes Mb3.1 – Mb3.4 doped with **L3** and 0.25, 0.5, 1.0 and 4.0 eq of TDMACl anion-exchanger respectively in individual solutions of KF^-, NS^- ions and various interfering ions in a concentration rage 1.0×10^{-7}–1.0×10^{-4} mol/L. For Mb3.1 the pH values measured in LysKF and NaNS upon calibration are shown. Plots shows error bars of 4 individual measurements (n=4).

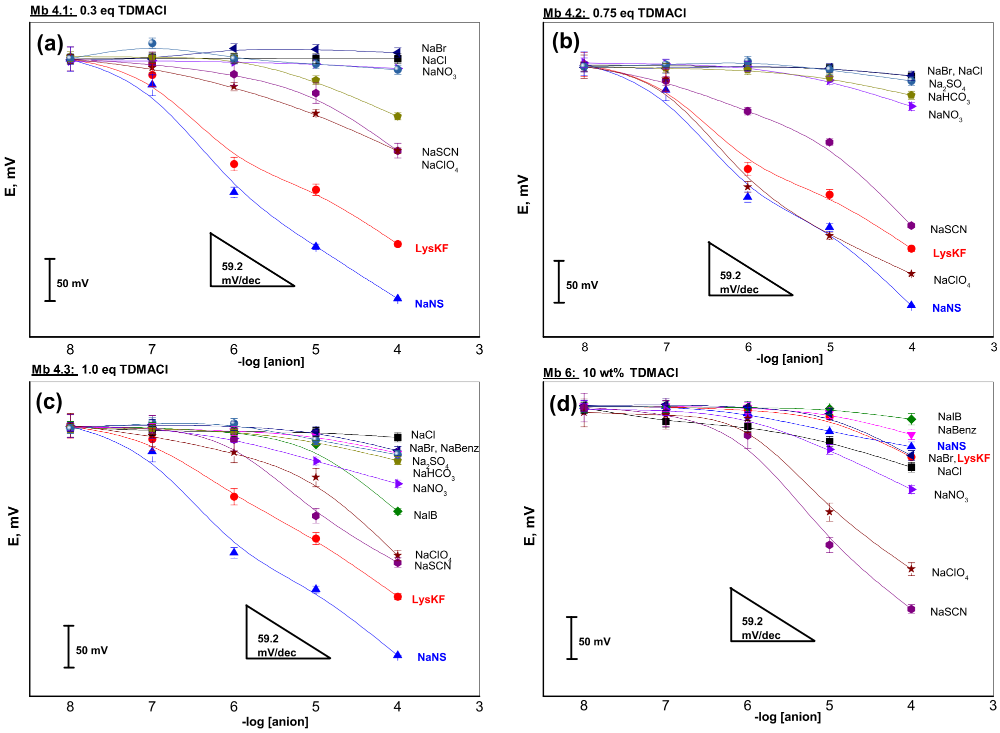

Figure S21. Potentiometric calibration curves of (a), (b), (c) membranes Mb4.1 – Mb4.3 doped with **L4** and 0.3, 0.75, 1.0 eq of TDMACl respectively, and (d) Mb6 prepared with 10wt% of only TDMACl anion-exchanger in individual solutions of KF^-, NS^- and various interfering ions in a concentration rage 1.0×10^{-7}–1.0×10^{-4} mol/L. Plots shows error bars of 4 individual measurements (n = 4).

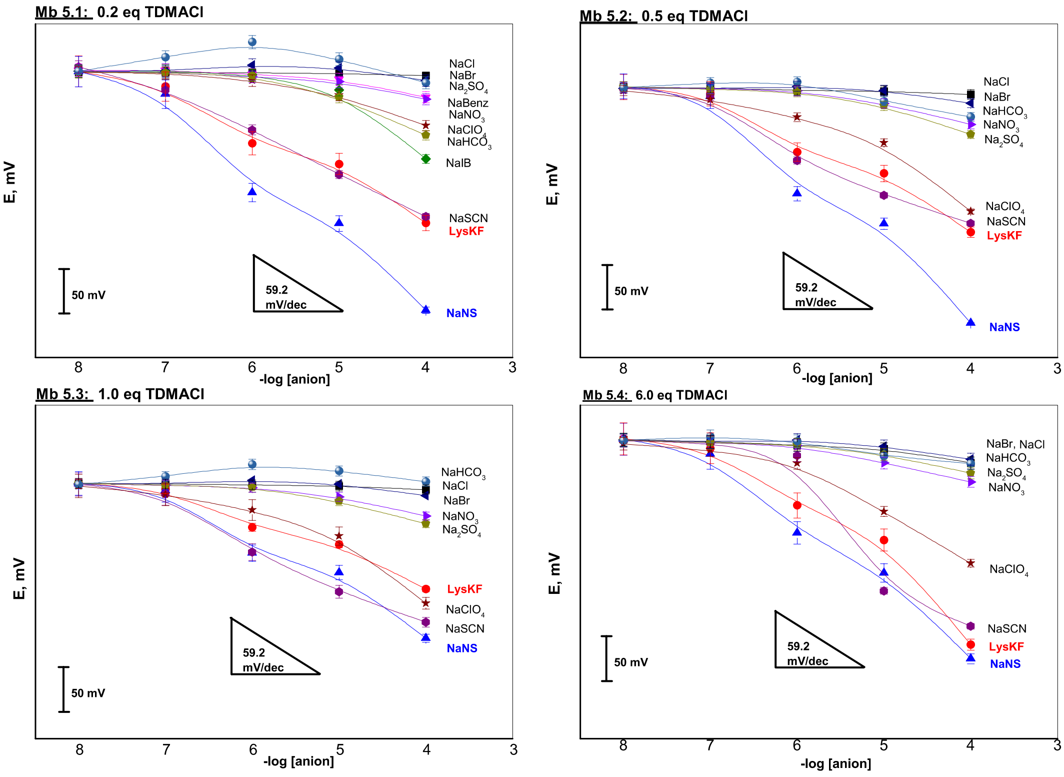

Figure S22. Potentiometric calibration curves of membranes Mb5.1–Mb5.4 doped with **L5** and 0.2, 0.5, 1.0 and 6.0 eq of TDMACl respectively in individual solutions of KF^-, NS^- and various interfering ions in a concentration rage 1.0×10^{-7}–1.0×10^{-4} mol/L. Plots shows error bars of 4 individual measurements (n = 4).

3.1 Super-Nernstian response of L1-doped membranes

The existence of either dimeric squaramide species, or catemeric clusters in solution was reported in literature, and depended on the solvent polarity (the latter were revealed only in low-polarity solvents) [**Error! Bookmark not defined.**]. Hence, for the polymeric membranes plasticized with medium-polar TOP plasticizer investigated in the present work, the possibility of ligand-ligand (**L1-L1**) dimers formation of possible configuration reported in Figure S23, may be hypothesized. In the case of smaller-sized non-linear KF^- ion sensing, the **L1-L1**/anion adducts may first form in the polymeric membrane phase, further the second ligand unit (or **L1-L1** dimer) may laterally approach and additionally coordinate the third (non-carboxylic) O atom in KF^- ion structure via additional hydrogen bonding, thus resulting in 2:1 (or either 4:1) ligand/anion complex stoichiometry. This hypothesis in a large degree is supported by the obtained widest linear response range and close-to Nernstian slopes (–54.4 mV/dec) of membrane mb 1.1, doped with 0.25 eq of TDMACl anion exchanger with respect to the L1 ligand amount. The KF^- sensitivity remains the same at low analyte concentrations also for membrane mb 1.2 doped (see Fig. 3b) with 0.5 eq of TDMACl, the mixed stoichiometry complexes may still present in the membrane, but 1:1 ionophore/anion adducts formation is prevalent in

membrane mb 1.3 and this ratio did not change for mb 1.4 doped with double excess of TDMACl (2 eq.) over the **L1** amount.

For NS⁻ ions the 1:1 **L1**/anion adducts formation through the hydrogen bonding between squaramide NH-groups and two oxygen atoms coming from carboxylic group of NS⁻ is prevalent in membrane mb 1.3, for which the slightly higher then Nernstian (–63.8 mV/dec) slope was registered in all the tested concentration ranges. We attribute the super-Nernstian naproxen sensitivities of membranes mb 1.1 and mb 1.2, to the initial binding of anion to **L1**-**L1** dimers present in membrane, followed by the dissociation of the **L1**-**L1** adduct**,** resulting in the release of free **L1** non-dimerized units readily participating in additional NS⁻ ions coordination. Since in comparison to KF⁻, NS⁻ ion has an almost linear structure and bigger size (length), the third (non-carboxylic) O atom in NS⁻ can be hardly coordinated by another **L1** unit due to the sterically difficulties for squaramide NH groups to approach it. The cooperativity of indole group NH is very probable decreased in this case due to the π-π repulsions from aromatic rings of NS⁻ and **L1** indoles by themselves. All these factors bring to the best potentiometric sensitivity of membrane mb 1.3 to NS⁻, while the following increase of TDMACl amount destabilizes the membrane response due to possible concurrent coordination of Cl⁻ anions from anion-exchanger and NS⁻ target ions on **L1** and the multi-ion complexes formation of complex stoichiometry in membrane mb 1.4.

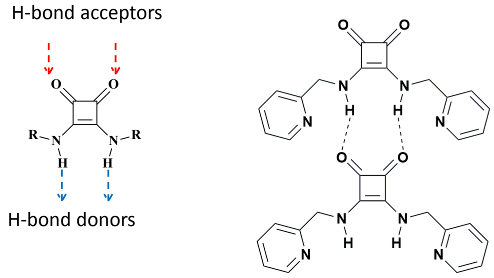

(a) **(b)**

Figure S23. (a) (H-bond acceptor and donor sites in squaramide moiety; (b) the schematic presentation of potential dimers of bis-secondary squaramides, reported from [1].

3.2 Phase-boundary model calculations

The calculated values of $[L]$, $[I^-]$, $[IL^-]$, $[I_2L^{2-}]$ and $\log K^{pot}_{I/J}$ for **L1**- and **L4**-based membranes are shown in Figures S24 and S25 respectively.

To calculate $\log K^{pot}_{I/J}$, the system of equations (3) has been solved as following:

From $\beta_{IL} = [IL^-]/([I^-][L])$ $[IL^-]$ can be expressed as: $[IL^-] = \beta_{IL}[I^-][L]$ (4)

From $\beta_{I_2L} = [I_2L^{2-}]/([I^-]^2[L])$ $[I_2L^{2-}]$ can be expressed as: $[I_2L^{2-}] = \beta_{I_2L}[I^-]^2[L]$ (5)

Therefore $[L_t] = [L] + [IL^-] + [I_2L^{2-}]$ can be rewritten as

$$[L_t] = [L] + \beta_{IL}[I^-][L] + \beta_{I_2L}[I^-]^2[L] = [L](1 + \beta_{IL}[I^-] + \beta_{I_2L}[I^-]^2) \quad (6)$$

Then [L] can be expressed as following: $[L] = [L_t]/(1 + \beta_{IL}[I^-] + \beta_{I_2L}[I^-]^2)$ (7)

and $[R^+] = [I^-] + [IL^-] + 2[I_2L^{2-}]$ can be expressed as following: $[R^+] = [I^-] + \beta_{IL}[I^-][L] + 2\beta_{I_2L}[I^-]^2[L]$ (8)

Combining equation (7) in equation (8) and reorganizing, the cubic equation for $[I^-]$can be obtained as following:

$$\beta_{I_2L}[I^-]^3 + [I^-]^2(\beta_{IL} + 2\beta_{I_2L}[L_t] - \beta_{I_2L}[R^+]) + [I^-](1 + \beta_{IL}[L_t] - \beta_{IL}[R^+]) = 0 \quad (9a)$$

The equation (9) can be modified as:

$$[I^-]\{\beta_{I_2L}[I^-]^2 + [I^-](\beta_{IL} + 2\beta_{I_2L}[L_t] - \beta_{I_2L}[R^+]) + (1 + \beta_{IL}[L_t] - \beta_{IL}[R^+])\} = 0 \quad (9b)$$

The cubic equation (9b) can be solved: one solution is $[I^-]_1 = 0$; the other two$[I^-]_{2,3}$ – are the solutions of the second order equation in brackets. From the acceptable solution of $[I^-]$, $[L]$ can be calculated from eq. 7. considering the values of β_{IL}, β_{I_2L} determined experimentally, and fixing the values of $[Lt]$ and $[R+]/[Lt]$ (the latter was experimentally varied as: 0.25, 0.5, 1, 2 for ligand **L1** and 0.25, 0.75 and 1 for **L4**).

It is important to specify here that for β_{IL} we have considered: $\beta_{IL} = K_{11}$ (determined by ^{1}H-NMR titrations).

On the other hand, taking into account that K_{12} determined by ^{1}H-NMR titrations is referred to the equilibrium

$$LI^- + I^- = LI_2^{2-} \quad (10)$$

for β_{I_2L} we have considered:

$$\beta_{I_2L} = K_{11}\, K_{12} \quad (11)$$

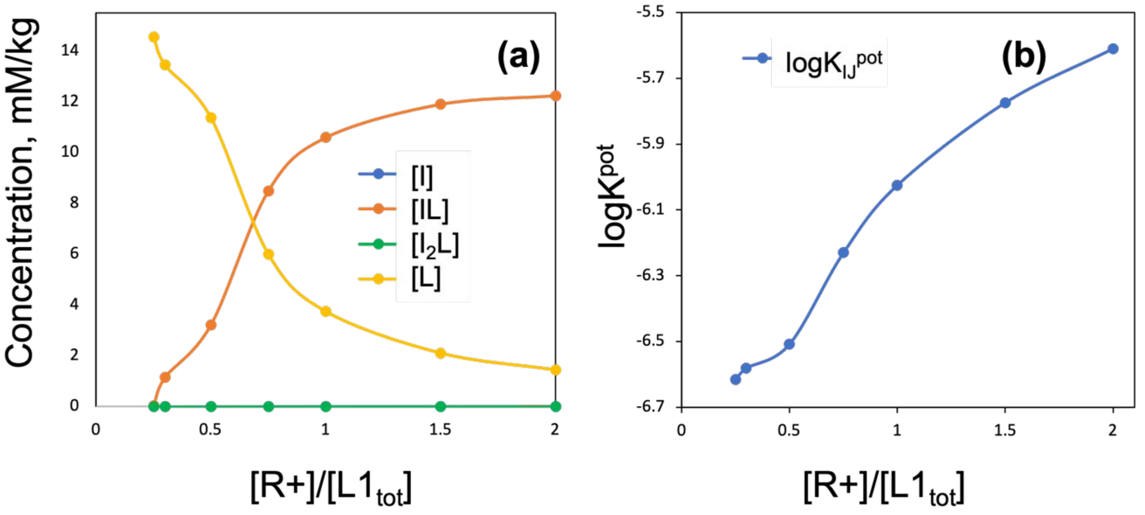

Figure S24. Calculated (a) concentrations of species inside the membrane against lipophilic additive-to-ionophore ratio,[R^+]/[L_t], and (b) selectivity, $\log K_{I/J}^{pot}$ of **L1**-based membranes for an ion I^- that forms 1:1 and 1:2 complexes with the ionophore, **L1**, with respect to an interfering ion J^- that does not bind to **L1**. Shown are calculated values for [**L1**$_t$] = 14.6 mM/kg, K_{11} = 2.38 × 10^4 mol^{-1}/L^{-1} and K_{12} = 2.50 × 10^2 mol^{-1}/L^{-1}, $K_{I/J}^{ex}$ = 0.1.

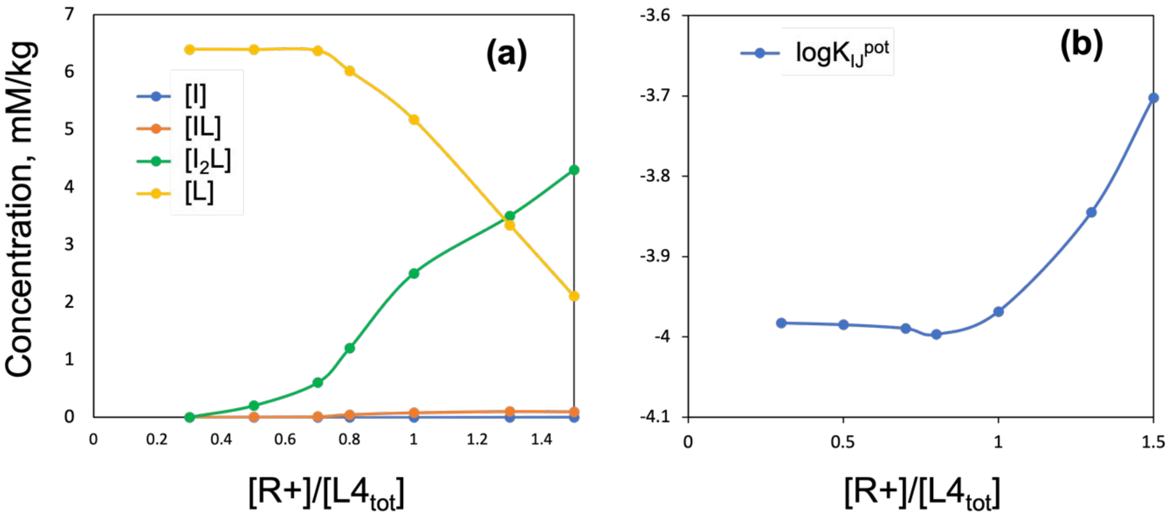

Figure S25. Calculated (a) concentrations of species inside the membrane against lipophilic additive-to-ionophore ratio,[R^+]/[L_t], and (b) selectivity, $\log K_{I/J}^{pot}$of **L4**-based membranes for an ion I^- that forms 1:1 and 1:2 complexes with the ionophore, **L4**, with respect to an interfering ion J^- that does not bind to **L4**.Shown are calculated values for [**L4**$_t$] = 6.4 mM/kg, K_{11} = 1.55 × 10^3 mol^{-1}/L^{-1} and K_{12} = 1.475 × 10^5 mol^{-1}/L^{-1}, $K_{I/J}^{ex}$ = 0.1.

Table S2. Selectivity coefficients of different membrane composition against various interfering ions of membranes based on **L1−L5** ligands with varied amount of TDMACl anion exchanger (compositions listed in Table 1) for NS⁻ as primary ion. Data reported graphically in Figure 4.

	Cl^-	KF^-	$Benz^-$	IB^-	Br^-	NO_3^-	ClO_4^-	SCN-	CO_3^{2-}	SO_4^{2-}
**mb 1.1**	-5.1	-1.7	-3.1	-1.8	-5.1	-4.7	-2.2	-2.8	-6.8	-6.9
**mb 1.2**	-4.7	-1.8	-3.2	-1.8	-4.7	-4.6	-2.4	-3.0	-6.3	-6.9
**mb 1.3**	-4.15	-1.6	-3.1	-1.7	-3.9	-3.6	-0.7	-1.6	-6.0	-5.7
**mb 1.4**	-4.9	-2.5	-3.3	-2.0	-5.0	-4.1	-0.5	-2.3	-7.4	-6.8
**mb 2.4**	-5.0	-1.6	-4.4	-3.6	-4.9	-4.3	-1.2	-1.1	-7.7	-7.3
**mb 3.2**	-1.9	-0.4	-1.5	-0.8	-1.8	-1	2.2	-0.03	-4.1	-4.5
**mb 3.3**	-1.8	-0.3	-1.3	-0.6	-1.7	-0.9	2.4	0.06	-3.9	-3.7
**mb 3.4**	-1.7	-0.5	-1.6	-1.4	-2.1	-1.3	1.6	-0.8	-3.9	-3.7
**mb 4.1**	-5.5	-1.2	-4.2	-3.1	-5.6	-5.2	-3.2	-3.5	-6.6	-7.2
**mb 4.2**	-5.2	-1.3	-4.3	-3.2	-5.3	-4.5	-1.6	-1.8	-7.3	-7.1
**mb 4.3**	-4.98	-1.3	-4.6	-3.3	-4.7	-3.9	-2.1	-2.3	-7.0	-6.6
**mb 5.1**	-5.05	-1.9	-4.6	-3.3	-4.9	-4.5	-2.0	-2.2	-6.3	-6.9
**mb 5.2**	-4.9	-2	-4.3	-3.2	-4.7	-4.3	-2.2	-2.4	-6.6	-6.4
**mb 5.3**	-3.2	-1.1	-3.8	-3.1	-3.1	-2.6	-0.3	-0.8	-5.0	-5.4
**mb 5.4**	-4.2	-0.3	-4.1	-3.2	-4.3	-3.8	-0.7	-2.1	-6.5	-6.2
**TDMACL**	0.2	0.2	-0.3	-0.85	-0.1	0.06	2.2	1.5	-2.7	-2.9

Table S3. Selectivity coefficients of different membrane composition against various interfering ions of membranes based on **L1−L5** ligands with varied amount of TDMACl anion exchanger (compositions listed in Table 1) for KF^- as primary ion. .Data reported graphically in Figure S26.

	Cl^-	NS^-	$Benz^-$	IB^-	Br^-	NO_3^-	ClO_4^-	SCN-	CO_3^{2-}	SO_4^{2-}
**mb 1.1**	-3.3	1.7	-1.5	-0.13	-3.4	-2.98	-0.5	-1.02	-5.1	-5.1
**mb 1.2**	-2.9	1.8	-1.5	-0.13	-2.9	-2.77	-0.6	-1.2	-5.4	-5.96
**mb 1.3**	-2.6	1.6	-1.5	-0.14	-2.4	-2.1	0.9	-0.005	-5.4	-5.1
**mb 1.4**	-2.4	2.5	-2.5	-1.1	-3.4	-1.6	1.9	0.13	-5.8	-5.2
**mb 2.4**	-3.4	1.6	-2.8	-1.9	-3.3	-2.7	0.4	0.53	-6.7	-6.3
**mb 3.2**	-1.5	0.4	-1.14	-0.5	-1.5	-0.6	2.6	0.34	-	-
**mb 3.3**	-1.5	0.3	-1.11	-0.4	-1.4	-0.7	2.7	0.34	-	-
**mb 3.4**	-1.2	0.5	-1.1	-0.9	-1.5	-0.8	2.1	-0.27	-	-
**mb 4.1**	-4.2	1.2	-2.7	-1.5	-4.3	-3.9	-2.1	-2.1	-4.6	-5.1
**mb 4.2**	-3.9	1.3	-2.9	-1.6	-3.9	-3.2	-0.5	0.58	-5.1	-4.9
**mb 4.3**	-3.6	1.3	-3.3	-1.95	-3.3	-2.6	-0.84	-0.94	-4.9	-4.6
**mb 5.1**	-3.2	1.9	-2.7	-1.2	-3.1	-2.7	-0.1	-2.1	-5.6	-6.2
**mb 5.2**	-2.9	2.0	-2.6	-1.24	-2.8	-2.3	-0.2	-0.44	-4.8	-4.7
**mb 5.3**	-2.2	1.1	-2.5	-1.21	-2.01	-1.6	0.7	0.31	-4.6	-5.0
**mb 5.4**	-3.9	0.3	-2.6	-1.26	-3.99	-3.5	-0.4	-1.75	-6.5	-6.2
**TDMACL**	-0.3	-0.2	-0.5	-0.85	-0.98	-0.17	2.01	1.26	-4.7	-4.5

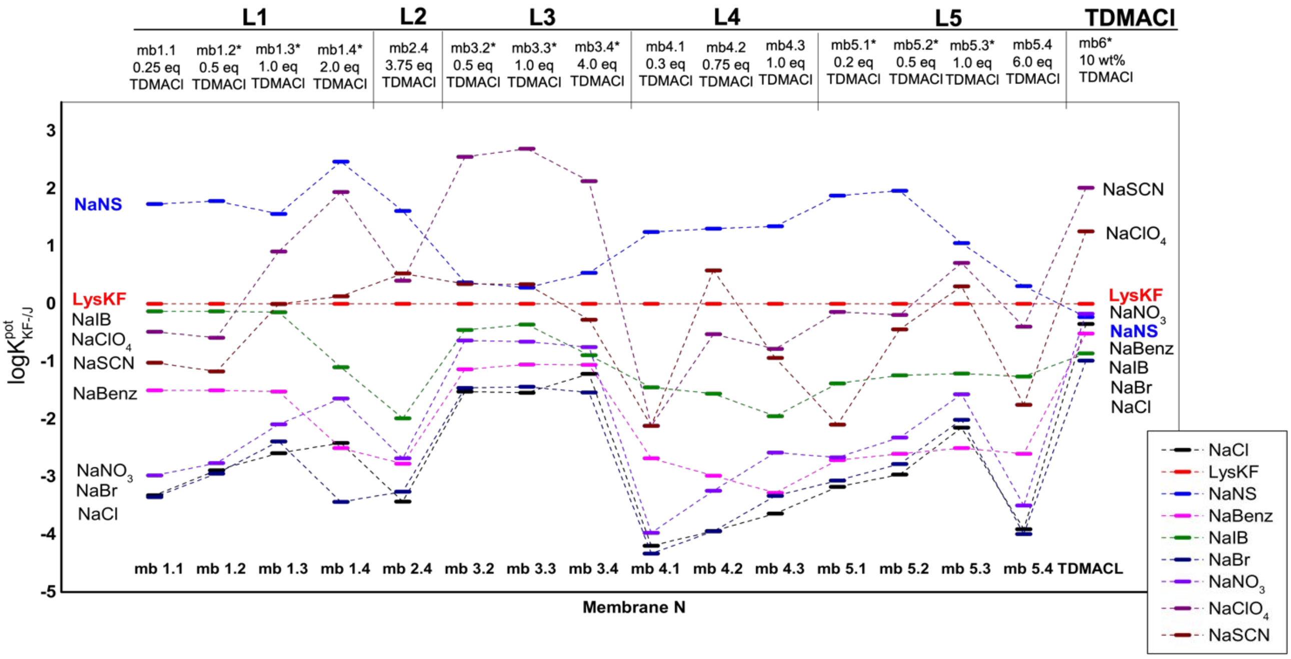

Figure S26. Potentiometric selectivity coefficients of membranes based on **L1**–**L5** ligands with varied amount of TDMACl anion exchanger (indicated in eq. relative to ligand content) for KF^- as primary ion. The values of $\log K^{pot}_{I/J}$ were estimated with SSM method, the slope of −59.2 mV/dec was used for calculus; the membranes non-exhibiting Nernstian response are indicated with (*) mark. For comparison, the selectivity of mb 6, formulated only with 10 wt% of TDMACl is also shown.

[1] Marchetti L A, Kumawat L K, Mao N, Stephens J C, Elmes R B P, Chem., 5 (2019) 1398-1485.
